# Effects of exercise and liraglutide on vascular health and inflammation during weight loss maintenance: a prespecified secondary analysis of the S-LiTE trial

**DOI:** 10.1038/s42255-026-01554-4

**Published:** 2026-06-24

**Authors:** Rasmus Michael Sandsdal, Joachim Holt, Haithem Ghalib Ali Alkhefagie, Julie Rehné Jørgensen, Christian Rimer Juhl, Lisa M. Olsen, Sarah Byberg, Roopameera Thirumathyam, Lasse Gliemann, Bente Stallknecht, Jens Juul Holst, Jens Dahlgaard Hove, Thomas Bandholm, Kirstine Nyvold Bojsen-Møller, Sten Madsbad, Joachim Størling, Else Marie Bladbjerg, Charalambos Antoniades, Simon Birk Kjær Jensen, Signe Sørensen Torekov

**Affiliations:** 1 Department of Biomedical Sciences, Faculty of Health and Medical Sciences, University of Copenhagen, Copenhagen, Denmark; 2 Department of Endocrinology, Copenhagen University Hospital Hvidovre, Hvidovre, Denmark; 3 The August Krogh Section for Human Physiology, Department of Nutrition, Exercise and Sports, University of Copenhagen, Copenhagen, Denmark; 4 NNF Center for Basic Metabolic Research, Faculty of Health and Medical Sciences, University of Copenhagen, Copenhagen, Denmark; 5 Department of Cardiology, Copenhagen University Hospital Hvidovre, Hvidovre, Denmark; 6 Physical Medicine and Rehabilitation Research - Copenhagen (PMR-C), Department of Physical and Occupational Therapy, Copenhagen University Hospital Hvidovre, Hvidovre, Denmark; 7 Department of Clinical Medicine, University of Copenhagen, Copenhagen, Denmark; 8 Translational Type 1 Diabetes Research, Clinical Research, Steno Diabetes Center Copenhagen, Herlev, Denmark; 9 Unit for Thrombosis Research, Department of Clinical Biochemistry, University Hospital of Southern Denmark, Esbjerg, and Department of Regional Health Research, University of Southern Denmark, Odense, Denmark; 10 Acute Multidisciplinary Imaging and Interventional Centre, Radcliffe Department of Medicine, University of Oxford, Oxford, UK

**Keywords:** Risk factors, Predictive markers, Translational research, Metabolism

## Abstract

Obesity and inactivity are linked to endothelial dysfunction and atherosclerosis. In this secondary analysis of the S-LiTE trial (ClinicalTrials.gov identifier: NCT04122716; EudraCT identifier: 2015–005585-32), 130 adults with obesity completed a diet-induced weight loss plan, followed by randomization to weight maintenance with exercise and/or liraglutide for 52 weeks. We show that exercise, alone or in combination with liraglutide, reduces carotid intima-media thickness and systemic pro-inflammatory cytokine levels (interleukin-6 and interferon-γ). Combination treatment also improves endothelial function biomarkers (sICAM-1, sVCAM-1 and tPA). Liraglutide alone shows no such improvements. Overall, regular physical activity, with or without GLP-1R agonists, is essential for promoting vascular health in adults with obesity.

## Abstract (web summary)

Exercise, alone or in combination with liraglutide, reduces vascular thickness and inflammation markers during the weight loss maintenance phase, whereas liraglutide alone does not elicit any effects.

## Main

Obesity and physical inactivity are major contributors to the global burden of cardiovascular disease^1,2^. Excess adiposity promotes chronic, low-grade systemic inflammation that contributes to endothelial dysfunction and vascular remodelling^3,4^. Carotid intima-media thickness (cIMT), measured non-invasively by ultrasound, serves as a surrogate marker of vascular health and is associated with increased cardiovascular risk^5,6^. Both exercise and glucagon-like peptide-1 receptor agonists (GLP-1 RAs) individually seem to promote cardiovascular health^7–10^. However, mechanisms by which exercise and GLP-1 RAs ameliorate cardiovascular risk after weight loss remain to be determined. Therefore, we aimed to determine the effects of exercise and GLP-1 RA liraglutide, either alone or in combination, on cIMT and circulating pro-inflammatory cytokines and endothelial function in a prespecified secondary analysis of the S-LiTE randomized placebo-controlled trial^11^ to elucidate distinct mechanisms on vascular health.

**Extended Data Fig. 1:**
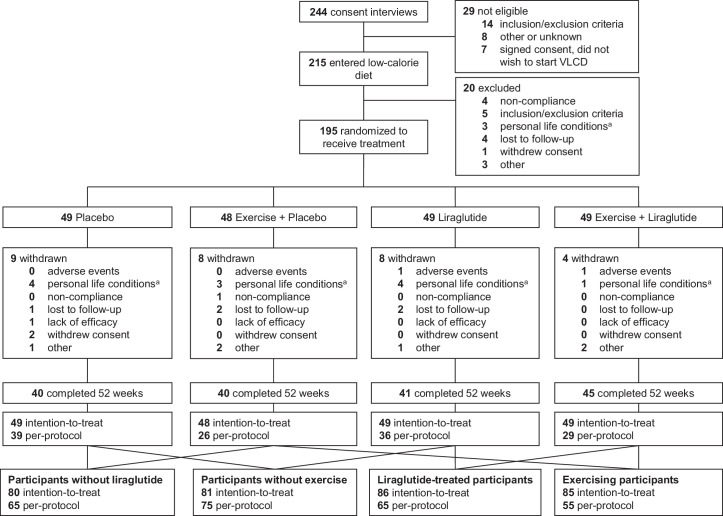
CONSORT study flow chart. CONSORT flow diagram of the participants enrolled in the S-LiTE trial. ^a^personal life conditions could be job-related, moving, or death in the family.

To standardize nutritional and metabolic status at randomization and reduce variation in circulating biomarkers attributable to dietary factors alone, participants in the S-LiTE trial were required to complete an 8 week low-calorie diet (LCD) to lose at least 5% of their body weight. In total, 130 adults with obesity (body mass index of 32–43 kg m^−2^) without diabetes completed the LCD and lost an average of 13.7 kg (12.7%) of total body weight; these individuals were then randomized to 52 weeks of treatment with placebo, exercise, liraglutide (3.0 mg per day) or exercise combined with liraglutide (Table 1, Extended Data Fig. 1 and Supplementary Table 1). After 1 year, individuals in the placebo group had regained weight, individuals in both the exercise and the liraglutide groups maintained their diet-induced weight loss, while the combination group experienced a further reduction in body weight. This observation enabled us to investigate weight-loss-independent effects of exercise or liraglutide treatment alone. Total weight loss across the duration of the trial is presented in Supplementary Table 2. Exercise adherence was 156 ± 54 min per week at 78 ± 4% maximum heart rate in the exercise-only group (corresponding to 92% achieving the minimum World Health Organization (WHO) recommendations on physical activity) and 144 ± 67 min per week at 78 ± 5% maximum heart rate in the combination group (corresponding to 79% achieving the minimum WHO recommendations on physical activity). The mean daily medication dose during the study was ≥2.6 mg across all groups.

**Table 1:** Baseline characteristics

	Placebo (*n* = 39)	Exercise (*n* = 26)	Liraglutide (*n* = 36)	Combination (*n* = 29)
Sex^a^, *n* (%) male / female	15 (38) / 24 (62)	11 (42) / 15 (58)	13 (36) / 23 (64)	11 (38) / 18 (62)
Age (years)	43.7 ± 12.3	45 ± 11.8	46.2 ± 10.3	44.5 ± 12.7
Body mass index (kg m^−2^)	32.1 ± 2.9	32.2 ± 3.3	32.5 ± 3.1	32.6 ± 2.5
Systolic blood pressure (mmHg)	122 ± 15	125 ± 17	122 ± 11	121 ± 13
Diastolic blood pressure (mmHg)	79 ± 8	78 ± 9	80 ± 8	77 ± 8
Total cholesterol (mmol l^−1^)	4.1 ± 0.8	3.8 ± 0.9	4.3 ± 0.8	3.8 ± 1.0
LDL cholesterol (mmol l^−1^)	2.5 ± 0.7	2.2 ± 0.8	2.7 ± 0.7	2.2 ± 0.9
HDL cholesterol (mmol l^−1^)	1.1 ± 0.2	1.2 ± 0.2	1.1 ± 0.3	1.1 ± 0.3
Triglycerides (mmol l^−1^)	1.0 (0.8–1.3)	0.9 (0.7–1.1)	0.9 (0.7–1.1)	1.1 (0.9–1.3)
Vascular ultrasound assessment				
cIMT (mm)	0.57 (0.52–0.65)	0.59 (0.54–0.70)	0.55 (0.51–0.61)	0.57 (0.49–0.61)
Inflammatory markers				
IL-6 (pg ml^−1^)	1.0 (0.7–1.4)	1.1 (0.9–1.6)	1.0 (0.8–1.5)	1.2 (1.0–1.8)
IL-8 (pg ml^−1^)	7.2 (6.0–8.9)	7.1 (6.2–7.9)	7.1 (5.7–9.4)	7.5 (6.0–9.8)
IL-10 (pg ml^−1^)	0.3 (0.2–0.3)	0.3 (0.2–0.5)	0.3 (0.2–0.4)	0.3 (0.2–0.4)
IFNγ (pg ml^−1^)	6.4 (3.8–10.8)	7.0 (5.0–18.5)	6.6 (3.7–9.6)	6.3 (5.1–10.2)
TNF (pg ml^−1^)	1.3 (1.0–1.6)	1.2 (0.9–1.5)	1.2 (1.0–1.4)	1.4 (1.2–1.6)
Endothelial markers				
sICAM-1 (ng ml^−1^)	0.5 (0.5–0.7)	0.6 (0.4–0.8)	0.5 (0.4–0.7)	0.6 (0.4–0.8)
sVCAM-1 (ng ml^−1^)	0.6 (0.5–0.7)	0.6 (0.5–0.7)	0.6 (0.5–0.7)	0.7 (0.5–0.8)
tPA (ng ml^−1^)	8.9 (7.2–13.4)	8.4 (6.3–9.9)	9.1 (6.9–12.3)	10.7 (6.7–14.4)
vWF (%)	126 ± 42	118 ± 41	130 ± 47	121 ± 38

Baseline characteristics at randomization (after the LCD) of the per-protocol population (*n* = 130). Data presented as mean ± s.d. or median (interquartile range). cIMT, carotid intima-media thickness; LDL, low-density lipoprotein; HDL, high-density lipoprotein.

^a^Sex assigned at birth.

**Fig. 1:**
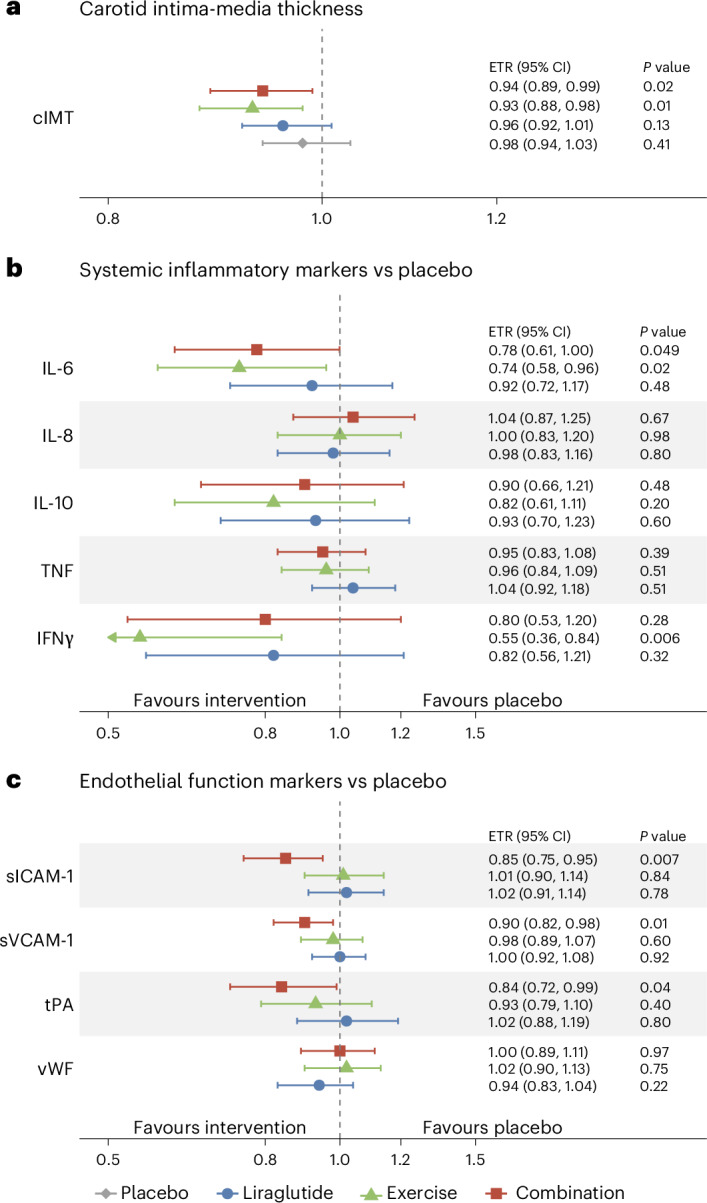
Effects of exercise (*n* = 26), liraglutide (*n* = 36) and the combination (exercise + liraglutide, *n* = 29) on vascular structure and circulating biomarkers during weight loss maintenance. **a**, Changes in cIMT in the four intervention groups during the 1 year intervention. **b**, Changes in inflammation markers compared with placebo (*n* = 39) during the 1 year intervention. **c**, Changes in endothelial function markers compared with placebo during the 1 year intervention. Data are presented as estimated treatment ratios (ETR) with 95% confidence intervals (CI), derived from a linear mixed model for repeated measurements, with sex assigned at birth, age (<40 vs ≥40 years), treatment, time (factorial) and a time × treatment interaction as covariates and an unstructured covariance pattern in the per-protocol population (*n* = 130). For cIMT, the analysis was adjusted for baseline scans. Analysis was performed on a log scale to meet model assumptions. All statistical tests were two-sided. No adjustment for multiple comparisons was applied. Source data

To assess the effects of exercise and liraglutide on vascular health, we quantified intimal thickening in the carotid artery by measuring the intima-media thickness using standardized ultrasound^5,12^. No changes in cIMT were observed during LCD-induced weight loss (Supplementary Table 1). During the 52 week weight maintenance phase, cIMT decreased in the exercise-containing groups, with reductions of 7% in the exercise group (95% CI, 2 to 12; *P* = 0.01) and 6% in the combination group (95% CI, 1 to 11; *P* = 0.02) (Fig. 1 and Supplementary Table 3). The change in cIMT (~0.04 mm) in this study is within the range reported in meta-analyses as being associated with cardiovascular risk reduction^6^; however, whether such a change indeed translates into clinical benefit remains uncertain. No changes were observed with liraglutide-only treatment.

We then investigated the effects of exercise, liraglutide or the combination treatment on the systemic pro-inflammatory cytokines interleukin (IL)-6, tumour necrosis factor (TNF) and interferon-γ (IFNγ), the most upstream circulating markers of atherosclerotic development^13–16^. We found that the initial weight loss induced a 10% increase in TNF, with no change in IL-6 or IFNγ, suggesting a transient increase in inflammation during rapid weight loss (Supplementary Table 1). After 1 year of treatment, IL-6 declined by 26% (4 to 42; *P* = 0.02) in the exercise group and by 22% (0.1 to 39; *P* = 0.049) in the combination group compared with placebo (Fig. 1 and Supplementary Table 3). Furthermore, IFNγ was reduced by 45% (23 to 60; *P* = 0.006) in the exercise group compared with placebo. No effects were observed after 1 year of liraglutide treatment compared with placebo.

Increased pro-inflammatory cytokines, such as IL-6 and IFNγ, are partly derived from adipose tissue^17,18^ and can induce the expression of markers of endothelial dysfunction, providing a mechanistic link between obesity-related inflammation and atherogenesis. Therefore, we assessed endothelial function markers associated with increased atherosclerotic development, including intercellular adhesion molecule 1 (sICAM-1), vascular adhesion molecule 1 (sVCAM-1), tissue plasminogen activator (tPA) and von Willebrand factor (vWF)^19–22^. The LCD-induced weight loss led to decreases of 14% in sICAM-1, 5% in vWF and 19% in tPA, and an 8% increase in sVCAM-1 (Supplementary Table 1). After 1 year of treatment, only the combination group had reductions in sICAM-1 and sVCAM-1, by 13% (5 to 20; *P* = 0.007) and 15% (9 to 20; *P* = 0.01), respectively, and tPA by 16% (1 to 28; *P* = 0.04) compared with placebo (Fig. 1 and Supplementary Table 3). However, 1 year of liraglutide treatment alone was not associated with improvements in endothelial function markers. At inclusion, cIMT was positively associated with blood pressure, but changes in cIMT during the 1 year treatment were not associated with changes in blood pressure or biomarkers (Supplementary Table 4).

**Extended Data Fig. 2:**
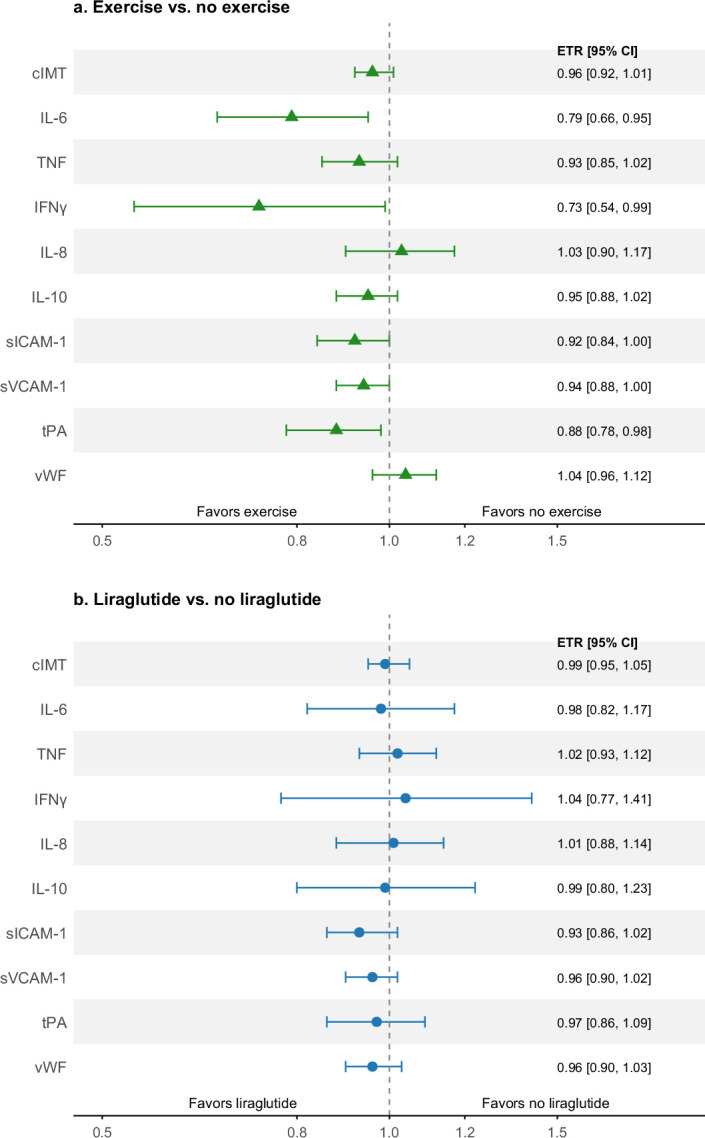
Changes in cIMT and biomarkers according to the 2×2 study design. Effects of exercise (exercise + placebo group and exercise + liraglutide group, n = 55) vs. no exercise (placebo + usual activity and liraglutide + usual activity group, n = 75) in Panel **a** and liraglutide (liraglutide + usual activity group and exercise + liraglutide, n = 65) vs no liraglutide (placebo + usual activity and exercise + placebo group, n = 65) in Panel **b** on vascular structure and circulating biomarkers during weight loss maintenance. Estimated treatment ratios (ETR) are derived from a linear mixed model for repeated measurements with sex assigned at birth, age ( < 40 vs ≥40 years), treatment (exercise vs. no exercise, and liraglutide vs. no liraglutide), time (factorial), and a time×treatment interaction as covariates and an unstructured covariance pattern in the per-protocol population (n = 130). Analysis was performed on log scale to meet model assumptions. Estimates are back-transformed and presented as estimated treatment ratios with 95% confidence intervals. For cIMT, the analysis was adjusted for baseline scans. All statistical tests were two-sided. No adjustment for multiple comparisons was applied. ETR: Estimated treatment ratio. IL-6: Interleukin-6, TNF: Tumor necrosis factor alpha, IFNγ: Interferon-γ, sICAM-1: soluble Intercellular adhesion molecule 1, sVCAM-1: soluble vascular cellular adhesion molecule 1, tPA: tissue plasminogen activator. Source data

Our findings underscore the importance of incorporating and adhering to physical activity as an adjunct to GLP-1-based therapy for obesity, as it promotes healthy weight maintenance with reduced levels of the pro-inflammatory cytokines IL-6 and IFNγ and improved endothelial function markers sICAM-1, sVCAM-1 and tPA. Together with the observed decrease in cIMT captured by ultrasound, our findings suggest that structural vascular adaptations are more sensitive to exercise-induced haemodynamic and anti-inflammatory effects than to the effects of liraglutide on metabolism alone during weight loss maintenance^11^. Our findings are supported by pooled analyses of the two interventions (exercise or liraglutide); that is, exercising versus non-exercising participants and liraglutide-treated versus non-liraglutide-treated participants (Extended Data Fig. 2).

We found that 1 year of treatment with liraglutide did not reduce cIMT while maintaining the diet-induced weight loss. This contrasts with previous clinical trials showing cIMT reduction with GLP-1 RA treatment. However, the previous studies examined patients with type 2 diabetes, a population characterized by substantially higher baseline cIMT, and did not include an initial standardized weight loss phase^23–25^. In addition, the large cardiovascular outcome trial with the GLP-1 RA semaglutide in patients with established cardiovascular disease has shown reduced incidence of cardiovascular events^10^. Our findings should be interpreted as pertaining to the weight loss maintenance phase in people with obesity who do not have diabetes or established cardiovascular disease and have already achieved substantial weight loss. In this context, liraglutide did not provide additional benefit in reducing cIMT and pro-inflammatory cytokines without exercise.

Strengths of this analysis include standardization of diet and weight loss through an LCD run-in, the randomized four-group design enabling evaluation of both independent and combined interventions, high adherence to exercise and study medication and comprehensive profiling using minimally invasive vascular imaging and emerging pro-atherosclerotic biomarkers. Limitations should be considered. This is a prespecified secondary analysis, and findings should be interpreted with appropriate caution regarding type I error. The ultrasound assessments were performed by personnel trained under a protocol that yielded a low coefficient of variation (around 5%) before the initiation of the trial. Additionally, cIMT is a surrogate endpoint regarding vascular health^6^. Although initial diet-induced weight loss is used in some clinical practices, including in Denmark and the UK^26–28^, GLP-1-based therapy is commonly prescribed as an adjunct to diet and exercise recommendations. Therefore, in this study, the initial weight loss may have attenuated the weight-loss-dependent effects of liraglutide and exercise. However, the main outcome, cIMT, did not improve with the LCD-induced weight loss, but only with 1 year of exercise with or without liraglutide. Although liraglutide is a first-generation GLP-1 RA with modest weight loss effects compared with newer agents^29^, liraglutide was administered at the highest approved dose and maintained a large weight loss. Participants in the exercise groups had more frequent contact with study personnel than those in the placebo and liraglutide groups, which could have led to a Hawthorne effect; nevertheless, adherence to study medication was similar across all groups. The partly supervised exercise intervention helped ensure adequate intensity and volume for mechanistic evaluations but may limit direct generalizability to routine clinical settings. The intervention had a duration of 1 year, and it is uncertain whether the observed results are sustainable in the long term; however, a follow-up study of the S-LiTE trial showed that the exercising groups had better maintenance of body weight and body composition 1 year after termination of the supervised exercise programme, whereas weight gain was observed after cessation of liraglutide treatment alone^30^.

In conclusion, among individuals with obesity who had adhered to a low-calorie diet, exercise reduced cIMT and pro-inflammatory cytokine levels, and the combination of exercise and liraglutide also improved endothelial function. This was not observed with liraglutide treatment alone or with placebo, highlighting the role of regular physical activity in improving vascular health in adults with obesity.

## Methods

### Study design

This study is a secondary analysis of a randomized, placebo-controlled trial. The trial is registered with EudraCT (2015–005585-32) and ClinicalTrials.gov (NCT04122716). The trial was conducted at Copenhagen University Hospital Hvidovre and the University of Copenhagen, Denmark. The study protocol and statistical analysis plan have been published and are also provided with this paper^11,31^. Results concerning the primary endpoint (change in body weight) and a secondary endpoint (change in body fat percentage) have been previously published^11^.

The trial protocol was approved by the Regional Ethics Committee for the Capital Region of Denmark (H-16027082) and the Danish Medicines Agency. The study was conducted in accordance with the Declaration of Helsinki and under the oversight of ICH Good Clinical Practice. All participants provided written informed consent before enrolment. Participants who completed the trial received compensation of 3,000 Danish kroner.

All participants initially followed an 8 week LCD (800 kcal d^−1^). During this phase, all meals were substituted with four formula meal replacement products daily, according to the Cambridge Weight Plan. On completion of the 8 week LCD, only participants who lost at least 5% of their initial body weight were eligible for enrolment and subsequently randomized in this study. Participants were randomly allocated to one of four 52 week interventions: placebo with usual activity, exercise with placebo, liraglutide with usual activity or a combination of exercise and liraglutide, in a 1:1:1:1 ratio to one of four treatment arms using a computer-generated randomization sequence provided by Novo Nordisk (subject randomization list; see study flow, Extended Data Fig. 1). The allocation sequence was stratified by sex and age, with separate randomization lists for participants aged ≥40 years and <40 years. Within each stratum, participants were assigned sequentially according to the randomization list. Allocation was implemented by a qualified, unblinded study nurse who was not otherwise involved in trial conduct. Study participants and study personnel were blinded to the study medication. Sex was defined as assigned at birth and was registered by a physician in an electronic case report form.

We also explored the main effects of the two interventions (exercise and liraglutide) on the investigated outcomes by testing each intervention independently against its respective control condition within the factorial design of the trial: exercise versus no exercise and liraglutide versus no liraglutide.

### Study participants

Participants were adults with obesity (18–65 years of age, body mass index of 32–43 kg m^−^^2^). Major exclusion criteria were any known serious chronic illness, including type 1 or 2 diabetes. A full list of inclusion and exclusion criteria is available in the protocol paper^31^. Use of blood pressure and lipid-lowering medication, as well as prior and current tobacco use, is reported in Supplementary Table 5.

### Exercise intervention

The exercise intervention was designed to align with WHO recommendations for physical activity, aiming for at least 150 min of moderate-intensity or 75 min of vigorous-intensity aerobic exercise each week, or an equivalent combination. To achieve this target, participants were encouraged to complete two supervised group sessions and two individual exercise sessions each week. Each 45 min group session included 30 min of vigorous, interval-based indoor cycling, targeting ≥80% of maximum heart rate, followed by 15 min of circuit training. The circuit training typically comprised three rounds of five exercises, each performed for 40 s with 20 s rest intervals, combining high-intensity aerobic and resistance exercises using body weight and/or external weights. Individual sessions were self-directed and involved moderate-to-vigorous aerobic activities such as cycling, running, brisk walking or circuit training, with intensity monitored through heart rate sensors. To enhance adherence, several support strategies were implemented: monthly or bimonthly in-person weight consultations to review exercise data from sports watches and address participation barriers; monthly feedback emails providing personalized summaries and motivational prompts; and individualized adherence plans for those not meeting goals, which could include increasing non-exercise physical activity, adjusting the balance of group and individual sessions or temporarily reducing frequency with a plan to increase it gradually. Full details of the intervention, in accordance with the Consensus on Exercise Reporting Template (CERT), are provided in the supplementary appendix, methods, section D of the primary trial report^11^.

### Pharmacological intervention

Participants received either liraglutide or volume-matched placebo, each containing 3 ml of solution (6 mg ml^−1^). Injections were administered once daily into the abdomen or thigh, starting with a dose of 0.6 mg d^−1^ and increasing weekly by 0.6 mg until a maximum of 3.0 mg d^−1^ was reached. If participants experienced intolerance to a given dose, they continued with the maximum tolerated dose.

### Outcomes

The present analyses were conducted as part of a predefined plan to investigate cardiometabolic risk modulation during exercise and GLP-1-based obesity therapy, using imaging and blood samples. The prespecified main outcome in this analysis was the change in cIMT^11^ in persons with obesity after 52 weeks of weight maintenance with exercise and liraglutide treatment. Supportive outcomes were changes during the weight maintenance period in markers of general inflammation (IL-6, IL-8, IL-10, TNF and IFNγ) and endothelial function (sICAM-1, sVCAM-1, vWF and tPA), which were prespecified objectives in the trial protocol^11^. Safety outcomes have previously been reported^11^. Trial data were collected and managed using the electronic case report form REDCap (Research Electronic Data Capture).

### cIMT

cIMT provides a validated, reproducible and non-invasive measure of subclinical atherogenic burden that is particularly informative in asymptomatic, middle-aged individuals with obesity and without previously established atherosclerotic disease^5^. Assessments were performed by trained personnel and in accordance with a previously described method^12^, which provides a coefficient of variation of 5.1%. Examinations were performed using a Philips CX50 ultrasound system and a high-frequency linear vascular probe, Philips L12-3 (IP X-7) with an internal electrocardiography monitor. Carotid ultrasound images were acquired in a supine position with the person at rest. The images were recorded approximately 1 cm caudally from the right carotid bulb. The right common carotid artery cIMT was measured by two blinded assessors at three evenly spaced locations on the image frame of the far carotid artery wall obtained at the R wave of the accompanying electrocardiography recording and reported as the average of these values. Blinded analyses were performed using ultrasound quantification software (QLAB v.15, Philips Healthcare).

### Biomarkers and blood pressure

Blood samples were collected after a minimum fasting period of 10 h and before the ultrasound examination of cIMT. The plasma concentration of vWF antigen (%) was determined by ELISA using rabbit anti-human vWF polyclonal IgG as capture and detection antibodies (DAKO, A0082)^32^. Results of vWF were expressed relative to a reference plasma (Biopool) calibrated against the WHO international standard for vWF. The concentration of tPA antigen (ng ml^−1^) was determined by an in-house ELISA using mouse anti-human tPA monoclonal IgG as capture (clone 15-4-21) and detection (clone 15-4-6) antibodies^32^. sICAM-1, sVCAM-1 and high-sensitivity CRP were analysed with a V-PLEX MSD MULTI-SPOT Assay System (Vascular Injury Panel 2 (human) Kits, K15198D). Pro-inflammatory cytokines were measured in duplicates using a V-PLEX MSD Proinflammatory Panel 1 (human), K15049D, which assesses ten cytokines. However, only IL-6, IL-8, IL-10, TNF and IFNγ displayed detection rates above 75%; therefore, only these five cytokines were included in the analyses, which is typical for this assay in a population without specific inflammatory diseases^33^. No measurements of the five cytokines reported had measurements below the detection limit; see Supplemental Table 6 for a table of the coefficient of variation and limits of detection and quantification of the measured biomarkers. Office blood pressure was measured twice in a seated position after at least 5 min of rest with an automated sphygmomanometer, and the average of the recordings is provided.

### Statistical analysis and figures

Descriptive data are presented as mean and standard deviation if normally distributed or median with interquartile range if not normally distributed. Changes in outcomes were analysed using a linear mixed model for repeated measurements with time (factorial), treatment group (placebo, exercise, liraglutide or exercise + liraglutide), time × treatment interactions, age (<40 years or ≥40 years) and sex as fixed effects, as prespecified in the statistical analysis plan for the original trial^11^. For serial imaging, the baseline value (at randomization) was included as a covariate. Participants were stratified by age and sex; therefore, these variables were included as covariates in the model, as recommended by the European Medicines Agency. The model assumed an unstructured covariance and a repeated effect for time. Model fit was visually inspected, and if residuals were not normally distributed, analyses were performed on log-transformed data. For data presentation, log-transformed data were back-transformed and presented as percentage change with 95% confidence intervals. All analyses were performed using SAS Enterprise Guide 8.1. The analyses were performed in the per-protocol population (defined as participants who performed at least 75% of the exercise programme and/or had taken at least 2.4 or 3.0 mg d^−1^ of liraglutide or placebo for at least 75% of the intervention period). Data from the intention-to-treat population are shown in Supplementary Table 7 and generally support the main findings. All missing data were assumed to be missing at random and handled implicitly in the mixed model by maximum likelihood estimation. As an exploratory analysis, the 2 × 2 factorial design was used to examine the pooled effects of exercise and liraglutide and their potential interaction. In the original trial, it was estimated that 30 participants per group needed to be included to detect a 4 kg difference in weight between the intervention groups, with 80% power at a significance level of 5% (ref. ^11^). Significance testing was performed on the outcomes cIMT and biomarkers (IL-6, IL-8, IL-10, TNF, IFNγ, tPA, vWF, sICAM-1 and sVCAM-1). *P* < 0.05 was considered statistically significant. Figures were created in R (v.4.5.1) using the ggplot2 package (v.4.0.3).

### Reporting summary

Further information on research design is available in the Nature Portfolio Reporting Summary linked to this article.

## Supplementary information

Supplementary Information Supplementary Tables 1–7

Reporting Summary

Peer Review File

## Source data

Source Data Fig. 1 Statistical source data for visualization.

Source Data Extended Data Fig. 2 Statistical source data for visualization.

## Data Availability

Data files for the figures, trial protocol and statistical analysis plan are provided with this paper. De-identified data may be available for research collaboration, in accordance with local regulations and the General Data Protection Regulation, upon reasonable request to the corresponding author and will require the completion of a data processing agreement. Source data are provided with this paper.
