# Supplementary material for: Effects of exercise and liraglutide on vascular health and inflammation during weight loss maintenance: a prespecified secondary analysis of the S-LiTE trial
Source: Nat Metab. 2026 Jun 24;8(7):1483–8. doi: 10.1038/s42255-026-01554-4 (PMC13400309; doi:10.1038/s42255-026-01554-4)
Supplement: Supplementary file 1 — Supplementary Tables 1–7 [file 42255_2026_1554_MOESM1_ESM.pdf]

nature metabolism

Supplementary information

https://doi.org/10.1038/s42255-026-01554-4

Effects of exercise and liraglutide on vascular health and inflammation during weight loss maintenance: a prespecified secondary analysis of the S-LiTE trial

In the format provided by the authors and unedited

Table of Contents

Table of Contents 1

Supplementary Table 1. Participant characteristics and estimated changes during the low-calorie diet 2

Supplementary Table 2. Changes in body weight in percent from inclusion and baseline to end-of-trial 3

Supplementary Table 3. Changes in carotid intima media thickness and biomarkers according to the four-group design in the per-protocol population 3

Supplementary Table 4. Association of change in biomarkers with change in cIMT ... 4

Supplementary Table 5. Blood pressure or lipid-lowering medication and smoking status at inclusion by randomization group 4

Supplementary Table 6. Assay performance on measured biomarkers 4

Supplementary Table 7. Changes in carotid intima media thickness and biomarkers according to the four-group design in the intention-to-treat population 5

Supplementary Table 1. Participant characteristics and estimated changes during the low-calorie diet

	**Before low-calorie diet** (n=130)	**After low-calorie diet** (n=130)	**Estimated changes**
**Sex assigned at birth, n male/female, (%)**	50/80 (38%/62%)	50/80 (38%/62%)	
**Age (years)**	45±12	45±12	
**Body mass index (kg/m²)**	36.9±2.9	32.4±2.8	-4.5 (-4.8; -4.3)
**Body weight (kg)**	110.2±14.7	96.5±12.3	-13.7 (-14.5; -12.9)
**Systolic blood pressure, mmHg**	133±17	122±14	-10 (-13; -8)
**Diastolic blood pressure, mmHg**	87±9	79±8	-8 (-9; -7)
**Glycated hemoglobin, mmol/mol**	36±4	34±4	-2.2 (-2.7; -1.8)
**Total cholesterol, mmol/L**	5.0±1.0	4.0±0.9	-1.0 (-1.2; -0.9)
**Cholesterol LDL, mmol/L**	3.1±0.8	2.4±0.8	-0.6 (-0.8; -0.5)
**Cholesterol HDL, mmol/L**	1.3±0.3	1.1±0.2	-0.1 (-0.2; -0.1)
**Triglycerides, mmol/L**	1.2 [0.9; 1.8]	1.0 [0.8; 1.2]	-38% (-48; -27)
**Vascular ultrasound assessment**			
**Carotid intima-media thickness, mm**	0.59 [0.51; 0.68]	0.57 [0.51; 0.63]	0% (-4; 5)
**Inflammatory markers**			
**IL-6 (ρg/mL)**	1.2 [0.8; 1.6]	1.1 [0.8; 1.6]	-4% (-12; 4)
**IL-8 (ρg/mL)**	6.9 [5.8; 9.3]	7.2 [6.0; 9.0]	2% (-4; 9)
**IL-10 (ρg/mL)**	0.24 [0.17; 0.35]	0.26 [0.19; 0.41]	15% (3; 29)
**IFNγ (ρg/mL)**	6.6 [4.2; 10.9]	6.6 [4.3; 11.3]	10% (-4; 26)
**TNF (ρg/mL)**	1.2 [0.93; 1.3]	1.2 [0.99; 1.5]	10% (5; 14)
**hsCRP (pg/mL)[a]**	3.9 [1.6; 9.1]	2.4 [1.0; 5.8]	-29% (-39; -16)
**Endothelial markers**			
**sICAM-1, ng/mL**	0.63 [0.49; 0.82]	0.55 [0.39; 0.69]	-14% (-17; -11)
**sVCAM-1, ng/mL**	0.56 [0.48; 0.68]	0.62 [0.49; 0.74]	8% (5; 12)
**tPA, ng/mL**	11.1 [8.7; 14.6]	9.1 [6.9; 12.2]	-19% (-22; -15)
**vWF, %**	129±39	124±42	-5 (-9; -1)

Data are mean ± standard deviation for normally distributed data or median with interquartile range in brackets for non-normally distributed data. Results are from the 130 participants who completed the study and were adherent to the interventions (per-protocol population). Presented changes from before to after the low-calorie diet are estimates (with 95% confidence intervals) derived from a linear mixed model for repeated measurements with sex, age (above/below 40 years of age), and time (factorial) as covariates and an unstructured covariance pattern. Ultrasound, triglycerides, inflammatory and endothelial markers were analyzed on log-scale and back transformed for presentation. Analysis of cIMT included the value of cIMT at randomization as a covariate in the model.
[a]Outcome previously reported.

Supplementary Table 2. Changes in body weight in percent from inclusion and baseline to end-of-trial

	Placebo (n=39)	Exercise (n=26)	Liraglutide (n=36)	Combination (n=29)
**Inclusion to end-of-treatment (%)**	-7.1 (-9.6; -4.6)	-12.4 (-15.5; -9.3)	-14.4 (-17.1; -11.8)	-18.7 (-21.7; -15.8)
**Baseline to end-of-treatment (%)**	6.5 (3.8; 9.2)	0.7 (-2.6; 4.0)	-2.4 (-5.2; 0.4)	-6.3 (-9.5; -3.2)

Presented data are within-group changes (with 95% confidence intervals) in the per protocol population (n=130) from inclusion/baseline to end-of-treatment derived from a linear mixed model for repeated measurements with sex, age (above/below 40 years of age), treatment, time (factorial) and a time×treatment interaction as covariates and an unstructured covariance pattern.

Supplementary Table 3. Changes in carotid intima media thickness and biomarkers according to the four-group design in the per-protocol population

	Placebo (n=39)	Exercise (n=26)	Liraglutide (n=36)	Combination (n=29)
**cIMT[a, b]**	-2% (-6; 3)	-7% (-12; -2)	-4% (-8; 1)	-6% (-11; -1)
**vs. placebo**		-5% (-12; 2)	-2% (-8; 5)	-4% (-10; 3)
**IL-6[a]**	-11% (-24; 4)	-34% (-46; -20)	-18% (-32; -3)	-31% (-42; -17)
**vs. placebo**		-26% (-42; -4)	-8% (-28; 17)	-22% (-39; -0.1)
**TNF[a]**	-19% (-26; -12)	-23% (-30; -15)	-16% (-23; -8)	-24% (-31; -16)
**vs. placebo**		-4% (-16; 9)	4% (-8; 18)	-5% (-17; 8)
**IFNγ[a]**	1% (-23; 31)	-45% (-60; -23)	-18% (-38; 9)	-19% (-41; 9)
**vs. placebo**		-45% (-64; -16)	-18 (-44; 21)	-20% (-47; 20)
**IL-8[a]**	-26% (-34; -17)	-26% (-36; -15)	-27% (-36; -18)	-23% (-33; -12)
**vs. placebo**		0% (-17; 20)	-2% (-17; 16)	4% (-13; 25)
**IL-10[a]**	-9% (-25; 10)	-25% (-41; -6)	-16% (-31; 3)	-18% (-35; 2)
**vs. placebo**		-18% (-39; 11)	-7% (-30; 23)	-10% (-34; 21)
**hsCRP[a, c]**	-15% (-39; 20)	-18% (-45; 22)	-36% (-55; -8)	-52% (-67; -29)
**vs. placebo**		-4% (-43; 62)	-24% (-54; 24)	-43% (-66; -5)
**sICAM-1[a]**	3% (-5; 11)	4% (-5; 14)	5% (-4; 13)	-13% (-20; -5)
**vs. placebo**		1% (-10; 14)	2% (-9; 14)	-15% (-25; -5)
**sVCAM-1[a]**	-5% (-10; 1)	-7% (-13; -1)	-5% (-11; 0)	-15% (-20; -9)
**vs. placebo**		-2% (-11; 7)	0% (-8; 8)	-10% (-18; -2)
**tPA[a]**	9% (-2; 21)	2% (-11; 16)	11% (0; 24)	-8% (-19; 4)
**vs. placebo**		-7% (-21; 10)	2% (-12; 19)	-16% (-28; -1)
**vWF**	3% (-4; 11)	5% (-4; 14)	-3% (-11; 5)	3% (-5; 12)
**vs. placebo**		2% (-10; 13)	-6 (-17; 4)	0% (-11; 11)

Results according to the four-group design. Presented data are within-group changes and changes compared to placebo (with 95% confidence intervals) in the per protocol population (n=130) from randomization to end-of-treatment derived from a linear mixed model for repeated measurements with sex, age (above/below 40 years of age), treatment, time (factorial), and a time×treatment interaction as covariates and an unstructured covariance pattern. [a]Analysis was performed on a logarithmic scale and back transformed for presentation as percentage change. [b]Analysis of cIMT included the value of cIMT at randomization as a covariate in the model. [c]Outcome previously reported.

Supplementary Table 4. Association of change in biomarkers with change in cIMT

Parameter	Unstandardized β
**Tumor necrosis factor (TNF)**	-0.020 (-0.10; 0.06)
**Interferon-gamma (IFNγ)**	0.004 (-0.02; 0.03)
**Interleukin 6 (IL-6)**	0.02 (-0.02; 0.06)
**Interleukin 8 (IL-8)**	-0.06 (-0.13; 0.01)
**Interleukin 10 (IL-10)**	0.02 (-0.02; 0.07)
**Von Willebrand factor (vWF)**	0.11 (-0.02; 0.24)
**Tissue plasminogen activator (tPA)**	-0.002 (-0.08; 0.07)
**Soluble Intercellular adhesion molecule 1 (ICAM-1)**	0.03 (-0.17; 0.24)
**Soluble Vascular adhesion molecule 1 (VCAM-1)**	0.09 (-0.03; 0.22)
**Systolic blood pressure**	-0.0005 (-0.002; 0.001)
**Diastolic blood pressure**	-0.001 (-0.004; 0.001)

Association of change from baseline (week 0) to end-of-treatment (week 52) in outcomes (independent variable) with change from baseline (week 0) to end-of-treatment (week 52) in carotid intima-media thickness (cIMT, as dependent variable). Changes in cIMT and biomarkers are on a log scale. The analyses are adjusted for sex, age (above/below 40 years), and intervention group.

Supplementary Table 5. Blood pressure or lipid-lowering medication and smoking status at inclusion by randomization group

	Placebo	Exercise	Liraglutide	Combination
**Blood pressure medication, n (%)**	6 (15)	4 (16)	3 (8)	8 (28)
**Lipid-lowering medication, n (%)**	3 (8)	4 (16)	1 (3)	6 (20)
**Smoking at inclusion, n (%)**	6 (18)	3 (13)	4 (12)	2 (7)
**Previously smoked, n (%)**	12 (44)	7 (35)	12 (43)	16 (64)

Use of blood pressure or lipid-lowering medication and smoking status at inclusion by randomization group in the per-protocol population.

Supplementary Table 6. Assay performance on measured biomarkers

Variable	Inter-assay CV (%)	LLOD	LLOQ
**IL-6 (pg/mL)[a]**	7.3	0.06	0.63
**IL-8 (pg/mL)[a]**	7.1	0.07	0.59
**IL-10 (pg/mL)[a]**	10.1	0.04	0.30
**IFNγ (pg/mL)[a]**	7.3	0.37	1.8
**TNF (pg/mL)[a]**	10.1	0.04	0.69
**hsCRP (pg/mL)[a]**	9.9	1.33	27.6
**sVCAM (pg/mL)[a]**	8.4	6.00	37.6
**sICAM (pg/mL)[a]**	13.4	1.94	15.0
**tPA (ng/mL)**	5	0.31	0.75
**vWF (%)**	6	3.7	12.4

Inter-assay coefficient of variation in percent (CV) is the variability across different plates (usually 96-well plates) used for analyzing various biomarkers. LLOD: Lower limit of detection is defined as the calculated concentration corresponding to the average signal 2.5 standard deviations above background signaling. LLOQ: Lower limit of quantification; For [a]LLOQ is defined as the lowest concentration (of the control samples) where the CVs of the calculated concentration were below 20% and where recovery of the samples was within 80-120% of the known value of the standard across all used plates. For tPA and vWF, LLOQ was defined as the average signal 10 standard deviations above background signaling. [a]Numbers provided by the manufacturer of the kits (MSD).

Supplementary Table 7. Changes in carotid intima media thickness and biomarkers according to the four-group design in the intention-to-treat population

	Placebo (n=49)	Exercise (n=48)	Liraglutide (n=49)	Combination (n=49)
**cIMT[a,b]**	-3% (-7; 2)	-6% (-10; -1)	-3% (-7; 3)	-5% (-9; -1)
**vs. placebo**		-3% (-9; 3)	0 (-6; 6)	-3 (-9; 3)
**IL-6[a]**	-11% (-25; 5)	-32% (-43; -19)	-17% (-30; -2)	-20% (-32; -6)
**vs. placebo**		-23% (-40; -3)	-7 (-27; 18)	-10 (-28; 13)
**TNF[a]**	-20% (-26; -12)	-20% (-28; -13)	-17% (-24; -9)	-17% (-24; -9)
**vs. placebo**		-1% (-13; 13)	3% (-9; 17)	3 (-9; 17)
**IFNγ[a]**	0% (-24; 31)	-37% (-52; -17)	-9% (-30; 19)	-12% (-31; 15)
**vs. placebo**		-37% (-57; -7)	-9 (-38; 33)	-11% (-39; 29)
**IL-8[a]**	-25% (-33; -16)	-27% (-35; -17)	-29% (-37; -21)	-27% (-35; -18)
**vs. placebo**		-2 (-17; 15)	-6 (-20; 10)	-3 (-17; 14)
**IL-10[a]**	-9% (-25; 10)	-26% (-39; -11)	-17% (-31; 1)	-8% (-23; 11)
**vs. placebo**		-19% (-38; 6)	-8 (-30; 20)	-2 (-22; 33)
**sICAM-1[a]**	2% (-6; 12)	5% (-4; 14)	6% (-2; 15)	-5% (-12; 3)
**vs. placebo**		2 (-9; 15)	4 (-9; 17)	-7 (-17; 4)
**sVCAM-1[a]**	-5% (-11; 1)	-6% (-12; 0)	-4% (-10; 1)	-11% (-16; -6)
**vs. placebo**		-1 (-10; 8)	1 (-8; 10)	-6 (-14; 2)
**tPA[a]**	9% (-2; 21)	2% (-9; 14)	11% (0; 23)	-9% (-17; 1)
**vs. placebo**		-7 (-20; 9)	1 (-12; 17)	-16 (-28; -3)
**vWF**	3% (-4; 11)	2% (-5; 10)	-5% (-12; 3)	9% (2; 17)
**vs. placebo**		-1% (-12; 10)	-8% (-19; 3)	6% (-5; 16)

Results according to the four-group design. Presented data are within-group changes and changes compared to placebo (with 95% confidence intervals) in the intention-to-treat population (n=195) from randomization to end-of-treatment derived from a linear mixed model for repeated measurements with sex, age (above/below 40 years of age), treatment, time (factorial), and a time×treatment interaction as covariates and an unstructured covariance pattern. [a]Analysis was performed on a logarithmic scale and back transformed for presentation as percentage change. [b]Analysis of cIMT included the value of cIMT at randomization as a covariate in the model.

Section/topic	No	CONSORT 2025 checklist item description	Reported on page no.
**Title and abstract**			
Title and structured abstract	1a	Identification as a randomised trial	1
	1b	Structured summary of the trial design, methods, results, and conclusions	2
**Open science**			
Trial registration	2	Name of trial registry, identifying number (with URL) and date of registration	2 and 7
Protocol and statistical analysis plan	3	Where the trial protocol and statistical analysis plan can be accessed	Is included in the submitted documents
Data sharing	4	Where and how the individual de-identified participant data (including data dictionary), statistical code and any other materials can be accessed	11
Funding and conflicts of interest	5a	Sources of funding and other support (eg, supply of drugs), and role of funders in the design, conduct, analysis and reporting of the trial	11-12
	5b	Financial and other conflicts of interest of the manuscript authors	12
**Introduction**			
Background and rationale	6	Scientific background and rationale	3
Objectives	7	Specific objectives related to benefits and harms	Ref. 11
**Methods**			
Patient and public involvement	8	Details of patient or public involvement in the design, conduct and reporting of the trial	7-8
Trial design	9	Description of trial design including type of trial (eg, parallel group, crossover), allocation ratio, and framework (eg, superiority, equivalence, non-inferiority, exploratory)	7-8
Changes to trial protocol	10	Important changes to the trial after it commenced including any outcomes or analyses that were not prespecified, with reason	NA
Trial setting	11	Settings (eg, community, hospital) and locations (eg, countries, sites) where the trial was conducted	7 and Ref 31
Eligibility criteria	12a	Eligibility criteria for participants	8 and Ref. 31
	12b	If applicable, eligibility criteria for sites and for individuals delivering the interventions (eg, surgeons, physiotherapists)	Ref 31
Intervention and comparator	13	Intervention and comparator with sufficient details to allow replication. If relevant, where additional materials describing the intervention and comparator (eg, intervention manual) can be accessed	8-9
Outcomes	14	Prespecified primary and secondary outcomes, including the specific measurement variable (eg, systolic blood pressure), analysis metric (eg, change from baseline, final value, time to event), method of aggregation (eg, median, proportion), and time point for each outcome	9-10
Harms	15	How harms were defined and assessed (eg, systematically, non-systematically)	9 and Ref. 11
Sample size	16a	How sample size was determined, including all assumptions supporting the sample size calculation	11
	16b	Explanation of any interim analyses and stopping guidelines	NA
Randomisation:			
Sequence generation	17a	Who generated the random allocation sequence and the method used	Ref 31
	17b	Type of randomisation and details of any restriction (eg, stratification, blocking and block size)	7

			Reported on page no.
Allocation concealment mechanism	18	Mechanism used to implement the random allocation sequence (eg, central computer/telephone; sequentially numbered, opaque, sealed containers), describing any steps to conceal the sequence until interventions were assigned	7
Implementation	19	Whether the personnel who enrolled and those who assigned participants to the interventions had access to the random allocation sequence	7
Blinding	20a	Who was blinded after assignment to interventions (eg, participants, care providers, outcome assessors, data analysts)	7+9-10
	20b	If blinded, how blinding was achieved and description of the similarity of interventions	7 Ref 31
Statistical methods	21a	Statistical methods used to compare groups for primary and secondary outcomes, including harms	10-11
	21b	Definition of who is included in each analysis (eg, all randomised participants), and in which group	11 and Extended Data Figure 1
	21c	How missing data were handled in the analysis	11
	21d	Methods for any additional analyses (eg, subgroup and sensitivity analyses), distinguishing prespecified from post hoc	10-11
**Results**			
Participant flow, including flow diagram	22a	For each group, the numbers of participants who were randomly assigned, received intended intervention, and were analysed for the primary outcome	Extended Data Figure 1
	22b	For each group, losses and exclusions after randomisation, together with reasons	Extended Data Figure 1
Recruitment	23a	Dates defining the periods of recruitment and follow-up for outcomes of benefits and harms	Ref 11
	23b	If relevant, why the trial ended or was stopped	NA
Intervention and comparator delivery	24a	Intervention and comparator as they were actually administered (eg, where appropriate, who delivered the intervention/comparator, how participants adhered, whether they were delivered as intended (fidelity))	7-9
	24b	Concomitant care received during the trial for each group	Ref 31
Baseline data	25	A table showing baseline demographic and clinical characteristics for each group	13
Numbers analysed, outcomes and estimation	26	For each primary and secondary outcome, by group:<br>● the number of participants included in the analysis<br>● the number of participants with available data at the outcome time point<br>● result for each group, and the estimated effect size and its precision (such as 95% confidence interval)<br>● for binary outcomes, presentation of both absolute and relative effect size	Figure 1 and figure legend
Harms	27	All harms or unintended events in each group	9, Ref 11
Ancillary analyses	28	Any other analyses performed, including subgroup and sensitivity analyses, distinguishing pre-specified from post hoc	10 and Extended Data Figure 1 and 2 and supplementary tables S3 and S7
**Discussion**			
Interpretation	29	Interpretation consistent with results, balancing benefits and harms, and considering other relevant evidence	4-6

Limitations	30	Trial limitations, addressing sources of potential bias, imprecision, generalisability, and, if relevant, multiplicity of analyses	5-6

Citation: Hopewell S, Chan AW, Collins GS, Hróbjartsson A, Moher D, Schulz KF, et al. CONSORT 2025 Statement: updated guideline for reporting randomised trials. BMJ. 2025; 388:e081123.
© 2025 Hopewell et al. This is an Open Access article distributed under the terms of the Creative Commons Attribution License), which permits unrestricted use, distribution, and reproduction in any medium, provided the original work is properly cited.

*We strongly recommend reading this statement in conjunction with the CONSORT 2025 Explanation and Elaboration and/or the CONSORT 2025 Expanded Checklist for important clarifications on all the items. We also recommend reading relevant CONSORT extensions. See .

This supplement contains the following items:

1. Original protocol, final protocol, summary of changes.

2. Original statistical analysis plan, final statistical analysis plan, summary of changes.

*S*ynergy effect of the appetite hormone GLP-1 (*Li*raglu*T*ide) and *E*xercise on maintenance of weight loss and health after a low calorie diet – the *S-LiTE* randomized trial

The Universal Trial Number (UTN) is U1111-1173-3104

Sponsor
Associate professor, PhD Signe Torekov
Group Leader
Deputy Director of Education
University of Copenhagen
Faculty Of Health Sciences
Department of Biomedical Sciences
Novo Nordisk Foundation Center for Basic Metabolic Research
Blegdamsvej 3B
DK-2200 Copenhagen N
Denmark
TEL +45 35327509/ +45 22983827
torekov@sund.ku.dk

Principal Investigator
Professor, DMSci Sten Madsbad,
Department of Endocrinology,
Hvidovre University Hospital
Kettegård Allé 30, 2650 Hvidovre, Denmark
TEL +45 38622243
Sten.Madsbad@regionh.dk

Sub-investigators
Professor, DMSci Jens Juul Holst, Department of Biomedical Sciences, University of Copenhagen
Professor, DMSci Bente Stallknecht, Post doc
Mads Rosenkilde, Department of Biomedical Sciences, University of Copenhagen
MD, PhD student Julie Lundgren, Department of Biomedical Sciences, University of Copenhagen
Cand.scient, PhD student Charlotte Janus

Trial centres:
Department of Biomedical Sciences, University of Copenhagen, Blegdamsvej 3B, 2200 Copenhagen N, Danmark
Department of Endocrinology, Hvidovre University Hospital, University of Copenhagen, Kettegård Allé 30, 2650 Hvidovre, Denmark
TEL +45 38622243

Other hospital departments, technical departments and/or institutions involved in the trial:
Division of Cardiovascular Medicine, John Radcliffe Hospital, Oxford University, Headley Way Oxford, OX3 9DU, UK
Novo Nordisk A/S, Novo Allé, 2880 Bagsværd, Denmark
Cambridge Weight Plan Ltd, Clare House, Hunters Road, Corby, Northants, NN17 5JE

GCP

The GCP unit of the University Hospitals of Copenhagen,
Bispebjerg Hospital, Building 51, 3rd floor
Bispebjerg Bakke 23
2400 Copenhagen, Denmark
will ensure that the trial follow GCP guidelines.

Signatures and dates 9.6.16

Sten Madsbad, Professor
Overlæge, dr. med.
Endokrinologisk afdeling 541
Region Hovedstaden
Hvidovre Hospital
Kettegaard Alle 30, 2650 Hvidovre

**Signe Torekov**	**Sten Madsbad**
Sponsor	Principle investigator

Summary

Diabetes and obesity are global challenges to our healthcare system as the prevalence of individuals with overweight and/or type 2 diabetes (T2D) reaches more than 1 billion humans. We are approaching the abyss why the need for better and new interdisciplinary-based treatment strategies has never been greater. Diet-induced weight loss maintained with physical activity combined with incretin-based treatment may be such an example. Obese subjects often suffer from deteriorating metabolic health, such as impaired glucose tolerance, and furthermore recent studies report that sedentary lifestyle may shift the glucose intolerance of these subjects towards a full-blown T2D diagnosis. Alarmingly, diet-induced weight loss alone does not substantially improve long term metabolic health. The secretion of the gluco-regulatory hormone glucagon-like peptide-1 (GLP-1) is significantly attenuated in both obesity and pre-diabetic individuals and GLP-1R agonists are currently being used in the clinic for treating T2D and recently also obesity. Our aim is to prevent weight regain and potentially reverse deterioration of metabolic health in obese individuals glancing at a high risk of developing metabolic disease. We hypothesize that by reversing sedentary lifestyles in obese and potentially pre-diabetic and metabolic syndrome subjects with diet-induced weight loss maintained with habitual physical activity and additionally treating these individuals with a GLP-1R agonist their potential improvement in their weight, body composition and metabolic health may reach synergistic levels compared to either of these treatments alone. To investigate this, we will perform a randomized clinical trial with 4 intervention arms: Placebo, physical activity, daily subcutaneous injection with a GLP-1R agonist (liraglutide 3mg) and finally physical activity plus injection with a GLP-1R agonist (liraglutide 3mg). We believe that improved integration of interdisciplinary-based treatment therapies such as an initial diet-induced weight loss maintained with the combination of physical activity and GLP-1R agonist in individuals struggling with obesity may provide substantial improvement on their body composition and metabolic health.

Background and significance

Obesity is a common disorder, and in many western countries and the United States more than half of the populations are overweight or obese. The prevalence is increasing dramatically, and the number of obese individuals worldwide is now projected to reach as much as 1.1 billion by 2030 (1). As obesity is associated with a high rate of morbidity and mortality, and thereby accounts for a large part of the public health care expenses (2), there is a great interest in treatment of obesity. Usually most obese people experience several successful weight losses, however only few are able to maintain the weight loss over a longer period (ex. one year). Furthermore, obesity involves both metabolic and inflammatory disturbances and diet-induced weight loss alone does not substantially improve long term metabolic health (3; 4).

Glucagon-like-peptide-1 (GLP-1) is a hormone that is secreted from specific endocrine L-cells in the intestine upon meal intake. GLP-1 inhibits appetite and reduces food intake (5). Obese people have low endogenous GLP-1 response (6) and weight loss induces a marked increase in GLP-1 response (35). We have recently shown that treatment with GLP-1 analogues (liraglutide 1.2 mg) can facilitate long term weight loss maintenance (12 kg) with substantial improvement in metabolic health, in contrast to similar diet induced weight loss maintenance (3; 4; 7). Moreover, once daily injections of the GLP-1 analogue (liraglutide 3 mg) has been shown to facilitate substantial long-term weight loss (1 year) of approximately 8.0% in obese subjects (8).

On the other hand regular physical activity has also been put forward as effective lifestyle component for weight loss maintenance (9) and recent data show that maintainers of weight loss are more likely to perform regular exercise as opposed to subjects that regains weight loss (10)(36). In response to 3 months of daily endurance exercise we recently found no increase in appetite in overweight males (11) despite the exercise program induced significant weight loss and reduction of fat mass (4 kg) (12). Also, The SCALE maintenance trial showed that liraglutide (3 mg), with diet and exercise recommendations, maintained weight loss achieved by caloric restriction and induced further weight loss over 56 weeks (7). Indeed, long term intensive lifestyle intervention with a strong exercise component also results in clinical meaning full weight loss with the same magnitude (8.6%) for obese subjects as is observed with GLP-1 analogues (13). While exercise is known to improve the sensitivity to a variety of endocrine functions, e.g. insulin sensitivity (14), the combined and/or synergistic effects of liraglutide and exercise for weight loss maintenance and metabolic health are not known.

Physical exercise influences systemic and adipose tissue inflammatory pathways differently than diet-induced weight loss, and weight loss with physical activity has been suggested to induce a more healthy immuno-metabolism profile compared to diet-induced weight loss alone (15). An acute bout of physical activity has been shown to accentuate the hormonal meal-induced satiety response, e.g. increase in GLP-1(16). Interestingly, emerging evidence also places GLP-1 as a potential important immune metabolic modulator. GLP-1R agonists reduce endoplasmic reticulum stress and decrease inflammation-associated gene and protein expression in macrophages (17) as well as exerting anti-inflammatory actions in adipocytes (18). The GLP-1 analogue, liraglutide, exerted anti-inflammatory actions in endothelial cells (19). In humans, treatment with exenatide, a GLP-1R agonist, has shown potent anti-inflammatory effects (20). Whether these actions are due to GLP-1 receptor activation, or the weight lowering actions of GLP-1R agonists, remain uncertain. Notably, a small study of 10 obese subjects with T2D treated with liraglutide for 8 weeks indicated that GLP- 1 analogue therapy directly modulates innate immune-mediated inflammation (21), independent of weight loss, by significantly decreasing the novel marker of macrophage activation: the soluble form of haptoglobin–haemoglobin receptor CD163 (sCD163). In obesity, macrophages become pro-inflammatory, and removal of CD163 becomes up-regulated and is measurable as soluble CD163. Interestingly, sCD163 is strongly associated with insulin resistance independently of inflammatory markers such as TNF-α(22) and recently, sCD163 was found to be a strong predictor of the development of type 2 diabetes in a large Danish cohort as well as a novel marker for the coronary atherosclerotic burden (23). Interestingly, the combination of diet-induced weight loss and exercise decreased sCD163 levels (37). Thus, sCD163 may reflect the link between low-grade inflammation and metabolic abnormalities in the obese state.

Although GLP-1 analogues (like liraglutide) have a global anti-inflammatory potential seen even as early as 8 weeks after initiation of treatment(20; 21), it is unclear whether their potential effect on the vascular wall is mediated by the GLP-1 receptor (GLP-1R), changes in insulin levels or the weight loss induced by these agents. Post-GLP-1R signalling has multiple immuno-metabolic effects (increased sCD163, activation of PI3K/Akt signalling and suppression of MAPK in vascular cells (24), which is also observed with exercise (25; 26)) implying that GLP-1 analogues and exercise may supress vascular oxidative stress, reducing cardiovascular complications of obesity. In addition, GLP-1 analogues and exercise may shift the secretory profile of human adipose tissue towards an anti-inflammatory state exerting additional protective effects on the cardiovascular system.

Thus, exercise and GLP-1 analogues both seem to facilitate weight loss maintenance and reduce systemic inflammation and may thus be used synergistically as obesity treatment to improve both inflammatory and metabolic health.

Research question

Study Hypothesis

We hypothesize that there is a synergy effect on maintenance of weight loss and beneficial health outcomes between treatment with the appetite hormone GLP-1 (liraglutide) and an exercise program in obese individuals.

Objective

To investigate the maintenance of weight loss and health outcomes (glucose and insulin for HOMA-IR and Matsuda index, HbA1c, lipids (i.e. cholesterol, HDL, LDL, triglycerides FFA), determination of glucose-tolerance status, biomarkers of inflammation (sCD163, hsCRP, IL6, IL1, IL1Rap, TNF-α)) over 1 year with GLP-1 treatment (liraglutide), exercise treatment and the combination in obese persons that have lost at least 5 % of their body weight by low calorie diet during 8 weeks.

Research design and Methods

Study population

Recruitment of 180 obese persons (ideally with equal number of men and women) in local newspapers, via electronic media and from Department of Endocrinology, Hvidovre University Hospital as well as Department of Biomedical Sciences, University of Copenhagen.

<u>*Inclusion criteria:*</u>

- BMI > 32 and < 40 (kg/m^2)
- Age >18 and <65 year
- Safe contraceptive method.

<u>*Exclusion criteria:*</u>

- Patients diagnosed with *known* serious chronic illness including type 1 or 2 diabetes (or a randomly measured fasting plasma glucose > 7 mmol/l)
- Angina pectoris, coronary heart disease, congestive heart failure (NYHA III-IV)
- Severe renal impairment (creatinine clearance (GFR) <30 mL/min)
- Severe hepatic impairment
- Inflammatory bowel disease
- Diabetic gastroparesis
- Cancer
- Chronic obstructive lung disease
- Psychiatric disease, a history of major depressive or other severe psychiatric disorders
- The use of medications that cause clinically significant weight gain or loss
- Previous bariatric surgery
- A history of idiopathic acute pancreatitis
- A family or personal history of multiple endocrine neoplasia type 2 or familial medullary thyroid carcinoma

- Osteoarthritis which is judged to be too severe to manage the exercise programme. As intended per study design the intervention will include a 5% weight loss prior to randomization, thus it is expected that possible participants with mild form of osteoarthritis will be able to manage exercise prescriptions.
- Pregnancy, expecting pregnancy or breast feeding. If a study participant is in doubt whether she could be pregnant, a urine pregnancy test is performed. Females of childbearing potential who are not using adequate contraceptive methods (as required by local law or practice). Adequate contraception must be used throughout the study period and at least 65 hours after discontinuation of trial medication (65 hours corresponds to 5 times the half-life of Saxenda). Allergy to any of the ingredients/excipients.
- Allergy to any of the ingredients/excipients of the study medication: liraglutide, disodium phosphate dihydrate, propylene glycol, phenol, hydrochloric acid, sodium hydroxide

Withdrawal criteria: The subject may withdraw at will at any time. If a subject withdraws after week 20 we will offer to perform the investigations planned at week 52 at the time of withdrawal. The subject may be withdrawn from the trial at the discretion of the Investigator due to a safety concern or if judged non-compliant with trial procedures.

A subject must be withdrawn if the following applies: Pregnancy or intention to become pregnant.

Subject replacement: Withdrawn subjects will not be replaced. However, re-screening is allowed within the recruitment period, at the Investigator's discretion.

Reminders: To minimize loss to follow-up, reminders (in the form of text messages or letters) will be sent to the participants prior to the examination dates.

Follow-up procedure

In case of drop-out, the participant will be asked to complete a full set of examinations at the time of drop out.

Study design

Randomized controlled parallel groups clinical trial. The trial is double blinded in regards to study medication. Exercise cannot be blinded for the participants, however, study personnel performing the exercise tests are blinded in regards to randomization to exercise program. Study design and work flow is outlined in figure 1 and 2.

This trial will recruit 180 obese individuals. The weight loss is obtained during 8 weeks with a very low calorie diet (VLCD) (800 kcal/day), Cambridge diet, which is a well-known initial treatment of obesity (3), with the objective to lose at least 5 % body weight (expected to be achieved in at least 160 subjects, and less than 15 will drop out during this phase based on our prior studies(3; 4)). Compliance with the dietary maintenance program will be assessed each week at weight consults with a dietician/scientific personnel during the weight loss period. After 8 weeks of weight loss the participants are randomized to receive a) Liraglutide 3mg/day sc or b) placebo sc or c) Exercise + placebo sc or d) Exercise + Liraglutide 3mg/d sc for 52 weeks. Weekly/monthly consultations, weighing and collection of fasting blood samples will be continued in all 4 groups during the study period.

Liraglutide or placebo intervention: Daily injections (3mg) with diet/weight consultations starting at dose of 0.6 mg injections with 0.6 mg increments weekly until 3.0 mg is achieved. For subjects who do not tolerate the fast weekly up-titration of 0.6 mg study drug until the 3mg, the titration procedure can be prolonged with up to three weeks for each up-titration. Subjects who do not tolerate the 3mg dose may in special

circumstances stay at 2.4 mg, however the overall aim is to reach 3 mg for all study subjects. The dosage and up-titration follow the recommendations from the summary of product characteristics.
Exercise intervention: Health authorities (WHO) recommend exercise 30 min/day 5 days/week. To make our study possible, we aim for 4 exercise days per week. Exercise prescription will be performed under strict control of the scientific personnel. There will be aerobic exercise and will include 5 sessions per week after the ramp-in period. 2 sessions per week will be performed under supervision of the staff and 2 sessions will be performed individually but monitored by the staff. *Week 1-2*: 1 supervised session/week, *week 3-4*: 1 supervised and 1 individual session/week, *week 5-6*: 2 supervised and 1 individual sessions/week, *week 7-52*: 2 supervised and 2 individual sessions/week. If this up regulation of exercise is not possible (for example due to side effects of study medication), up regulation will proceed more slowly. Supervised sessions include structured exercise with an exercise intensity > 60% VO_2peak, individual exercise includes general physical activity such as brisk walking or cycling to work. Participants will use heart rate monitors during session. Furthermore participants will be instructed to perform mostly non-weight bearing activities in the start of the intervention, such as cycling and rowing but brisk walking on a treadmill with inclination may also be performed.
Liraglutide+exercise: Combination of the two groups.
The trial ends at week 52 where Saxenda/placebo and exercise treatment is discontinued. Saxenda/placebo does not need to be phased out. Elimination half-life of Saxenda is approximately 13 hours.

Randomization
The subjects are randomized to 1 of the 4 treatment arms in a 1:1:1:1 ratio, according to the subject randomization list (SRL) provided by Novo Nordisk. A qualified un-blinded study nurse (not otherwise associated with the trial) will allocate study participants according to the SRL. Randomization will be stratified by gender and age. Two lists are made; one for participants over 40 years and one for participants under 40 years of age. Males will be gives the lowest available number from the appropriate list (from the top of the list) and females will be gives the highest available number from the appropriate list (from the bottom of the list).
Novo Nordisk also provides a total dispensing unit number list (TDL). The un-blinded study nurse will allocate trial medication using the TDL by matching a six digit Dispensing Unit Number (DUN) to the correct treatment. Each box will be labeled with a unique DUN. The DUN alone is not un-blinding. Thus, the dispensing of trial medication to subjects can be carried out by blinded trial staff, by selecting the DUN(s) provided by the un-blinded study nurse. The SRL and TDL are to be kept with restricted access only to the designated un-blinded study nurse.

Un-blinding
The subject randomization list (SRL) and the total dispensing unit number list (TDL) is stored on site at Hvidovre Hospital. Subject number of the trial participant is matched with the SRL and TDL, which reveals if trial medication is liraglutide or placebo. Preferably, the un-blinded study nurse will perform any un-blinding of study participants. However, if needed, all trial staff (sponsor, investigator and sub-investigators) can get access to the SRL and TDL and perform the un-blinding procedure.

Examinations
V0/screening (before weight loss)

- Measurement of weight, height, waist and hip circumference, blood pressure and pulse
- Meal tests (with liquid meal) are performed and blood samples will be obtained before, during and 3 hours after meal intake for plasma/serum samples (glucose, insulin, C-peptid, leptin, soluble leptin receptor, GLP-1, PYY, ghrelin, glucagon, adiponektin, free fatty acids, sCD163, hsCRP, IL-1, IL-1Rap IL-6, TNF-α, SAA1, SAA2, ORM1, ORM2, malonyldialdehyde, F2-Isoprostanes, CTX and P1NP, FGF21) and peripheral blood mononuclear cells (PBMNCs) isolation.
  Here amongst, a set of standard samples are collected and analysed on the same day. These include haemoglobin, free calcium, creatinine and GFR, potassium, sodium, CRP, ALAT, amylase, alkaline phosphatase, vitamin D, HbA1c, PTH, TSH, total cholesterol, HDL, LDL, VLDL and triglycerides. Among other, we use these for monitoring the safety of the study participants.
- Endothelial function of the brachial artery will be evaluated using flow mediated dilatation (FMD) (27) and ECG's are obtained.
- Body, fat and bone composition will be measured with DEXA and MRI scans.
- Subcutaneous adipose tissue biopsies (abdominal) are taken under local anaesthesia.
- Faecal, urine, saliva and semen samples are collected.
- Food preferences determined by standardized pictures
- Subjective appetite sensations using visual analogue scales (VAS)
- Questionnaires will be given to the subjects to determine 1) self-rated quality of life (SF-36), 2) eating habits (three-factor eating questionnaire), 3) IPAQ, 4) food preferences.
- Physiological tests: we will perform a peak oxygen test, strength test and walking distance test.

We aim at completing all tests on one day. If this is not possible, the remaining tests are performed at an adjacent test day.

V1/baseline (after weight loss phase; randomization): Same tests as described for V0.

V2/mid (26 weeks after randomization)

- Blood samples
- Subcutaneous adipose tissue biopsies (abdominal) are taken under local anaesthesia.

V3/end (52 weeks after randomization): Same tests as described for V0.

Furthermore, fasting blood samples are drawn at week 4, week 13 and week 39.

Criteria for when tests can be performed:

- The participant has to be fasting from the previous evening (from 22:00), including food, liquids and medication (except study medication)
- Study medication should be taken on the morning of the tests
- Training should not be performed the day before tests

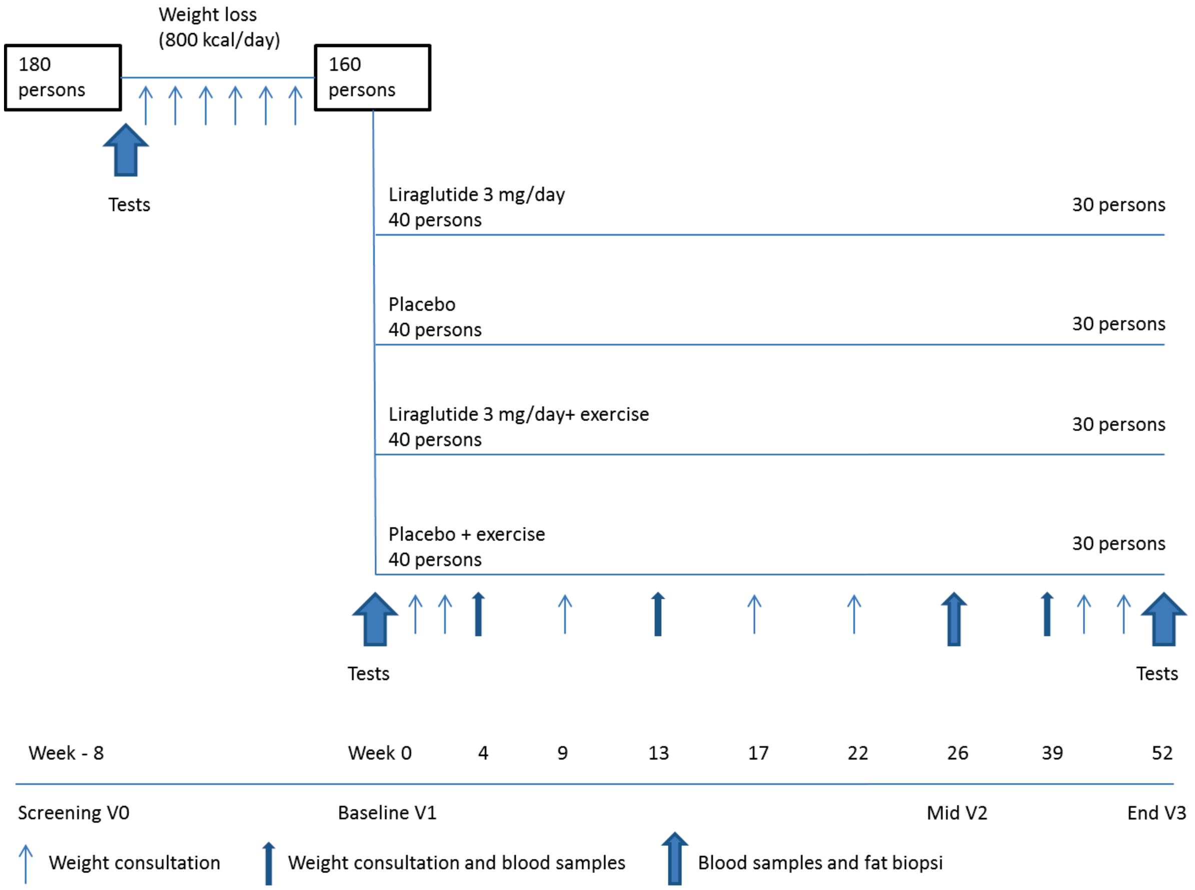

Figure 1 Study design; *Tests* refer to all the tests/examinations described in the *Examination* section for V0.

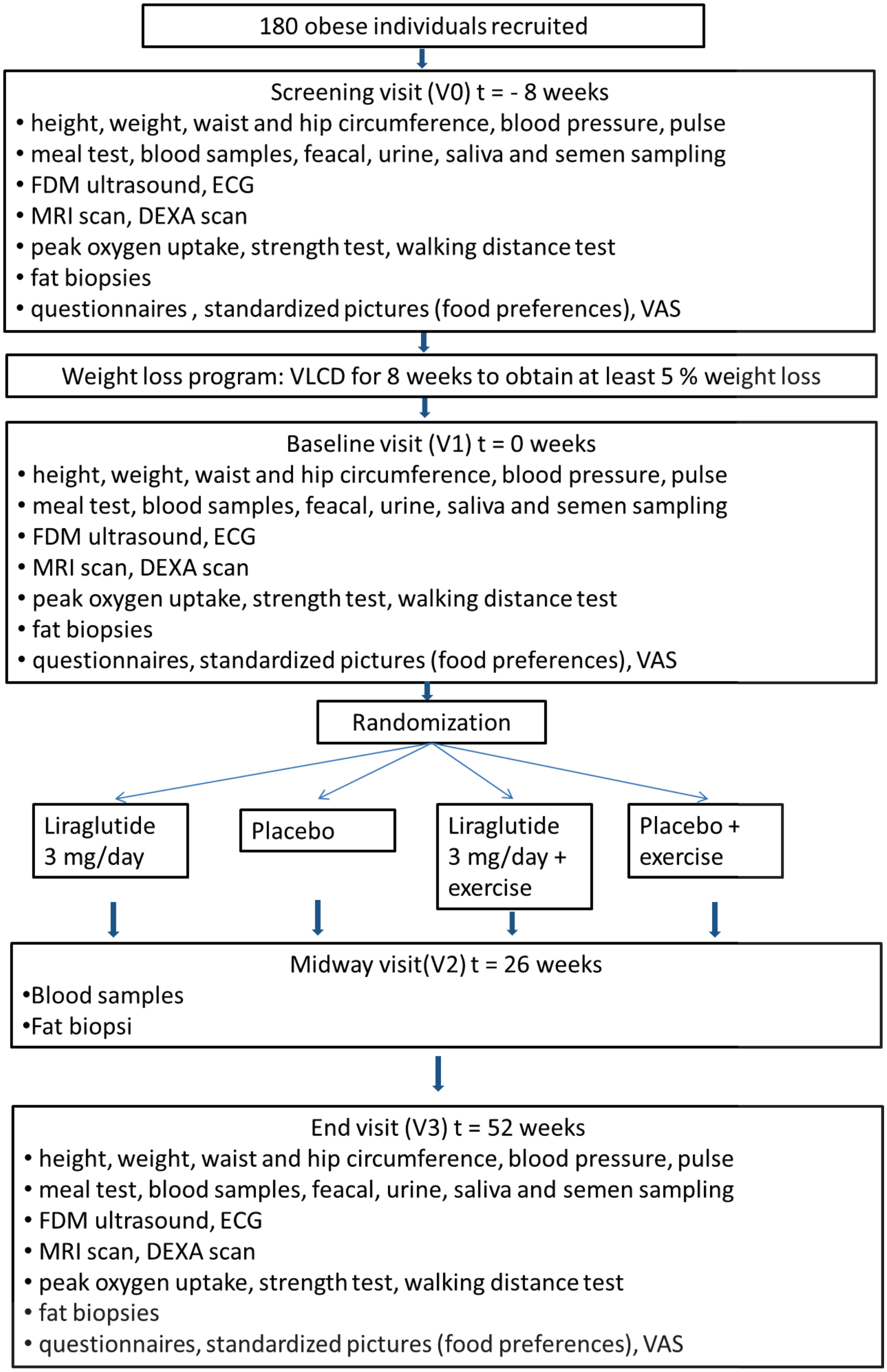

Figure 2 Work flow (FMD: flow mediated dilatation, VLCD: very low calorie diet (800 kcal/day))

Endpoints

Primary endpoints (change from V1 to V3)
Body weight change (kg)
Weight will be measured to the nearest 0.1 kg. The same set of scales should ideally be used throughout the trial. Weight should be measured in a fasting state without shoes and wearing light clothes.
Secondary endpoints (change from V1 to V3)
1) Metabolic health (glucose tolerance and lipid status, waist circumference, blood pressure, Composite Metabolic syndrome Z-score) (2) Meal related appetite response (3) peak oxygen uptake and determination of daily physical activity (4) Systemic markers of immune-metabolism and oxidation (5) Endothelial function (6) Immuno-metabolic changes in the subcutaneous adipose tissue (7) Bone health (8) Lipid content of liver (9) gene expression profile of circulating inflammatory cells (10) changes in body composition (11) Food preferences and subjective appetite sensation
Tertiary endpoints (change from V1 to V3)
(1) Faecal bacterial composition, (2) Plasma metabolomics and proteomics, (3) epigenetics of spermatozoa, (4) activation of brown fat and FGF21

Specific aims and endpoints
<u>Primary objective</u>: To explore the long-term synergistic effects of liraglutide and exercise program on the maintenance of weight loss and body composition after very a low calorie restriction diet: This will be explored by weekly/monthly weighing of subjects during their weight.
<u>Secondary objectives 1-4</u>: To explore the long-term synergistic effects of liraglutide and exercise program on and metabolic health, appetite regulation, oxygen uptake and systemic markers of immune-metabolism and oxidation: We will measure circulating biomarkers of metabolic regulation to evaluate metabolic health (glucose and insulin for HOMA-IR and Matsuda index, HbA1c, lipids i.e. cholesterol, HDL, LDL, triglycerides FFA and determination of glucose-tolerance status). We will measure hormonal appetite regulation during meal tests and fasting (GLP-1, Peptide YY, Glucagon, leptin, Ghrelin, adiponectin) using our standard methodologies (3). Peak oxygen uptake and determination of daily physical activity will be obtained. Cardiorespiratory fitness (VO_2 peak) is assessed using an electronically braked bicycle and indirect calorimetry at V0, V1 and V3: After warm-up workload is increased with 25 W increments every minute until attainment of VO_2peak, based on the following criteria: Levelling off in oxygen uptake and RER-value above 1.15 or age-predicted heart rate max (220 minus age).
In plasma samples from V0, V1, V2, V3 we will measure various biomarkers of inflammation (sCD163, hsCRP, IL-1, IL-1Rap IL-6, TNF-α, SAA1, SAA2, ORM1, ORM2) and oxidation (malonyldialdehyde, F2-Isoprostanes, etc.) using methodologies well established in the lab in Oxford (28).
<u>Secondary objective 5</u>: To explore the long-term effects of liraglutide and exercise program on vascular endothelial function: We will use Flow-mediated vasodilation (FMD) to study changes in endothelial function (27) as well as blood pressure and electrocardiogram (ECG) measurements at V0, V1 and V3. FMD is an endothelium-dependent function, explored by a non-invasive technique, in the brachial artery. The brachial arteries are scanned with high resolution ultrasound imaging at rest and during hyperaemia. Hyperaemia can be induced by inflation and deflation of a sphygmomanometer cuff around the forearm, distal to the site scanned later with ultrasound. Peripheral arteries, including the brachial artery, respond to physical and chemical stimuli by adjusting vascular tone and regulating blood flow.

Increased blood flow in peripheral arteries is leading to increased shear stress stimuli, increased nitric oxide production, and vasodilation. The vasodilatory response of the brachial artery to increased shear stress is called flow mediated dilation (FMD), and reflects the ability of vascular endothelium to produce NO. Endothelial function, defined as FDM is defined as the percentage increase in vessel diameter from baseline conditions to maximum vessel diameter, during hyperaemia. FMD is estimated as the percentage change of the brachial artery diameter from rest, to the vessel diameter 60 seconds after the cuff is released. After a 12 hour fasting period, the subject is positioned in a supine position, in a quiet room, with constant temperature. The arm is placed in a comfortable position for assessing the brachial artery, and must remain under constant conditions for at least 10 minutes.
All vasoactive drugs must be discontinued at least 12 hours before the study, and the patient must not ingest substances that might affect FMD such as caffeine, high-fat food and vitamin C nor smoke, for at least six hours before the study. The FMD includes administration of 400 micrograms of nitro-glycerine. This method has the advantage of being non-invasive and safe with no risk concerns. From the previous studies (27) with 30 study participants in each group we expect to be able to detect a 10% difference between groups.
<u>Secondary objective 6</u>*: *To explore the long-term effects of liraglutide and exercise program on immunometabolic profile of the human subcutaneous adipose tissue: In adipose tissue biopsies obtained at V0, V1 and V3 we will: a) determine the changes in the gene expression of GLP-1 receptor, and target-genes downstream the GLP-1 receptor that include pro-inflammatory (e.g. IL-6, IL1b, MCP-1, resistin, leptin, chemerin, etc.) and anti-inflammatory (e.g. adiponectin) adipocytokines that could have an endocrine/paracrine effect on cardiovascular function. The changes in the expression of adipocyte differentiation markers such as FAB4, CABPA and PPAR-γ as well as markers of macrophages infiltration (CD163, CD68) and M1/M2 phenotype of them such as CD40, CCR7, CD207 etc. (all by qRTPCR) will also help us understand the potential phenotypic changes in adipocyte differentiation status and inflammatory infiltration after treatment. The sc adipose tissue (1 g) will be obtained by needle aspiration under local anaesthesia using 5–10 ml 0.5% lidocaine. It has previously been demonstrated that this procedure does not influence adipocyte metabolism. This is a standard procedure, safe and with local anaesthesia non-painful.
<u>*Secondary* objectives 7-8</u>: *To explore the long-term effects of liraglutide and exercise program on reversal of fatty liver and weight loss induced bone loss*
Conventional Magnetic resonance imaging (MRI) is used to assess effects on fat deposits in liver. MRI exploits the difference of the resonance frequencies between water and fat proton signals. By acquiring the images at echo times at which water and fat signals are approximately in-phase (W+F) and opposed-phase (W-F), volumetric liver fat detection is possible based on the relative signal loss on opposed-phase (also known as "out-of-phase") images (no contrast testing is necessary). MRI uses a powerful magnetic field, radio waves, rapidly changing magnetic fields, and a computer to create images. An MRI examination causes no pain, and the magnetic fields produce no known tissue damage of any kind, and is thus safe. Site specific bone-measurements and bone markers (CTX and P1NP) as well as DEXA scans to assess bone-health (4).
<u>Secondary objective 9</u>*: *Determine the effects of long-term treatment with liraglutide and an exercise program on gene expression profile of circulating inflammatory cells (PBMNCs) obtained at V0, V1, and V3.
<u>Secondary objective 10:</u> Determine the effect of liraglutide and/or exercise on body composition
Study participants are scanned by DEXA at V0, V1 and V3. These scans can be used to determine fat mass, lean mass and fat percentage.
<u>Secondary objective 11:</u> Determine the effect of liraglutide and/or exercise on food preferences and appetite sensation

Food preferences, in particular liking and wanting of food items, are assessed by a picture display test where standardized pictures of the following 20 food items are shown: pork rib roast, French fries, nuggets, salty crackers, cheese, smoked fillet (cold cuts), omelet, carrots, crisp bread, turkey strips, Danish pastries, vanilla ice cream, milk chocolate, pound cake, cookies, cut fruits, skyr (yoghurt) with berries, sweet licorice, cocoa meringues and wine gum. These 20 food items can be organized into combined and separate food categories. Pictures are displayed in a randomized order and the participants are instructed to rate each individual food item and to choose the three food items they prefer the most in a prioritized order (43).
Subjective appetite sensations will be obtained during a fixed standardized meal using visual analogue scales (VAS) to record hunger, satiety, fullness, prospective food consumption, desire to eat something fatty, salty, sweet or savory, and palatability of the meals (44).
Tertiary objectives: ***Determine the effects of long-term treatment with liraglutide and an exercise program on*** faecal bacterial composition, plasma metabolomics, proteomics profile, brown fat and epigenetics of the spermatozoa at V0, V1 and V3. Epidemiological data support that paternal nutritional status can directly affect the health of the offspring (38;39), suggesting that an environmentally acquired phenomenon of epigenetic inheritance is passed on by the gametes. Recently, Barres showed that spermatozoa of obese men carry altered epigenetic signals specifically located at regions controlling the development of the central nervous system and the control of appetite. Moreover, they showed that weight loss, induced by bariatric surgery, remodels the epigenetic profile of spermatozoa, suggesting that pre-conceptional behaviour targeting weight loss may be relevant to the metabolic health of the next generation (40). To test the ability of obese subjects to activate brown fat, thermo-scanning (cold exposure by blanket roll and measuring the skin temperature in the supraventricular neck region by thermo-scanning camera (41)) will be set up and performed before and after treatment. Furthermore, levels of FGF21 (a metabolic growth factor released from the liver in response to exercise) are measured. Brown fat recruitment has been shown to be stimulated by FGF21 (42).

Data

Data management

The subject and the biological material obtained from the subject will be identified by subject number and trial identification number. Appropriate measures such as encryption or deletion will be enforced to protect the identity of human subjects in all presentations and publications as required by national requirements.
Laboratory data will be transferred electronically from the laboratory performing clinical analyses and will be archived in secured hard drives with backup options. The electronic data will be considered source data. In cases where laboratory data is transferred via non-secure electronic networks, data will be encrypted during transfer. Data is saved for 20 years.

Source data

Source documents will be kept in the Investigators file.
Source data registered directly in the CRF include blood pressure, weight, height, hip and waist measurements, anamnesis and adverse events.

Evaluability of subjects for analysis

Withdrawn subjects will not be replaced. However, re-screening is allowed within the recruitment period, at the Investigator’s discretion.

Two analysis sets are defined

Per Protocol (PP) analysis set: All exposed subjects who complete the 52-week randomized treatment period without significantly violating the inclusion/exclusion criteria or other aspects of the protocol considered to potentially affect the efficacy results.

Intent to treat (ITT) analysis set: All randomized subjects exposed to at least one dose of trial product and completed the visit at week 4.

Safety analysis will be performed on the ITT population.

The decision to exclude any subject or observation from the statistical analysis is the responsibility of the sponsor and will be agreed on before breaking the blind. The subjects or observations to be excluded, and the reasons for their exclusion must be documented and signed by those responsible prior to database release. The documentation must be stored together with the remaining trial documentation.

Analysis will be based on the ITT analysis set, unless stated otherwise. Analysis of the efficacy variables will also be performed on the PP analysis set as supportive evidence.

Data analysis plan

All variables will be tested for normality with the Shapiro-Wilk Test and/or the Kolmogorov-Smirnov Test and log-transformed if non-normally distributed to achieve normality. Then parametric tests will be used throughout the analyses, if normality is achieved. If normality cannot be achieved through log transformation other transformations will be tried, if non-successful non-parametric analyses will be applied.

Delta changes in variables (from week 0 - week 52) will be analysed using a general linear model (ANCOVA) with age and sex and baseline weight as covariates and treatment as an effect (SPSS statistics version 22, IBM Corporation, Armonk, New York, United States). The effect of treatment on serial measurements (before vs after treatment in the four groups) will be assessed by ANOVA for repeated measures with time x treatment interaction terms and age and sex and baseline weight as covariates. We will use drug treatment (lira, placebo), exercise (yes, no) and their interaction to investigate the interaction between drug and exercise.

Statistical methods

All two-sided tests are at the 5% significance level.

Primary endpoint

The change from baseline (randomization) in weight (kg) after 52 weeks of treatment will be analysed using an analysis of covariance (ANCOVA) model with treatment group as explanatory variable and baseline weight, sex and gender as covariates. Missing values will be replaced using last observation carried forward. The objective of the analysis is to determine whether the effect on weight (as measured
by kg) and body composition (lean/fat body mass ratio) of 3mg liraglutide in combination with exercise program is superior to 1) placebo and if so to 2) to either treatments alone.

Secondary endpoints

The secondary efficacy endpoints are: metabolic health measured by the metabolic syndrome z-score. Analysis of covariance models as described above will be used to test for superiority. The change from baseline (randomization) after 52 weeks of treatment will be analysed using an analysis of covariance

(ANCOVA) model with treatment group as explanatory variable and baseline weight, sex and gender as covariates.

Other endpoints

Other endpoints are described above. The change from baseline (randomization) after 52 weeks of treatment will be analysed using an analysis of covariance (ANCOVA) model with treatment group as explanatory variable and baseline weight, sex and gender as covariates.

Safety endpoints

Endpoints related to safety are: AEs, laboratory tests (haematology and biochemistry)

AEs: Frequencies of subjects experiencing treatment emergent AEs (TEAE) and frequency of the events will be tabulated by system organ class and preferred term. Listings of TEAEs, non-TEAEs, SAEs, and AEs leading to withdrawal will be provided.

A TEAE is defined as an event that

- Occurs after randomization and increases in severity during the treatment period
- Occurs between first and last day of treatment + seven days

Clinical laboratory tests: Haematology and biochemistry will be summarized descriptively together with change from baseline. Abnormal values will be listed.

Sample size calculation

Based on our previous studies (3; 4) only 10 % did not obtain a weight loss of more than 10% during the initial weight loss phase, thus we expect 160 out of 180 to obtain at least a 5% weight loss. We are planning a study with 30 experimental subjects and 30 control subjects. In our previous study (3; 4) the response within each subject group was normally distributed with standard deviation 5.5. If the true difference in the experimental and control means is 4kg, we will be able to reject the null hypothesis that the population means of the experimental and control groups are equal with probability (power) 0.8. The Type I error probability associated with this test of this null hypothesis is 0.05. If we unexpectedly experience higher withdrawal rates than stated more study subject will be recruited to obtain sufficient power. Retention modalities: by frequent weight and diet consultations with the medical doctor/dietician we have previously experienced that this motivated study participants to fulfil the study period.

Study time line

August 2016 – August 2021

Study medication

The active treatment will be subcutaneous injections of liraglutide 3mg (Saxenda) or placebo, that will be given once daily for 52 weeks after weight loss.

Treatment of subjects

Subjects will attend screening visit to assess their eligibility. If found eligible, subjects should return at visit 0. When the weight loss program has been completed they will be randomized to one of the four treatment arms.

The randomized treatment period lasts 52 weeks, including at least 5 weeks of dose escalation for the liraglutide/placebo/exercise treatment.

Labels and packaging

Novo Nordisk A/S will handle the labelling and packaging of the study medication. The text of the labels will be according to Eudralex Volume 4, Annex 13.

Accounting of study medication

To account for study medication schemas are used to 1) register reception of medication on site from Novo Nordisk (reception date, number of packages, batch number, expiration date, signatures) and 2) register the medication handed out to study participants (ID number of participant, date, batch number, expiration date, number of pens, number of pens returned, signatures).
For surveillance of compliance in terms of using the study medication, we will register number of returned pens. This will be set in relation to the amount of study medication used in case the medicine is used as prescribed.

Dose escalation and drug administration

Liraglutide will be available at a concentration of 6.0 mg/mL and supplied in 3 mL pen-injectors.
Dosing with the liraglutide pen-injector is controlled by turning the dose selector until the dose indicator lines up with the relevant dose. Liraglutide is administered once daily by subcutaneous injections with the pen-injector, either in the abdomen, thigh or upper arm. Injections can be done at any time of the day and irrespective of meals. It is recommended that the time of administration is repeated between consecutive days throughout the trial. Subjects will be instructed to perform an air shot before the first use of a new pen-injector. For further information, please see the handling instructions for the liraglutide pen-injector. These handling instructions will be provided in Danish language together with the trial products. The Investigator must instruct subjects how to inject liraglutide, and must ensure that the subjects are familiar with the instructions. Subjects will be instructed to escalate the liraglutide/placebo dose to 3 mg/day over a five week period, following an initiation dose of 0.6 mg/day and weekly dose escalation steps of 0.6 mg/day.

Storage of trial products

The liraglutide pen-injectors must be stored in a refrigerator at a temperature between + 2°C and + 8°C. Freezing must be avoided and the drug must not be used if it has been frozen. Liraglutide pen injectors must be protected from all sources of light, and the pen cap should be kept on when the pen is not in use. The in-use time for liraglutide is 1 month at temperatures below 30°C.
Trial medication will be stored in a locked room, separately of other medicines.

Concomitant illnesses and medication

Concomitant illness: any illness that is present at the start of the trial (i.e. at the first visit).
Concomitant medication: any medication other than the trial products that is taken during the trial, including the screening and run-in periods.
Details of all concomitant illnesses and medication must be recorded at trial entry (i.e. at the first visit). Any changes in concomitant medication must be recorded at each visit. If the change influences the subject's eligibility to continue in the trial, then the sponsor must be informed.

The information collected for each concomitant medication includes, as a minimum, start date, stop date or continuing and indication.

Safety (see appendix: SAFETY REPORTING REQUIREMENTS FOR INVESTIGATOR INITIATED STUDIES (IIS))

Definitions

Adverse Event (AE)	Any untoward medical occurrence in a participant to whom a medicinal product has been administered, including occurrences which are not necessarily caused by or related to that product. An Adverse Event (AE) can therefore be any unfavourable and unintended sign (including an abnormal laboratory finding), symptom, or disease temporally associated with the use of a product, whether or not considered related to the product.
Adverse Reaction (AR)	An untoward and unintended response in a participant to an investigational medicinal product which is related to any dose administered to that participant.<br>The phrase "response to an investigational medicinal product" means that a causal relationship between a trial medication and an AE is at least a reasonable possibility, i.e. the relationship cannot be ruled out.<br>All cases judged by either the reporting medically qualified professional or the Sponsor as having a reasonable suspected causal relationship to the trial medication qualify as adverse reactions.
Serious Adverse Event (SAE)	A serious adverse event is any untoward medical occurrence that:<br>• results in death<br>• is life-threatening<br>• requires inpatient hospitalization or prolongation of existing hospitalization<br>• results in persistent or significant disability/incapacity<br>• consists of a congenital anomaly or birth defect.<br>Other 'important medical events' may also be considered serious if they jeopardize the participant or require an intervention to prevent one of the above consequences.<br>NOTE: The term "life-threatening" in the definition of "serious" refers to an event in which the participant was at risk of death at the time of the event; it does not refer to an event which hypothetically might have caused death if it were more severe.<br>Suspicion of transmission of infectious agents must always be considered an SAE.
Serious Adverse Reaction (SAR)	An adverse event that is both serious and, in the opinion of the reporting Investigator, believed with reasonable probability to be due to one of the trial treatments, based on the information provided.

Suspected Unexpected Serious Adverse Reaction (SUSAR)	A serious adverse reaction, the nature and severity of which is not consistent with the information about the medicinal product in question set out:<br>• in the case of a product with a marketing authorization, in the summary of product characteristics (SmPC) for that product

NB: to avoid confusion or misunderstanding of the difference between the terms "serious" and "severe", the following note of clarification is provided: "Severe" is often used to describe intensity of a specific event, which may be of relatively minor medical significance. "Seriousness" is the regulatory definition supplied above.

Reference Document

The reference document for reporting adverse events is the Summary of Product Characteristics (SmPC) for Saxenda (European Medical Agency, published 16/04/2015). In particular section 4.8 "Undesirable effects".

Pregnancy

If pregnancy unexpectedly occurs the participant is immediately excluded from the trial. Any pregnancy occurring during the clinical trial and the outcome of the pregnancy should be recorded and followed up for congenital abnormality or birth defect, at which point it would fall within the definition of "serious". All pregnancies in trial subjects occurring during use of liraglutide will be recorded immediately to NN (Novo Nordisk).

Causality

The relationship of each adverse event to the trial medication must be determined by a medically qualified individual according to the following definitions:
Probable - Good reason and sufficient documentation to assume a causal relationship. Possible - A causal relationship is conceivable and cannot be dismissed. Unlikely - The event is most likely related to aetiology other than the trial product.
The reference document in the protocol which is agreed upon with NN, for evaluation of expectedness will be specified.

Procedures for recording Adverse Events

All adverse events will be actively collected from the first study-related activity (from the signing of the informed consent) and in all following contacts with the study-subject throughout the project. This includes events from the first trial related activity after the subject has signed the informed consent, and until the post treatment follow-up period, as defined in the protocol. Trial-related activities are any procedures (i.e. laboratory tests, X-rays, ECGs, fasting periods etc.) that would not have been performed during normal management of the subject.
As a minimum the following details will be reported -Study name -Patient identification (e.g. subject number, initials, sex, age) -Event (Preferably diagnosis) -Trial drug -Reporter -Causality -Outcome These details to be reported to the local NN affiliate safety department.
All AEs occurring during the trial that are observed by the Investigator or reported by the participant will be recorded on the CRF, whether or not attributed to trial medication.
The following information will be recorded: description, date of onset and end date, severity, assessment of relatedness to trial medication, other suspect drug or device and action taken. Follow-up information should be provided as necessary.
The severity of events will be assessed on the following scale: 1 = mild, 2 = moderate, 3 = severe.

AEs considered related to the trial medication as judged by a medically qualified investigator or the Sponsor will be followed either until resolution, or the event is considered stable.

Reporting of adverse events/reactions

SUSARs

We do not expect any SUSARs. The trial medication is approved for treatment of obesity and used in this trial as prescribed in the Summary of Product Characteristics. The study medication has been thoroughly tested in several clinical trials.

In case of any deadly or life-threatening SUSAR, Sponsor will immediately (and no later than 7 days after becoming aware) notify the Competent Authorities (Lægemiddelstyrelsen) and the Ethics Committee. No later than 8 days after reporting of a SUSAR, Sponsor will notify the Competent Authorities and the Ethics Committee of all relevant information about Sponsor and Investigator's follow-up of the SUSAR. All other SUSARs will be reported to the Competent Authorities and the Ethics Committee no later than 15 days after Sponsor becoming aware of this.

SARs

Investigator will immediately report all SARs to sponsor. The notification will be followed by a detailed written report. Once yearly, Sponsor will prepare a report regarding SARs occurring in the trial as well as describe the safety of the study participants in regards to continuation of the trial. This will be sent to the Competent Authorities and the Ethics Committee. Reporting will follow the EudraLex - Volume 10 Clinical trial guidelines (Detailed guidance on the collection, verification and presentation of adverse event/reaction reports arising from clinical trials on medicinal products for human use (CT 3)).

SAEs

Investigator will immediately report all SAEs to sponsor. The notification will be followed by a detailed written report. If Sponsor upgrades a SAE to a SAR, procedure is as described above.

SAEs, AEs, ARs

Will be noted in the CRF. These will be recorded in the End of Trial Form to the Competent Authorities and in the report to the Ethics Committee (if requested) no later than 90 days after trial completion.

To NN:

As a minimum requirement, the sponsor should copy NN when expediting SARs or SUSARs to Competent Authorities and should report all SARs related to NN Product to NN

As a minimum, the investigator should copy NN (Novo Nordisk) when expediting SARs or SUSARs to health authorities and should report all SARs related to NN product to the local NN affiliate safety department within the timelines specified in the Investigator-Initiated Study Agreement (IIS) agreement.. The submission to NN must be within day 15 from the investigator's first knowledge about a valid case. Where required in national law the investigator should also expedite SARs or SUSARs, to Independent Ethics Committee (IEC)/Institutional Review Board (IRB). Treatment codes will be un-blinded for specific participants.

The Investigator must report initial information on all serious AEs to NN within 24 hours of obtaining knowledge about the event. The information must be provided by mail or telephone to the local NN affiliate. The Investigator must complete and forward electronically copies of the AE form and the SAE supplementary pages to NN within 5 calendar days of obtaining knowledge about the SAE.

The sponsor must inform the regulatory authorities and institutional review boards (IRBs)/independent ethics committees (IECs) in accordance with the local requirements in force and ICH GCP. The sponsor will notify the Investigator of trial product related suspected unexpected serious adverse reactions in accordance with the local requirements. In addition, the Investigator will be informed of any trial related procedure SAE which may warrant a change of any trial procedure. Investigators will be notified of trial-related SAEs in accordance with the local requirements in force and ICH GCP. The monitor must be informed accordingly.

Safety follow-up

It will be left to the Investigator's clinical judgment to decide whether or not an AE is of sufficient severity to require the participant's removal from treatment. A participant may also voluntarily withdraw from treatment due to what he or she perceives as an intolerable AE. If either of these occurs, the participant must undergo an end of trial assessment and be given appropriate care under medical supervision until symptoms cease, or the condition becomes stable. Any need for long-term follow-up will be assessed by a physician.

Randomization code breaking

Un-blinding can be performed under the following circumstances:

- Treatment of an individual in a medical emergency where knowledge of the treatment allocation is required.
- Treatment of an individual for an AE.
- In the event of a SUSAR.
- In the event that the participant's study medication is accidentally taken by a member of their household e.g. a child.
- For the submission of trial data to the Data Monitoring and Safety Committee (DMSC) for the monitoring of safety and/or efficacy.

All individuals directly involved in the trial must not be un-blinded. When the code break is needed, the local NN affiliate safety department must be informed the following points by the representative of the sponsor via email: Date and time of the code break, the reason for that, the actions taken, details of the person(s) involved in the code break.

Protection of humans

Declaration of Helsinki

The Investigator will ensure that this trial is conducted in accordance with the principles of the Declaration of Helsinki.

Guidelines for Good Clinical Practice

The sponsor will ensure that this study is conducted in accordance with relevant regulations and with Good Clinical Practice. The sponsor hereby confirm that the ordinary procedures for quality control and assurance are complied with, cf. sections 3 and 4 of the Danish executive order on GCP and the standard procedures for quality control and quality assurance will be complied with, cf. ICH GCP guidelines.

Approvals

The protocol, informed consent form, participant information sheet and any proposed advertising material will be submitted to an appropriate Research Ethics Committee (REC), Competent Authorities, the Danish Data Protection Agency and host institution for written approval.

Informed consent: after giving extensive oral and written information, a written informed consent form will be obtained from the subjects before initiating any study-related activity.

Safety information in the subject informed consent will be in full accordance with risks and safety information included in the latest updated local Summary of Products Characteristics for Saxenda.

The Sponsor will submit and, where necessary, obtain approval from the above parties for all substantial amendments to the original approved documents.

Participant Confidentiality

Information regarding the study participants is protected by the law of personal data (*Persondataloven).*

The trial staff will ensure that the participants' anonymity is maintained. The participants will be identified only by initials and a participants ID number on the CRF and any electronic database. All documents will be stored securely and only accessible by trial staff and authorized personnel. The Danish Data Protection Agency (*Datatilsynet*) will be notified about the trial. The trial will comply with the Data Protection Act, which requires data to be anonymised as soon as it is practical to do so.

Audits, monitoring and inspection

Direct access to source data/documents, including the permission of investigator to access source data/documents (patient files included), at monitoring, audits and/or inspection from the GCP unit, the Ethics Committee, the Competent Authorities or from Health Authorities of other countries.

Indemnity statement

NN only carries the product indemnity. Investigator institutions, University of Copenhagen and Hvidovre Hospital hold indemnity for patients (this is not covered by NN).

Financial aspect

The study is financed by Synergistic effects of GLP-1 and exercise on immuno-metabolic health (Excellence grant from The Novo Nordisk Foundation (NNF), 5 million DKK) (salary, purchase of materials, running operating expenses and analyses), the Danish Diabetes Academy (PhD scholarship, 550,000 DKK) (salary), Faculty of Health Sciences (PhD scholarship, 1,650,000 DKK) (salary), Helsefonden (200,000 DKK) (salary) and NNF center for basic metabolic research synergy grant (556,000 DKK) (salary, running operating expenses, analyses). The GLP-1 analogue (Liraglutide 3 mg, Saxenda) and placebo is provided by Novo Nordisk (approximately 5 million DKK). Cambridge weight plan diet products for the initial 8 weeks weight loss program is donated by Cambridge weight plan (approximately 1 million DKK). All other expenses, incl. salary as well as the planning and conduct of the study, interpretation of data and writing of the manuscripts, are completely independent of the companies.

In regards to the aspect of insurance, study participants are equated to hospital patients. We will be handing out the pamphlet "Forsikringer, klage- og erstatningsmuligheder".

Practical opportunities for implementation of the project

The clinical trial will be performed at Department of Endocrinology, Hvidovre University Hospital and Department of Biomedical Sciences, University of Copenhagen where all necessary expertise, apparatus, and guidance are present. The sponsor and sub-investigators (Associate Prof Signe Torekov, Prof Jens Juul Holst Prof Sten Madsbad, Prof Bente Stallknecht and Post doc Mads Rosenkilde) has long experience in organizing this type of clinical trials (3; 4; 6; 11; 12; 15; 29-34). The clinical Fellow who will run the flow mediated dilation examinations will be trained in Oxford (Antoniades' group), and the image analysis will be performed in the Oxford's workstations. The contact person of the trial is the sponsor, Signe Torekov.

Publication

Results

The results (positive, negative and inconclusive) will be presented at international conferences as oral presentations or poster presentations. In addition, results will be published in peer-reviewed scientific journals. If not published in journals, the results will be disclosed in other ways, for example on www.clinicaltrals.gov.

Agreements regarding publication of results have been mutually made between sponsor, investigator, sub-investigators and other collaborators.

Authorities

As according to the rules, sponsor will notify the Competent Authorities and the Ethics Committee no later than 90 days after the end of trial.

Biological material and establishment of a bio-bank

All biological material obtained from the study participants will be kept in a research bio-bank. During the meal test, 350 ml of blood is drawn. Of these, 322 ml will be frozen in a bio-bank. When taking fasting blood samples (at week 4, 13, 26 and 39) 75 ml of blood is drawn and frozen in a bio-bank. Glasses are labelled with a subject ID number. For risks and disadvantages see next section. Furthermore, we also collect faecal (sample size vary as it is the study participant who collects and freezes the sample at home), urine (maximum 5 mL/test), saliva (maximum 5 mL/test) and semen samples (maximum 10 mL/test) and fat biopsies (maximum 1 gram/test) which are stored in the bio-bank. From the blood samples we will extract DNA material which will be used for genome analyses.

The purpose of the bio-bank is to be able to analyse all samples at once to avoid large instrumental variations. The research bio-bank is terminated no later than August 1st 2036. After this date, the remains of the material are transferred to a regular bio-bank in Denmark. In order to access this material, a new protocol has to be approved from an ethics committee.

Risks and disadvantages

Applying of periphery venous catheter for taking blood samples can cause transient discomfort, irritation and redness around the puncture site. Participants will be informed of this.

Injection with GLP-1 (Saxenda) may cause mild and transient nausea, which often will vanish after a couple of weeks. Other side effects include dizziness, insomnia (transient) and gall stones. Uncommon/rare side include dehydration, inflamed gall bladder, allergic reactions and reduced kidney function. For full

information about side effects please refer to the summary of product characteristics. There should not be any discomfort to the injection if performed as prescribed.
Some discomfort might be experienced when applying local anaesthesia for the fat biopsy. The biopsy itself should not be uncomfortable.
MRI scans are based on a magnetic field (not radiation) and thus does not lead to side effects or medical risks. Participants suffering from claustrophobia may experience some discomfort. If severe claustrophobia, the participant cannot be scanned.
DEXA scans use radiation with a radiation dose of approximately 0.02 mSv per examination. This dose is very low compared to the background radiation in Denmark (approximately 3 mSv/year).
The FMD ultrasound scan does not use radiation. Applying nitro-glycerine may cause transient headache, dizziness, decrease in blood pressure and increase in heart rate. Half-life of nitro-glycerine is 1-3 minutes. Nitro-glycerine will not be used if systolic blood pressure is under 100 mmHg.

Ethical considerations

All participants are informed orally and in writing about the examinations and written consent is collected. The participants are recruited through newspapers and online media. The discomfort, in regards to the examinations, is minimal. Any discomfort will primarily be experienced when taking blood samples and when applying local anaesthesia for the fat biopsies. The overall blood loss is less than the amount of blood collected when donating blood. Only participants with normal blood percentage will participate and will be offered iron supplements.
Injection with GLP-1 is given with Saxenda injection pen. Saxenda is an approved drug and the dosage is kept within the approved maximum (3.0 mg). Saxenda is safe and the only discomfort might be a transient nausea during the first weeks. Placebo injections should not cause any discomfort. The use of placebo is to minimize bias.
The diet (Cambridge Weight Plan) is used in daily clinical practice, and there are no side effects associated with ingestion of the diet.
The exercise program does not exceed the recommendations from WHO.
The risks that are associated with this study are assessed as minimal. By participating in this project, the participants will contribute with new important knowledge about the interaction between GLP-1 and exercise and their importance for weight loss maintenance and metabolic health.
Overall, we consider that any potential risks and side effects are outweighed by the advantages of achieving new knowledge about weight loss maintenance and health.
If a study participant wish to receive further information about the trial, sponsor Signe Torekov, can be contacted.

Compensation

A payment of 3000 DKK will be given to the participants when they complete the study (complete the final test at week 52). Participants who provide semen and faecal samples (at screening, baseline and week 52) will be given an additional 1000 DKK.
In case of drop-out, no compensation will be given.

Reference List

1. Kelly T, Yang W, Chen CS, Reynolds K, He J: Global burden of obesity in 2005 and projections to 2030. IntJ Obes (Lond) 2008;32:1431-1437

2. Akram DS, Astrup AV, Atinmo T, Boissin JL, Bray GA, Carroll KK, Chitson P, Chunming C, Dietz WH, Hill JO, Jequier E, Komodiki C, Matsuzawa Y, Mollentze WF, Moosa K, Noor MI, Reddy KS, Seidell J, Tanphaichitr V, Uauy R, Zimmet P: Obesity: Preventing and managing the global epidemic. World Health Organization 2000;Technical Report Series

3. Iepsen EW, Lundgren J, Dirksen C, Jensen JE, Pedersen O, Hansen T, Madsbad S, Holst JJ, Torekov SS: Treatment with a GLP-1 receptor agonist diminishes the decrease in free plasma leptin during maintenance of weight loss. Int J Obes 2014;

4. Iepsen EW, Lundgren JR, Hartmann B, Pedersen O, Hansen T, Jorgensen NR, Jensen JE, Holst JJ, Madsbad S, Torekov SS: GLP-1 receptor agonist treatment increases bone formation and prevents bone loss in weight-reduced obese women. The Journal of clinical endocrinology and metabolism 2015:jc20151176

5. Flint A, Raben A, Astrup A, Holst JJ: Glucagon-like peptide 1 promotes satiety and suppresses energy intake in humans. J ClinInvest 1998;101:515-520

6. Faerch K, Torekov SS, Vistisen D, Johansen NB, Witte DR, Jonsson A, Pedersen O, Hansen T, Lauritzen T, Sandbaek A, Holst JJ, Jorgensen ME: Glucagon-Like Peptide-1 (GLP-1) Response to Oral Glucose is Reduced in Pre-diabetes, Screen-detected Type 2 Diabetes and Obesity, and Influenced by Sex: The ADDITION-PRO Study. Diabetes 2015;

7. Wadden TA, Hollander P, Klein S, Niswender K, Woo V, Hale PM, Aronne L: Weight maintenance and additional weight loss with liraglutide after low-calorie-diet-induced weight loss: the SCALE Maintenance randomized study. International journal of obesity (2005) 2013;37:1443-1451

8. Pi-Sunyer X, Astrup A, Fujioka K, Greenway F, Halpern A, Krempf M, Lau DC, le Roux CW, Violante Ortiz R, Jensen CB, Wilding JP: A Randomized, Controlled Trial of 3.0 mg of Liraglutide in Weight Management. N Engl J Med 2015;373:11-22

9. Wing RR, Hill JO: Successful weight loss maintenance. Annual review of nutrition 2001;21:323-341

10. Wing RR, Phelan S: Long-term weight loss maintenance. Am J Clin Nutr 2005;82:222s-225s

11. Rosenkilde M, Reichkendler MH, Auerbach P, Torang S, Gram AS, Ploug T, Holst JJ, Sjodin A, Stallknecht B: Appetite regulation in overweight, sedentary men after different amounts of endurance exercise: a randomized controlled trial. Journal of applied physiology (Bethesda, Md : 1985) 2013;115:1599-1609

12. Rosenkilde M, Auerbach P, Reichkendler MH, Ploug T, Stallknecht BM, Sjodin A: Body fat loss and compensatory mechanisms in response to different doses of aerobic exercise--a randomized controlled trial in overweight sedentary males. American journal of physiology Regulatory, integrative and comparative physiology 2012;303:R571-579

13. Wing RR, Bolin P, Brancati FL, Bray GA, Clark JM, Coday M, Crow RS, Curtis JM, Egan CM, Espeland MA, Evans M, Foreyt JP, Ghazarian S, Gregg EW, Harrison B, Hazuda HP, Hill JO, Horton ES, Hubbard VS, Jakicic JM, Jeffery RW, Johnson KC, Kahn SE, Kitabchi AE, Knowler WC, Lewis CE, Maschak-Carey BJ, Montez MG, Murillo A, Nathan DM, Patricio J, Peters A, Pi-Sunyer X, Pownall H, Reboussin D, Regensteiner JG, Rickman AD, Ryan DH, Safford M, Wadden TA, Wagenknecht LE, West DS, Williamson DF, Yanovski SZ: Cardiovascular effects of intensive lifestyle intervention in type 2 diabetes. N Engl J Med 2013;369:145-154

14. Perseghin G, Price TB, Petersen KF, Roden M, Cline GW, Gerow K, Rothman DL, Shulman GI: Increased glucose transport-phosphorylation and muscle glycogen synthesis after exercise training in insulin-resistant subjects. N Engl J Med 1996;335:1357-1362
15. Auerbach P, Nordby P, Bendtsen LQ, Mehlsen JL, Basnet SK, Vestergaard H, Ploug T, Stallknecht B: Differential effects of endurance training and weight loss on plasma adiponectin multimers and adipose tissue macrophages in younger, moderately overweight men. American journal of physiology Regulatory, integrative and comparative physiology 2013;305:R490-498
16. Schubert MM, Sabapathy S, Leveritt M, Desbrow B: Acute exercise and hormones related to appetite regulation: a meta-analysis. Sports medicine (Auckland, NZ) 2014;44:387-403
17. Liang CP, Han S, Li G, Tabas I, Tall AR: Impaired MEK signaling and SERCA expression promote ER stress and apoptosis in insulin-resistant macrophages and are reversed by exenatide treatment. Diabetes 2012;61:2609-2620
18. Kim Chung le T, Hosaka T, Yoshida M, Harada N, Sakaue H, Sakai T, Nakaya Y: Exendin-4, a GLP-1 receptor agonist, directly induces adiponectin expression through protein kinase A pathway and prevents inflammatory adipokine expression. Biochem Biophys Res Commun 2009;390:613-618
19. Hadjiyanni I, Siminovitch KA, Danska JS, Drucker DJ: Glucagon-like peptide-1 receptor signalling selectively regulates murine lymphocyte proliferation and maintenance of peripheral regulatory T cells. Diabetologia 2010;53:730-740
20. Chaudhuri A, Ghanim H, Vora M, Sia CL, Korzeniewski K, Dhindsa S, Makdissi A, Dandona P: Exenatide exerts a potent antiinflammatory effect. J Clin Endocrinol Metab 2012;97:198-207
21. Hogan AE, Gaoatswe G, Lynch L, Corrigan MA, Woods C, O'Connell J, O'Shea D: Glucagon-like peptide 1 analogue therapy directly modulates innate immune-mediated inflammation in individuals with type 2 diabetes mellitus. Diabetologia 2014;57:781-784
22. Parkner T, Sorensen LP, Nielsen AR, Fischer CP, Bibby BM, Nielsen S, Pedersen BK, Moller HJ: Soluble CD163: a biomarker linking macrophages and insulin resistance. Diabetologia 2012;55:1856-1862
23. Moller HJ, Frikke-Schmidt R, Moestrup SK, Nordestgaard BG, Tybjaerg-Hansen A: Serum soluble CD163 predicts risk of type 2 diabetes in the general population. Clinical chemistry 2011;57:291-297
24. Seufert J, Gallwitz B: The extra-pancreatic effects of GLP-1 receptor agonists: a focus on the cardiovascular, gastrointestinal and central nervous systems. Diabetes Obes Metab 2014;16:673-688
25. Xie L, Jiang Y, Ouyang P, Chen J, Doan H, Herndon B, Sylvester JE, Zhang K, Molteni A, Reichle M, Zhang R, Haub MD, Baybutt RC, Wang W: Effects of dietary calorie restriction or exercise on the PI3K and Ras signaling pathways in the skin of mice. The Journal of biological chemistry 2007;282:28025-28035
26. Cheng S-M, Ho T-J, Yang A-L, Chen IJ, Kao C-L, Wu F-N, Lin JA, Kuo C-H, Ou H-C, Huang C-Y, Lee S-D: Exercise training enhances cardiac IGFI-R/PI3K/Akt and Bcl-2 family associated pro-survival pathways in streptozotocin-induced diabetic rats. International Journal of Cardiology 167:478-485
27. Tousoulis D, Antoniades C, Stefanadis C: Evaluating endothelial function in humans: a guide to invasive and non-invasive techniques. Heart 2005;91:553-558
28. Lee R, Margaritis M, Channon KM, Antoniades C: Evaluating oxidative stress in human cardiovascular disease: methodological aspects and considerations. Curr Med Chem 2012;19:2504-2520
29. Torekov SS, Holst JJ, Ehlers MR: Dose response of continuous subcutaneous infusion of recombinant glucagon-like peptide-1 in combination with metformin and sulphonylurea over 12 weeks in patients with type 2 diabetes mellitus. Diabetes ObesMetab 2013;

30. Torekov SS, Madsbad S, Holst JJ: Obesity - an indication for GLP-1 treatment? Obesity pathophysiology and GLP-1 treatment potential. ObesRev 2011;12:593-601
31. Torekov SS, Kipnes MS, Harley RE, Holst JJ, Ehlers MR: Dose response of subcutaneous GLP-1 infusion in patients with type 2 diabetes. Diabetes ObesMetab 2011;13:639-643
32. Madsbad S, Kielgast U, Asmar M, Deacon CF, Torekov SS, Holst JJ: An overview of once-weekly glucagon-like peptide-1 receptor agonists--available efficacy and safety data and perspectives for the future. Diabetes ObesMetab 2011;13:394-407
33. Iepsen EW, Torekov SS, Holst JJ: Therapies for inter-relating diabetes and obesity - GLP-1 and obesity. Expert opinion on pharmacotherapy 2014:1-14
34. Asmar M, Simonsen L, Madsbad S, Stallknecht B, Holst JJ, Bulow J: Glucose-dependent insulinotropic polypeptide may enhance fatty acid re-esterification in subcutaneous abdominal adipose tissue in lean humans. Diabetes 2010;59:2160-2163
35.Eva W. Iepsen, Julie Lundgren, Sten Madsbad, DMSci, Jens J. Holst, Signe S. Torekov Successful weight loss maintenance includes long-lasting increases in the appetite inhibiting hormones GLP-1 and PYY_{3-36} American Diabetes Association (abstract), Diabetes 2015
36. Bodil J. Christensen, Eva W. Iepsen, Julie Lundgren, Sten Madsbad, Jens J. Holst, Signe S Torekov Sustained weight loss is associated with beneficial health outcomes and behavioural, affective and contextual factors are important psycho-social mediators in successful weight loss maintenance. European Congress of Obesity (abstract) 2015.
37. Fjeldborg K, Christiansen T, Bennetzen M, Moller J, Pedersen SB, Richelsen B. The macrophage-specific serum marker, soluble CD163, is increased in obesity and reduced after dietary-induced weight loss. Obesity (Silver Spring) 2013 Dec;21(12):2437-43.
38. Kaati G, Bygren LO, Pembrey M, Sjostrom M. Transgenerational response to nutrition, early life circumstances and longevity. Eur J Hum Genet 2007 Jul;15(7):784-90.
39. Pembrey ME, Bygren LO, Kaati G, Edvinsson S, Northstone K, Sjostrom M, et al. Sex-specific, male-line transgenerational responses in humans. Eur J Hum Genet 2006 Feb;14(2):159-66.
40. Donkin I, Versteyhe S, Ingerslev LR, Qian K, Mechta M, Nordkap L, et al. Obesity and Bariatric Surgery Drive Epigenetic Variation of Spermatozoa in Humans. Cell Metab 2015 Dec 6.
41. Crane JD, Mottillo EP, Farncombe TH, Morrison KM, Steinberg GR. A standardized infrared imaging technique that specifically detects UCP1-mediated thermogenesis in vivo. Mol Metab 2014 Jul;3(4):490-4.
42. Lee P, Linderman JD, Smith S, Brychta RJ, Wang J, Idelson C, et al. Irisin and FGF21 are cold-induced endocrine activators of brown fat function in humans. Cell Metab 2014 Feb 4;19(2):302-9.
43. Finlayson G, King N, Blundell J. The role of implicit wanting in relation to explicit liking and wanting for food: implications for appetite control. Appetite 2008 Jan;50(1):120-7
44. Flint A, Raben A, Blundell JE, Astrup A. Reproducibility, power and validity of visual analogue scales in assessment of appetite sensations in single test meal studies. Int J Obes Relat Metab Disord 2000 Jan;24(1):38-48.

Synergy effect of the appetite hormone GLP-1 (LiragluTide) and Exercise on maintenance of weight loss and health after a low calorie diet – the *S-LiTE* randomized trial

The Universal Trial Number (UTN) is U1111-1173-3104

Sponsor

Professor MSO, Signe Torekov
Group Leader
Deputy Director of Education
University of Copenhagen
Faculty Of Health Sciences
Department of Biomedical Sciences
Novo Nordisk Foundation Center for Basic Metabolic Research
Blegdamsvej 3B
DK-2200 Copenhagen N
Denmark
TEL +45 35327509/ +45 22983827
torekov@sund.ku.dk

Principal Investigator

Professor, DMSci Sten Madsbad,
Department of Endocrinology,
Hvidovre University Hospital
Kettegård Allé 30, 2650 Hvidovre, Denmark
TEL +45 38622243
Sten.Madsbad@regionh.dk

Sub-investigators

Professor, DMSci Jens Juul Holst, Department of Biomedical Sciences, University of Copenhagen
Professor, DMSci Bente Stallknecht, Department of Biomedical Sciences, University of Copenhagen
MD, PhD student Julie Lundgren, Department of Biomedical Sciences, University of Copenhagen
MSc, PhD student Charlotte Janus, Department of Biomedical Sciences, University of Copenhagen
MSc, research assistant, Simon B. K. Jensen, Department of Biomedical Sciences, University of Copenhagen
MD, PhD student, Christian Rimer Juhl, Department of Biomedical Sciences, University of Copenhagen

Trial centres:
Department of Biomedical Sciences, University of Copenhagen, Blegdamsvej 3B, 2200 Copenhagen N, Danmark
Department of Endocrinology, Hvidovre University Hospital, University of Copenhagen, Kettegård Allé 30, 2650 Hvidovre, Denmark
TEL +45 38622243

Other hospital departments, technical departments and/or institutions involved in the trial:
Division of Cardiovascular Medicine, John Radcliffe Hospital, Oxford University, Headley Way Oxford, OX3 9DU, UK
Novo Nordisk A/S, Novo Allé, 2880 Bagsværd, Denmark
Cambridge Weight Plan Ltd, Clare House, Hunters Road, Corby, Northants, NN17 5JE

GCP

The GCP unit of the University Hospitals of Copenhagen,
Bispebjerg Hospital, Building 51, 3rd floor
Bispebjerg Bakke 23
2400 Copenhagen, Denmark
will ensure that the trial follow GCP guidelines.

Signatures and dates

Sten Madsbad, professor
[illegible]
Endokrinologisk Afdeling 541
Region Hovedstaden
Hvidovre Hospital
Kettegård Allé 30, 2650 Hvidovre

Sten Madsbad
Principle investigator

20/11-2019	20/11-2019
**Signe Torekov**	**Sten Madsbad**
Sponsor	Principle investigator

Summary

Diabetes and obesity are global challenges to our healthcare system as the prevalence of individuals with overweight and/or type 2 diabetes (T2D) reaches more than 1 billion humans. We are approaching the abyss why the need for better and new interdisciplinary-based treatment strategies has never been greater. Diet-induced weight loss maintained with physical activity combined with incretin-based treatment may be such an example. People with obesity often suffer from deteriorating metabolic health, such as impaired glucose tolerance, and furthermore recent studies report that sedentary lifestyle may shift the glucose intolerance of these individuals towards a full-blown T2D diagnosis. Alarmingly, diet-induced weight loss alone does not substantially improve long term metabolic health. The secretion of the gluco-regulatory hormone glucagon-like peptide-1 (GLP-1) is significantly attenuated in both obesity and pre-diabetic individuals and GLP-1R agonists are currently being used in the clinic for treating T2D and recently also obesity. Our aim is to prevent weight regain and potentially reverse deterioration of metabolic health in obese individuals glancing at a high risk of developing metabolic disease. We hypothesize that by reversing sedentary lifestyles in obese and potentially pre-diabetic and metabolic syndrome participants with diet-induced weight loss maintained with habitual physical activity and additionally treating these individuals with a GLP-1R agonist their potential improvement in their weight, body composition and metabolic health may reach synergistic levels compared to either of these treatments alone. To investigate this, we will perform a randomized clinical trial with 4 intervention arms: Placebo, physical activity, daily subcutaneous injection with a GLP-1R agonist (liraglutide 3mg) and finally physical activity plus injection with a GLP-1R agonist (liraglutide 3mg). We believe that improved integration of interdisciplinary-based treatment therapies such as an initial diet-induced weight loss maintained with the combination of physical activity and GLP-1R agonist in individuals struggling with obesity may provide substantial improvement on their body composition and metabolic health.

Background and significance

Obesity is a common disorder, and in many western countries and the United States more than half of the populations are overweight or obese. The prevalence is increasing dramatically, and the number of obese individuals worldwide is now projected to reach as much as 1.1 billion by 2030 (1). As obesity is associated with a high rate of morbidity and mortality, and thereby accounts for a large part of the public health care expenses (2), there is a great interest in treatment of obesity. Usually most obese people experience several successful weight losses, however only few are able to maintain the weight loss over a longer period (ex. one year). Furthermore, obesity involves both metabolic and inflammatory disturbances and diet-induced weight loss alone does not substantially improve long term metabolic health (3; 4).

Glucagon-like-peptide-1 (GLP-1) is a hormone that is secreted from specific endocrine L-cells in the intestine upon meal intake. GLP-1 inhibits appetite and reduces food intake (5). Obese people have low endogenous GLP-1 response (6) and weight loss induces a marked increase in GLP-1 response (35). We have recently shown that treatment with GLP-1 analogues (liraglutide 1.2 mg) can facilitate long term weight loss maintenance (12 kg) with substantial improvement in metabolic health, in contrast to similar diet induced weight loss maintenance (3; 4; 7). Moreover, once daily injections of the GLP-1 analogue (liraglutide 3 mg) has been shown to facilitate substantial long-term weight loss (1 year) of approximately 8.0% in individuals with obesity (8).

On the other hand regular physical activity has also been put forward as effective lifestyle component for weight loss maintenance (9) and recent data show that maintainers of weight loss are more likely to perform regular exercise as opposed to individuals that regain weight loss (10)(36). In response to 3 months of daily endurance exercise we recently found no increase in appetite in overweight males (11) despite the exercise program induced significant weight loss and reduction of fat mass (4 kg) (12). Also, The SCALE maintenance trial showed that liraglutide (3 mg), with diet and exercise recommendations, maintained weight loss achieved by caloric restriction and induced further weight loss over 56 weeks (7). Indeed, long term intensive lifestyle intervention with a strong exercise component also results in clinical meaning full weight loss with the same magnitude (8.6%) for individuals with obesity as is observed with GLP-1 analogues (13). While exercise is known to improve the sensitivity to a variety of endocrine functions, e.g. insulin sensitivity (14), the combined and/or synergistic effects of liraglutide and exercise for weight loss maintenance and metabolic health are not known.

Physical exercise influences systemic and adipose tissue inflammatory pathways differently than diet-induced weight loss, and weight loss with physical activity has been suggested to induce a more healthy immuno-metabolism profile compared to diet-induced weight loss alone (15). An acute bout of physical activity has been shown to accentuate the hormonal meal-induced satiety response, e.g. increase in GLP-1(16). Interestingly, emerging evidence also places GLP-1 as a potential important immune metabolic modulator. GLP-1R agonists reduce endoplasmic reticulum stress and decrease inflammation-associated gene and protein expression in macrophages (17) as well as exerting anti-inflammatory actions in adipocytes (18). The GLP-1 analogue, liraglutide, exerted anti-inflammatory actions in endothelial cells (19). In humans, treatment with exenatide, a GLP-1R agonist, has shown potent anti-inflammatory effects (20). Whether these actions are due to GLP-1 receptor activation, or the weight lowering actions of GLP-1R agonists, remain uncertain. Notably, a small study of 10 obese participants with T2D treated with liraglutide for 8 weeks indicated that GLP- 1 analogue therapy directly modulates innate immune-mediated inflammation (21), independent of weight loss, by significantly decreasing the novel marker of macrophage activation: the soluble form of haptoglobin–haemoglobin receptor CD163 (sCD163). In obesity, macrophages become pro-inflammatory, and removal of CD163 becomes up-regulated and is measurable as soluble CD163. Interestingly, sCD163 is strongly associated with insulin resistance independently of inflammatory markers such as TNF-α(22) and recently, sCD163 was found to be a strong predictor of the development of type 2 diabetes in a large Danish cohort as well as a novel marker for the coronary atherosclerotic burden (23). Interestingly, the combination of diet-induced weight loss and exercise decreased sCD163 levels (37). Thus, sCD163 may reflect the link between low-grade inflammation and metabolic abnormalities in the obese state.

Although GLP-1 analogues (like liraglutide) have a global anti-inflammatory potential seen even as early as 8 weeks after initiation of treatment(20; 21), it is unclear whether their potential effect on the vascular wall is mediated by the GLP-1 receptor (GLP-1R), changes in insulin levels or the weight loss induced by these agents. Post-GLP-1R signalling has multiple immuno-metabolic effects (increased sCD163, activation of PI3K/Akt signalling and suppression of MAPK in vascular cells (24), which is also observed with exercise (25; 26)) implying that GLP-1 analogues and exercise may supress vascular oxidative stress, reducing cardiovascular complications of obesity. In addition, GLP-1 analogues and exercise may shift the secretory profile of human

adipose tissue towards an anti-inflammatory state exerting additional protective effects on the cardiovascular system.

Thus, exercise and GLP-1 analogues both seem to facilitate weight loss maintenance and reduce systemic inflammation and may thus be used synergistically as obesity treatment to improve both inflammatory and metabolic health.

Research question

Study Hypothesis

We hypothesize that there is a beneficial effect on maintenance of weight loss and health outcomes when combining treatment with the appetite hormone GLP-1 (liraglutide) and an exercise program in individuals with obesity.

Objective

To investigate the maintenance of weight loss, body composition and health outcomes (glucose and insulin for HOMA-IR and Matsuda index, HbA1c, lipids (i.e. cholesterol, HDL, LDL, and triglycerides), determination of glucose-tolerance status, and biomarkers of inflammation over 1 year with GLP-1 treatment (liraglutide), exercise treatment and the combination in obese persons that have lost at least 5 % of their body weight by low calorie diet during 8 weeks.

Research design and Methods

Study population

Recruitment of 222 participants with obesity (ideally with equal number of men and women) in local newspapers, via electronic media and from Department of Endocrinology, Hvidovre University Hospital as well as Department of Biomedical Sciences, University of Copenhagen.

Inclusion criteria:

- BMI > 32 and < 43 (kg/m^2)
- Age >18 and <65 year
- Safe contraceptive method.

Exclusion criteria:

- Patients diagnosed with *known* serious chronic illness including type 1 or 2 diabetes (or a randomly measured fasting plasma glucose > 7 mmol/l)
- Angina pectoris, coronary heart disease, congestive heart failure (NYHA III-IV)
- Severe renal impairment (creatinine clearance (GFR) <30 mL/min)
- Severe hepatic impairment
- Inflammatory bowel disease
- Diabetic gastroparesis
- Cancer
- Chronic obstructive lung disease
- Psychiatric disease, a history of major depressive or other severe psychiatric disorders
- The use of medications that cause clinically significant weight gain or loss
- Previous bariatric surgery
- A history of idiopathic acute pancreatitis

- A family or personal history of multiple endocrine neoplasia type 2 or familial medullary thyroid carcinoma
- Osteoarthritis which is judged to be too severe to manage the exercise programme. As intended per study design the intervention will include a 5% weight loss prior to randomization, thus it is expected that possible participants with mild form of osteoarthritis will be able to manage exercise prescriptions.
- Pregnancy, expecting pregnancy or breast feeding. If a study participant is in doubt whether she could be pregnant, a urine pregnancy test is performed. Females of childbearing potential who are not using adequate contraceptive methods (as required by local law or practice). Adequate contraception must be used throughout the study period and at least 65 hours after discontinuation of trial medication (65 hours corresponds to 5 times the half-life of Saxenda). Allergy to any of the ingredients/excipients.
- Allergy to any of the ingredients/excipients of the study medication: liraglutide, disodium phosphate dihydrate, propylene glycol, phenol, hydrochloric acid, sodium hydroxide.
- Regular exercise training at high intensity (e.g. spinning) >2 hours per week.

Withdrawal criteria: The participant may withdraw at will at any time. If a randomized participant withdraws, we will offer to perform the investigations planned for week 52 at the time for the originally planned follow-up visit at week 52. The participant may be withdrawn from the trial at the discretion of the Investigator due to a safety concern or if judged non-compliant with trial procedures.
A participant must be withdrawn if the following applies: Pregnancy or intention to become pregnant.

Participant replacement: Withdrawn participants will not be replaced. However, re-screening is allowed within the recruitment period, at the Investigator's discretion.

Reminders: To minimize loss to follow-up, reminders (in the form of text messages or letters) will be sent to the participants prior to the examination dates.

Follow-up procedure:
In case of drop-out, the participant will be asked to complete a full set of examinations for week 52 (V3) at the time where the V3 was planned to be completed.

Study design
Randomized controlled parallel groups clinical trial. The trial is double blinded in regards to study medication. Exercise cannot be blinded for the participants, however, study personnel performing the exercise tests are blinded in regards to randomization to exercise program. Study design and work flow is outlined in figure 1 and 2.
This trial will recruit 222 participants with obesity. The weight loss is obtained during 8 weeks with a very low calorie diet (VLCD) (800 kcal/day), Cambridge diet, which is a well-known initial treatment of obesity (3), with the objective to lose at least 5 % body weight (expected to be achieved in at least 180 participants, and less than 20 will drop out during this phase based on our prior studies(3; 4)). Compliance with the dietary maintenance program will be assessed each week at weight consults with a dietician/scientific personnel during the weight loss period. After 8 weeks of weight loss the participants are randomized to receive a)

Liraglutide 3mg/day sc or b) placebo sc or c) Exercise + placebo sc or d) Exercise + Liraglutide 3mg/d sc for 52 weeks. Weekly/monthly consultations, weighing and collection of fasting blood samples will be continued in all 4 groups during the study period.

Liraglutide or placebo intervention: Daily injections (3mg) with diet/weight consultations starting at dose of 0.6 mg injections with 0.6 mg increments weekly until 3.0 mg is achieved. For participants who do not tolerate the fast weekly up-titration of 0.6 mg study drug until the 3mg, the titration procedure can be prolonged. Participants who do not tolerate the 3mg dose may in special circumstances stay at 2.4 mg, however the overall aim is to reach 3 mg for all study participants. The dosage and up-titration follow the recommendations from the summary of product characteristics.

Exercise intervention: Health authorities (WHO) recommend exercise 30 min/day 5 days/week. To make our study possible, we aim for 4 exercise days per week (150 min/week) after a ramp-in period. Exercise prescription will be performed under strict control of the scientific personnel. 2 sessions per week will be performed under supervision of the staff and 2 sessions will be performed individually but monitored by the staff. *Week 1-2*: 1 supervised session/week, *week 3-4*: 1 supervised and 1 individual session/week, *week 5-6*: 2 supervised and 1 individual sessions/week, *week 7-52*: 2 supervised and 2 individual sessions/week. If this up regulation of exercise is not possible (for example due to side effects of study medication), up regulation will proceed more slowly. Supervised sessions include structured exercise with a duration of 45 min. Of this 30 min will comprise of interval based spinning session and 15 min circuit training program focusing on large muscle groups. Individual exercise includes general physical activity such as brisk walking or cycling to work. Target exercise intensity will be ~80% maximal heart rate during endurance exercise, and is expected to accumulate an average exercise intensity of ~70% maximal heart rate (equivalent to 60% VO_2peak). Participants will use heart rate monitors during exercise sessions. Furthermore, participants will be instructed to perform mostly non-weight bearing activities in the start of the intervention, such as cycling and rowing but brisk walking on a treadmill with inclination may also be performed.

Liraglutide+exercise: Combination of the two groups.
The trial ends at week 52 where Saxenda/placebo and exercise treatment is discontinued. Saxenda/placebo does not need to be phased out. Elimination half-life of Saxenda is approximately 13 hours.

Randomization

The participants are randomized to 1 of the 4 treatment arms in a 1:1:1:1 ratio, according to the subject randomization list (SRL) provided by Novo Nordisk. A qualified un-blinded study nurse (not otherwise associated with the trial) will allocate study participants according to the SRL. Randomization will be stratified by sex and age. Two lists are made; one for participants over 40 years and one for participants under 40 years of age. Males will be gives the lowest available number from the appropriate list (from the top of the list) and females will be gives the highest available number from the appropriate list (from the bottom of the list).

Novo Nordisk also provides a total dispensing unit number list (TDL). The un-blinded study nurse will allocate trial medication using the TDL by matching a six digit Dispensing Unit Number (DUN) to the correct treatment. Each box will be labeled with a unique DUN. The DUN alone is not un-blinding. Thus, the

dispensing of trial medication to participants can be carried out by blinded trial staff, by selecting the DUN(s) provided by the un-blinded study nurse. The SRL and TDL are to be kept with restricted access only to the designated un-blinded study nurse.

Un-blinding
The subject randomization list (SRL) and the total dispensing unit number list (TDL) is stored on site at Hvidovre Hospital. Subject number of the trial participant is matched with the SRL and TDL, which reveals if trial medication is liraglutide or placebo. Preferably, the un-blinded study nurse will perform any un-blinding of study participants. However, if needed, all trial staff (sponsor, investigator and sub-investigators) can get access to the SRL and TDL and perform the un-blinding procedure.

Examinations
Pre-screening
- Individual conversation obtaining medical history, including use of medication
- Measurement of weight, height, waist and hip circumference, blood pressure and pulse
- Measurement of fasting blood glucose, e.g. with HemoCue

V0/screening (before weight loss)
- Body weight, height, waist and hip circumference, blood pressure and pulse
- Meal tests (with liquid meal) are performed and blood samples will be obtained before, during and 3 hours after meal intake for plasma/serum samples (glucose, insulin, C-peptid, leptin, soluble leptin receptor, GLP-1, PYY, ghrelin, glucagon, adiponektin, free fatty acids, sCD163, hsCRP, IL-1, IL-1Rap IL-6, TNF-α, SAA1, SAA2, ORM1, ORM2, malonyldialdehyde, F2-Isoprostanes, CTX and P1NP, FGF21) and peripheral blood mononuclear cells (PBMNCs) isolation, including DNA collection.
  Here amongst, a set of standard samples are collected and analysed on the same day. These include haemoglobin, free calcium, creatinine and GFR, potassium, sodium, CRP, ALAT, amylase, alkaline phosphatase, vitamin D, HbA1c, PTH, TSH, total cholesterol, HDL, LDL, VLDL, and triglycerides. Among other, we use these for monitoring the safety of the study participants.
- Endothelial function of the brachial artery will be evaluated using flow mediated dilatation (FMD) (27) and ECG's are obtained. Carotid intima-media thickness (IMT) is measured by ultrasound
- Body composition (fat mass, fat free mass) will be measured with DEXA scan
- Subcutaneous adipose tissue biopsies (abdominal) are taken under local anaesthesia.
- Faecal, urine, saliva and semen samples are collected.
- Food preferences determined by standardized pictures
- Subjective appetite sensations using visual analogue scales (VAS)
- Questionnaires will be given to the participants to determine 1) self-rated quality of life (SF-36), 2) eating habits (three-factor eating questionnaire), 3) IPAQ, 4) self-efficacy (G-SES), 5) sleep quality (PSQI)
- Physiological tests: we will perform a peak oxygen test, a strength test and a stair test.
- Measurement of sleep and physical activity levels during 7 consecutive days and nights assessed by wrist-worn accelerometry

We aim at completing all tests on one day. If this is not possible, the remaining tests are performed at an adjacent test day.

V1/baseline (after weight loss phase; randomization): Same tests as described for V0.

V2/mid (26 weeks after randomization)

- Body weight, height, waist and hip circumference, blood pressure and pulseBlood samples
- Subcutaneous adipose tissue biopsies (abdominal) are taken under local anaesthesia.
- Accelerometer device (GENEActiv) worn on wrist for 7 consecutive days and nights after follow-up testing to assess physical activity levels

V3/end (52 weeks after randomization): Same tests as described for V0.

Furthermore, fasting blood samples are drawn at week 4, week 13 and week 39.

V4/follow-up (1 year after intervention we will invite participants to a follow-up visit via telephone or e-mail):

- Body weight, height, waist and hip circumference, blood pressure and pulse
- Fasting blood samples (identical to samples obtained for V0)
- DEXA scan
- Questionnaires as in V0
- Accelerometer device (GENEActiv) worn on wrist for 7 consecutive days and nights after follow-up testing to assess physical activity levels

Criteria for when tests can be performed:

- The participant has to be fasting from the previous evening (from 22:00), including food, liquids and medication (except study medication)
- Study medication should be taken on the morning of the tests
- Training should not be performed the day before tests

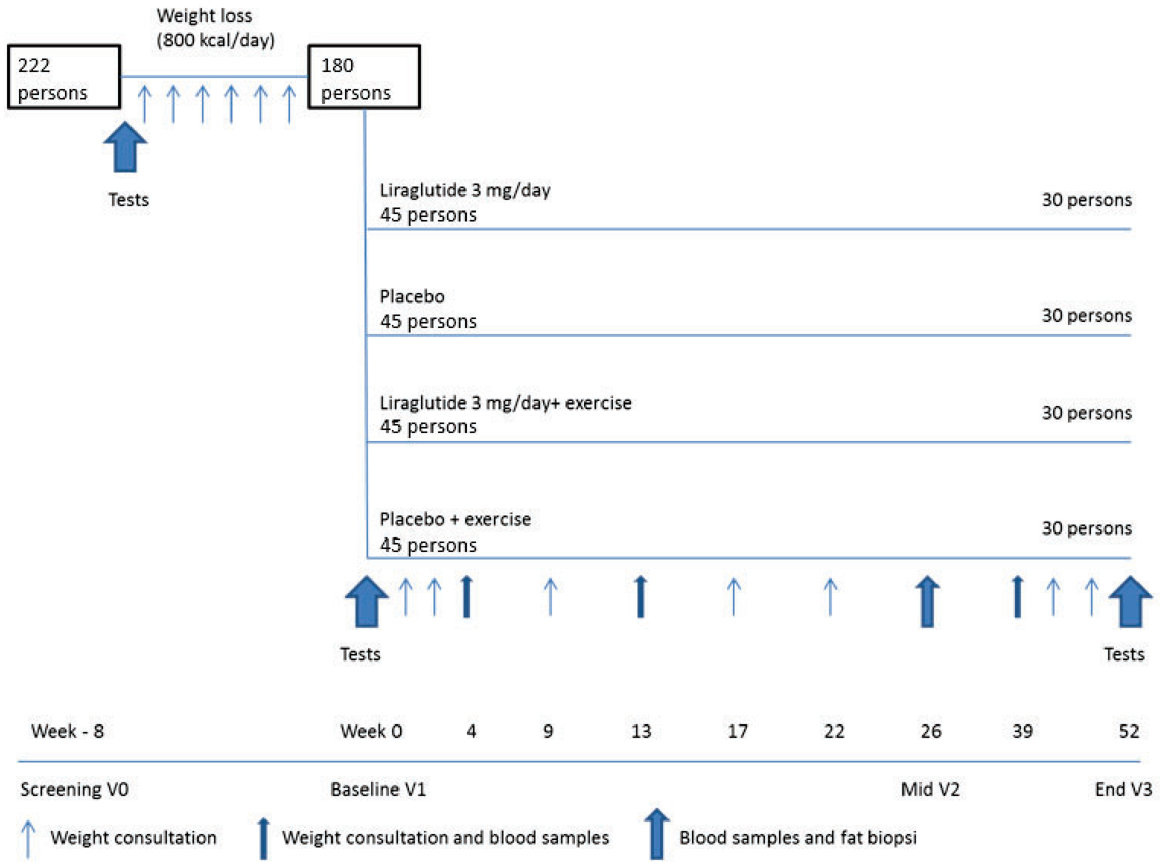

Figure 1 Study design; *Tests* refer to all the tests/examinations described in the *Examination* section for V0.

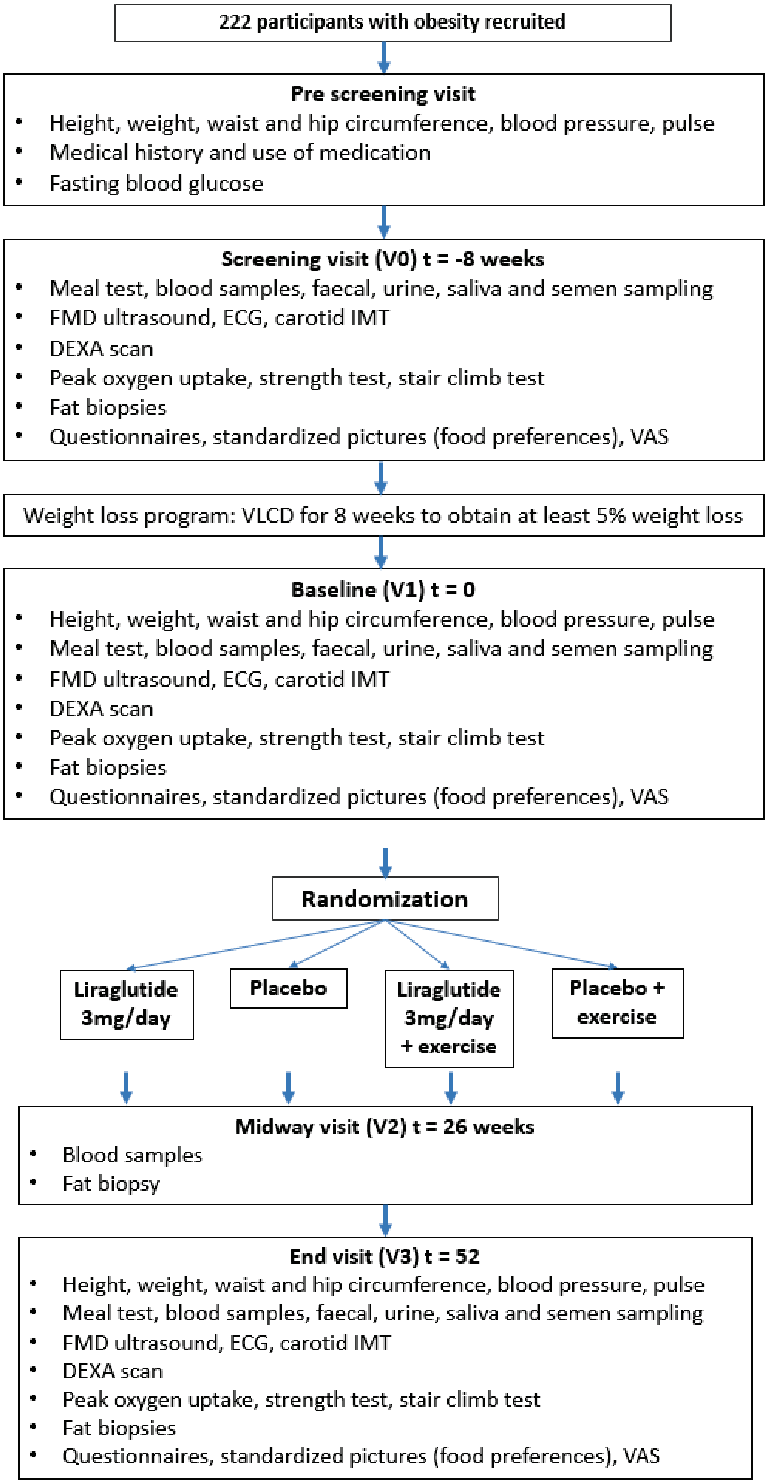

Figure 2 Work flow (FMD: flow mediated dilatation, IMT: intima-media thickness, VAS: visual analogue scale, VLCD: very low calorie diet (800 kcal/day))

Endpoints

Primary endpoints (change from V1 to V3)
(1) Body weight (kg)
Weight will be measured to the nearest 0.1 kg. The same set of scales should ideally be used throughout the trial. Weight should be measured in a fasting state without shoes and wearing light clothes.

Secondary endpoints (change from V1 to V3)
(1) Body composition (fat mass (kg and %) and fat free mass (kg).
Body composition will be measured to nearest 0.1 kg or percentage on a dual-energy X-ray absorptiometry (DXA) scanner. Body composition should be measured in a fasting state without shoes and wearing light clothes.
(2) Metabolic health (glucose tolerance and lipid status, waist circumference, blood pressure, Composite Metabolic syndrome Z-score).

Exploratory endpoints (change from V1 to V3)
(1) Meal related appetite response (2) Peak oxygen uptake and determination of daily physical activity and sleep (3) Systemic markers of immune-metabolism and oxidation (4) Endothelial function (5) Immuno-metabolic changes in the subcutaneous adipose tissue (6) gene expression profile of circulating inflammatory cells (7) Bone health (8) Food preference questionnaire (LFPQ) and subjective appetite sensation (9) Faecal bacterial composition (10) Plasma metabolomics and proteomics (11) epigenetics of spermatozoa. (12) Questionnaries (SF-36, TFEQ, IPAQ, G-SES, PSQI) (13) Use of medication (n, frequency).

Specific aims and endpoints
<u>Primary objective</u>: To investigate the long-term effects of liraglutide and exercise program on the maintenance of weight loss after a very low calorie restriction diet: This will be explored by weekly/monthly weighing of participants.

<u>Secondary objective</u>: To explore the long-term effects of liraglutide and exercise program onbody composition (fat/lean mass) and metabolic health. Study participants are DXA scanned at V0, V1 and V3. These scans can be used to determine fat mass, lean mass, and fat percentage. We will measure circulating biomarkers of metabolic regulation to evaluate metabolic health (glucose and insulin for HOMA-IR and Matsuda index, HbA1c, waist circumference, blood pressure, lipids (i.e. cholesterol, HDL, LDL, and triglycerides) and determination of glucose-tolerance status).

<u>Exploratory objective 1</u>: To explore the long-term effects of liraglutide and exercise program on appetite regulation: We will measure hormonal appetite regulation during meal tests and fasting (e.g. GLP-1, Peptide YY, Glucagon, leptin, Ghrelin, adiponectin, etc.) using our standard methodologies (3).
<u>Exploratory objective 2</u>: To explore the long-term effects of liraglutide and exercise program on physical fitness and sleep: Cardiorespiratory fitness (peak oxygen consumption) will be assessed using an electronically braked bicycle and indirect calorimetry at V0, V1 and V3: After warm-up workload is increased with 25 W increments every minute until attainment of VO_2peak, based on the following criteria: Levelling off in oxygen uptake and RER-value above 1.15 or age-predicted heart rate max (220 minus age). Daily

physical activity levels and sleep will be assessed using wrist-worn accelerometer device (GENEActiv). Maximal strength will be measured as maximum voluntary contraction force of the dominant thigh. A stair climb test will be performed to assess physical functioning.

<u>Exploratory objective 3</u>: To explore the long-term effects of liraglutide and exercise program on systemic markers of immune-metabolism and oxidation: In plasma samples from V0, V1, V2, and V3 we will measure various biomarkers of inflammation (sCD163, hsCRP, IL-1, IL-1Rap IL-6, TNF-α, SAA1, SAA2, ORM1, ORM2 etc.) and oxidation (malonyldialdehyde, F2-Isoprostanes, etc.) using methodologies well established in the lab in Oxford (28).

<u>Exploratory objective 4</u>: To explore the long-term effects of liraglutide and exercise program on vascular endothelial function: We will use Flow-mediated vasodilation (FMD) to study changes in endothelial function (27) as well as blood pressure and electrocardiogram (ECG) measurements at V0, V1 and V3. FMD is an endothelium-dependent function, explored by a non-invasive technique, in the brachial artery. The brachial arteries are scanned with high resolution ultrasound imaging at rest and during hyperaemia. Hyperaemia can be induced by inflation and deflation of a sphygmomanometer cuff around the forearm, distal to the site scanned later with ultrasound. Peripheral arteries, including the brachial artery, respond to physical and chemical stimuli by adjusting vascular tone and regulating blood flow.

Increased blood flow in peripheral arteries is leading to increased shear stress stimuli, increased nitric oxide production, and vasodilation. The vasodilatory response of the brachial artery to increased shear stress is called flow mediated dilation (FMD), and reflects the ability of vascular endothelium to produce NO. Endothelial function, defined as FDM is defined as the percentage increase in vessel diameter from baseline conditions to maximum vessel diameter, during hyperaemia. FMD is estimated as the percentage change of the brachial artery diameter from rest, to the vessel diameter 60 seconds after the cuff is released. After a 12 hour fasting period, the participant is positioned in a supine position, in a quiet room, with constant temperature. The arm is placed in a comfortable position for assessing the brachial artery, and must remain under constant conditions for at least 10 minutes.

All vasoactive drugs must be discontinued at least 12 hours before the study, and the patient must not ingest substances that might affect FMD such as caffeine, high-fat food and vitamin C nor smoke, for at least six hours before the study. The FMD includes administration of 400 micrograms of nitro-glycerine. This method has the advantage of being non-invasive and safe with no risk concerns. From the previous studies (27) with 30 study participants in each group we expect to be able to detect a 10% difference between groups. Imaging analysis are made in Oxford.

<u>Exploratory objective 5</u>*: *To explore the long-term effects of liraglutide and exercise program on immunometabolic profile of the human subcutaneous adipose tissue: In adipose tissue biopsies obtained at V0, V1 and V3 we will: a) determine the changes in the gene expression of GLP-1 receptor, and target-genes downstream the GLP-1 receptor that include pro-inflammatory (e.g. IL-6, IL1b, MCP-1, resistin, leptin, chemerin, etc.) and anti-inflammatory (e.g. adiponectin) adipocytokines that could have an endocrine/paracrine effect on cardiovascular function. The changes in the expression of adipocyte differentiation markers such as FAB4, CABPA and PPAR-γ as well as markers of macrophages infiltration (CD163, CD68) and M1/M2 phenotype of them such as CD40, CCR7, CD207 etc. (all by qRTPCR) will also help us understand the potential phenotypic changes in adipocyte differentiation status and inflammatory infiltration after treatment. The immune cells of the fat will be isolated to evaluate macrophage sup-populations and activation status (single cell analysis). The sc adipose tissue (1 g) will be obtained by needle aspiration under local anaesthesia using 5–10 ml 0.5% lidocaine. It has previously been demonstrated that

this procedure does not influence adipocyte metabolism. This is a standard procedure, safe and with local anaesthesia non-painful.

Exploratory objective 6: ***To explore the effects of long-term treatment with liraglutide and an exercise program on*** gene expression profile and single cell analysis of isolated sup-populations of immune cells of circulating inflammatory cells (PBMNCs) obtained at V0, V1, and V3.

Exploratory* objectives 7*: To explore the long-term effects of liraglutide and exercise program on reversal of weight loss induced bone loss:

We will measure bone markers (CTX and P1NP etc) as well as DEXA scans to assess bone-health.

Exploratory objective 8: To explore the effect of liraglutide and/or exercise on food preferences and appetite sensation: Food preferences, in particular liking and wanting of food items, are assessed by a picture display test where standardized pictures of the following 20 food items are shown: pork rib roast, French fries, nuggets, salty crackers, cheese, smoked fillet (cold cuts), omelet, carrots, crisp bread, turkey strips, Danish pastries, vanilla ice cream, milk chocolate, pound cake, cookies, cut fruits, skyr (yoghurt) with berries, sweet licorice, cocoa meringues and wine gum. These 20 food items can be organized into combined and separate food categories. Pictures are displayed in a randomized order and the participants are instructed to rate each individual food item and to choose the three food items they prefer the most in a prioritized order (43).

Subjective appetite sensations will be obtained during a fixed standardized meal using visual analogue scales (VAS) to record hunger, satiety, fullness, prospective food consumption, desire to eat something fatty, salty, sweet or savory, and palatability of the meals (44).

Exploratory* objective 9-11:** ***To explore the effects of long-term treatment with liraglutide and an exercise program on faecal bacterial composition, plasma metabolomics, proteomics profile, and epigenetics of the spermatozoa at V0, V1 and V3. Epidemiological data support that paternal nutritional status can directly affect the health of the offspring (38;39), suggesting that an environmentally acquired phenomenon of epigenetic inheritance is passed on by the gametes. Recently, Barres showed that spermatozoa of obese men carry altered epigenetic signals specifically located at regions controlling the development of the central nervous system and the control of appetite. Moreover, they showed that weight loss, induced by bariatric surgery, remodels the epigenetic profile of spermatozoa, suggesting that pre-conceptional behaviour targeting weight loss may be relevant to the metabolic health of the next generation (40). We will purify motile spermatozoa by using a so-called “swim-up” method by which motile spermatozoa will be separated from somatic and non-motile spermatozoa. We will lysate the purified, motile spermatozoa from the upper fraction and use this lysate for DNA and RNA extraction. The DNA will be purified and we will use this to determine the DNA methylation and histon modification. We will measure the entire genome promoter DNA methylation by using MethylMinerTM and next-generation sequencing, which subsequently will be verified. The hereby generated data will provide information about potential differences in DNA methylation of spermatozoa before and after weight loss and treatment. The histon modification will also be analysed. Total RNA from spermatozoa will be purified and small-RNAs from total RNA will be separated by gel electroforese as well as ligate adapters to small-RNAs on their 3’ and 5’ ends. Subsequently, reverse transcription and PCR will be applied to produce a complete cDNA library that can be used for next-generation sequencing. This procedure will generate a library of the total content of small-RNAs in human spermatozoa, and this will show potential differences in the expression of small-RNAs in spermatozoa before and after weight loss and treatment. See also the section about ethical considerations.

Exploratory* objective 12:** ***To explore the effects of long-term treatment with liraglutide and an exercise program on self-rated eating habits, physical activity, self-efficacy and sleep quality assessed from validated questionnaires (TFEQ, IPAQ, G-SES, and PSQI).
Exploratory* objective 13:** ***To explore the effects of long-term treatment with liraglutide and an exercise program on use of medication reported from self-reporting as indication, dose, and frequency.

Data

Data management

The participant and the biological material obtained from the participant will be identified by subject number and trial identification number. Appropriate measures such as encryption or deletion will be enforced to protect the identity of human participants in all presentations and publications as required by national requirements.
Laboratory data will be transferred electronically from the laboratory performing clinical analyses and will be archived in secured hard drives with backup options. The electronic data will be considered source data. In cases where laboratory data is transferred via non-secure electronic networks, data will be encrypted during transfer. Data is saved for 20 years.

Source data

Source documents will be kept in the Investigators file.
Source data registered directly in the CRF include blood pressure, weight, height, hip and waist measurements, anamnesis and adverse events.

Evaluability of participants for analysis

Withdrawn participants will not be replaced. However, re-screening is allowed within the recruitment period, at the Investigator's discretion.

Two analysis sets are defined

Per Protocol (PP) analysis set: All exposed participants who complete the 52-week randomized treatment period without significantly violating the inclusion/exclusion criteria or other aspects of the protocol considered to potentially affect the efficacy results.
Intent to treat (ITT) analysis set: All randomized participants.
Safety analysis will be performed on the ITT population.
The decision to exclude any participant or observation from the statistical analysis is the responsibility of the sponsor and will be agreed on before breaking the blind. The participants or observations to be excluded, and the reasons for their exclusion must be documented and signed by those responsible prior to database release. The documentation must be stored together with the remaining trial documentation.
Analysis will be based on the ITT analysis set and PP analysis set. Analysis of the efficacy variables will also be performed on the PP analysis set as supportive evidence.

Data analysis plan

Changes in variables (from week 0 - week 52) will be analysed using a linear mixed model with age, sex, baseline weight (or BMI), and initial weight loss (if relevant) as covariates and treatment as an effect. The

effect of treatment on serial measurements (before, during, and after treatment in the four groups) will be assessed by repeated measures analyses as appropriate (linear mixed model with an appropriate covariance structure and a suitably described effect over time). We will use time, group allocation and their interaction to investigate the effects of drug and exercise. Adequacy of model assumptions will be assessed using graphical models, and outcome variables may be logarithmically transformed if considered necessary to meet the assumptions of linearity, variance homogeneity, and/or normality of residuals.

Statistical methods
All two-sided tests are at the 5% significance level.

Primary endpoint
The change from baseline (randomization) in body weight (kg) to 52 weeks of treatment will be analysed using a linear mixed model with treatment group as explanatory variable and baseline weight or BMI, sex, and initial weight loss (if relevant) as covariates. Weight change over time will also be analysed using linear mixed model analysis with an appropriate covariance structure and a suitably described effect over time. The objective of the analyses is further specified in the statistical analysis plan. ITT and PP analyses are performed.

Secondary endpoints
The secondary efficacy endpoints are: Body composition (fat (% and kg), fat free mass (kg)) and metabolic health measured by glucose tolerance and metabolic syndrome z-score. Linear mixed models as described above will be used to test for superiority. The change from baseline (randomization) after 52 weeks of treatment will be analysed using a linear mixed model with treatment group as explanatory variable and baseline weight or BMI, initial weight loss (if relevant), and sex as covariates. Longitudinal data will also be analysed using linear mixed models as described above.

Exploratory endpoints
Exploratory endpoints are described above. The change from baseline (randomization) after 52 weeks of treatment will be analysed using a linear mixed model with treatment group as explanatory variable and baseline weight or BMI, initial weight loss (if relevant), and sex as covariates. Longitudinal data will also be analysed using linear mixed models as described above.

Safety endpoints
Endpoints related to safety are: AEs, laboratory tests (haematology and biochemistry)
AEs: Frequencies of participants experiencing treatment emergent AEs (TEAE) and frequency of the events will be tabulated by system organ class and preferred term. Listings of TEAEs, non-TEAEs, SAEs, and AEs leading to withdrawal will be provided.
A TEAE is defined as an event that
• Occurs after randomization and increases in severity during the treatment period
• Occurs between first and last day of treatment + seven days
Clinical laboratory tests: Haematology and biochemistry will be summarized descriptively together with change from baseline. Abnormal values will be listed.

Sample size calculation

Based on our previous studies (3; 4) only 10 % did not obtain a weight loss of more than 10% during the initial weight loss phase, thus we expect at least 180 out of 222 to obtain at least a 5% weight loss. We are planning a study with 30 experimental participants and 30 control participants. In our previous studies (3; 4) the response within each participant group was normally distributed with standard deviation 5.5. If the true difference in the experimental and control means is 4kg, we will be able to reject the null hypothesis that the population means of the experimental and control groups are equal with probability (power) 0.8. The Type I error probability associated with this test of this null hypothesis is 0.05. If we unexpectedly experience higher withdrawal rates than stated more study participant will be recruited to obtain sufficient power. Retention modalities: by frequent weight and diet consultations with the medical doctor/dietician we have previously experienced that this motivated study participants to fulfil the study period.

Study time line

August 2016 – August 2023

Study medication

The active treatment will be subcutaneous injections of liraglutide 3mg (Saxenda) or placebo, that will be given once daily for 52 weeks after weight loss.

Treatment of participants

Participants will attend a pre-screening visit to assess their eligibility. If found eligible, participants should return at visit 0. When the weight loss program has been completed they will be randomized to one of the four treatment arms.
The randomized treatment period lasts 52 weeks, including at least 5 weeks of dose escalation for the liraglutide/placebo/exercise treatment.

Labels and packaging

Novo Nordisk A/S will handle the labelling and packaging of the study medication. The text of the labels will be according to Eudralex Volume 4, Annex 13.

Accounting of study medication

To account for study medication schemas are used to 1) register reception of medication on site from Novo Nordisk (reception date, number of packages, batch number, expiration date, signatures) and 2) register the medication handed out to study participants (ID number of participant, date, batch number, expiration date, number of pens, number of pens returned, signatures).
For surveillance of compliance in terms of using the study medication, we will register number of returned pens. This will be set in relation to the amount of study medication used in case the medicine is used as prescribed.

Dose escalation and drug administration

Liraglutide will be available at a concentration of 6.0 mg/mL and supplied in 3 mL pen-injectors.
Dosing with the liraglutide pen-injector is controlled by turning the dose selector until the dose indicator lines up with the relevant dose. Liraglutide is administered once daily by subcutaneous injections with the pen-

injector, either in the abdomen, thigh or upper arm. Injections can be done at any time of the day and irrespective of meals. It is recommended that the time of administration is repeated between consecutive days throughout the trial. Participants will be instructed to perform an air shot before the first use of a new pen-injector. For further information, please see the handling instructions for the liraglutide pen-injector. These handling instructions will be provided in Danish language together with the trial products. The Investigator must instruct participants how to inject liraglutide, and must ensure that the participants are familiar with the instructions. Participants will be instructed to escalate the liraglutide/placebo dose to 3 mg/day over a five week period, following an initiation dose of 0.6 mg/day and weekly dose escalation steps of 0.6 mg/day or more slowly if side-effects occur.

Storage of trial products

The liraglutide pen-injectors must be stored in a refrigerator at a temperature between + 2°C and + 8°C. Freezing must be avoided and the drug must not be used if it has been frozen. Liraglutide pen injectors must be protected from all sources of light, and the pen cap should be kept on when the pen is not in use. The in-use time for liraglutide is 1 month at temperatures below 30°C.

Trial medication will be stored in a locked room, separately of other medicines.

Concomitant illnesses and medication

Concomitant illness: any illness that is present at the start of the trial (i.e. at the first visit).

Concomitant medication: any medication other than the trial products that is taken during the trial, including the screening and run-in periods.

Details of all concomitant illnesses and medication must be recorded at trial entry (i.e. at the first visit). Any changes in concomitant medication must be recorded at each visit. If the change influences the participant's eligibility to continue in the trial, then the sponsor must be informed.

The information collected for each concomitant medication includes, as a minimum, start date, stop date or continuing and indication.

Safety (see appendix: SAFETY REPORTING REQUIREMENTS FOR INVESTIGATOR INITIATED STUDIES (IIS))

Definitions

Adverse Event (AE)	Any untoward medical occurrence in a participant to whom a medicinal product has been administered, including occurrences which are not necessarily caused by or related to that product. An Adverse Event (AE) can therefore be any unfavourable and unintended sign (including an abnormal laboratory finding), symptom, or disease temporally associated with the use of a product, whether or not considered related to the product.
Adverse Reaction (AR)	An untoward and unintended response in a participant to an investigational medicinal product which is related to any dose administered to that participant.<br>The phrase "response to an investigational medicinal product" means that a causal relationship between a trial medication and an AE is at least a reasonable possibility, i.e. the relationship cannot be ruled out.

	All cases judged by either the reporting medically qualified professional or the Sponsor as having a reasonable suspected causal relationship to the trial medication qualify as adverse reactions.
Serious Adverse Event (SAE)	A serious adverse event is any untoward medical occurrence that:<br>• results in death<br>• is life-threatening<br>• requires inpatient hospitalization or prolongation of existing hospitalization<br>• results in persistent or significant disability/incapacity<br>• consists of a congenital anomaly or birth defect.<br>Other 'important medical events' may also be considered serious if they jeopardize the participant or require an intervention to prevent one of the above consequences.<br>NOTE: The term "life-threatening" in the definition of "serious" refers to an event in which the participant was at risk of death at the time of the event; it does not refer to an event which hypothetically might have caused death if it were more severe.<br>Suspicion of transmission of infectious agents must always be considered an SAE.
Serious Adverse Reaction (SAR)	An adverse event that is both serious and, in the opinion of the reporting Investigator, believed with reasonable probability to be due to one of the trial treatments, based on the information provided.
Suspected Unexpected Serious Adverse Reaction (SUSAR)	A serious adverse reaction, the nature and severity of which is not consistent with the information about the medicinal product in question set out:<br>• in the case of a product with a marketing authorization, in the summary of product characteristics (SmPC) for that product<br>•

NB: to avoid confusion or misunderstanding of the difference between the terms "serious" and "severe", the following note of clarification is provided: "Severe" is often used to describe intensity of a specific event, which may be of relatively minor medical significance. "Seriousness" is the regulatory definition supplied above.

Reference Document

The reference document for reporting adverse events is the Summary of Product Characteristics (SmPC) for Saxenda (European Medical Agency, published 16/04/2015). In particular section 4.8 "Undesirable effects".

Pregnancy

If pregnancy unexpectedly occurs the participant is immediately excluded from the trial. Any pregnancy occurring during the clinical trial and the outcome of the pregnancy should be recorded and followed up for congenital abnormality or birth defect, at which point it would fall within the definition of "serious". All pregnancies in trial participants occurring during use of liraglutide will be recorded immediately to NN (Novo Nordisk).

Causality

The relationship of each adverse event to the trial medication must be determined by a medically qualified individual according to the following definitions:
Probable - Good reason and sufficient documentation to assume a causal relationship. Possible - A causal relationship is conceivable and cannot be dismissed. Unlikely - The event is most likely related to aetiology other than the trial product.
The reference document in the protocol which is agreed upon with NN, for evaluation of expectedness will be specified.

Procedures for recording Adverse Events

All adverse events will be actively collected from the first study-related activity (from the signing of the informed consent) and in all following contacts with the study participant throughout the project. This includes events from the first trial related activity after the participant has signed the informed consent, and until the post treatment follow-up period, as defined in the protocol. Trial-related activities are any procedures (i.e. laboratory tests, X-rays, ECGs, fasting periods etc.) that would not have been performed during normal management of the participant.
As a minimum requirement, the sponsor should copy NN when expediting SARs or SUSARs to Competent Authorities and should report all SARs related to NN Product to NN within the timelines specified in the Investigator-Initiated Study Agreement (IIS) agreement.
As a minimum the following details will be reported -Study name -Patient identification (e.g. subject number, initials, sex, age) -Event (Preferably diagnosis) -Trial drug -Reporter -Causality -Outcome These details to be reported to the local NN affiliate safety department.
All AEs occurring during the trial that are observed by the Investigator or reported by the participant will be recorded on the CRF, whether or not attributed to trial medication.
The following information will be recorded: description, date of onset and end date, severity, assessment of relatedness to trial medication, other suspect drug or device and action taken. Follow-up information should be provided as necessary.
The severity of events will be assessed on the following scale: 1 = mild, 2 = moderate, 3 = severe.
AEs considered related to the trial medication as judged by a medically qualified investigator or the Sponsor will be followed either until resolution, or the event is considered stable.

Reporting of adverse events/reactions

SUSARs

We do not expect any SUSARs. The trial medication is approved for treatment of obesity and used in this trial as prescribed in the Summary of Product Characteristics. The study medication has been thoroughly tested in several clinical trials.
In case of any deadly or life-threatening SUSAR, Sponsor will immediately (and no later than 7 days after becoming aware) notify the Competent Authorities (Lægemiddelstyrelsen) and the Ethics Committee. No later than 8 days after reporting of a SUSAR, Sponsor will notify the Competent Authorities and the Ethics Committee of all relevant information about Sponsor and Investigator’s follow-up of the SUSAR. All other SUSARs will be reported to the Competent Authorities and the Ethics Committee no later than 15 days after Sponsor becoming aware of this.

SARs

Investigator will immediately report all SARs to sponsor. The notification will be followed by a detailed written report. Once yearly, Sponsor will prepare a report regarding SARs occurring in the trial as well as describe the safety of the study participants in regards to continuation of the trial. This will be sent to the Competent Authorities and the Ethics Committee. Reporting will follow the EudraLex - Volume 10 Clinical trial guidelines (Detailed guidance on the collection, verification and presentation of adverse event/reaction reports arising from clinical trials on medicinal products for human use (CT 3)).

SAEs

Investigator will immediately report all SAEs to sponsor. The notification will be followed by a detailed written report. If Sponsor upgrades a SAE to a SAR, procedure is as described above.

SAEs, AEs, ARs

Will be noted in the CRF. These will be recorded in the End of Trial Form to the Competent Authorities and in the report to the Ethics Committee (if requested) no later than 90 days after trial completion.

To NN:

As a minimum, the investigator should copy NN (Novo Nordisk) when expediting SARs or SUSARs to health authorities and should report all SARs related to NN product to the local NN affiliate safety department within the timelines specified in the Investigator-Initiated Study Agreement (IIS) agreement.

The submission to NN must be within day 15 from the investigator's first knowledge about a valid case. Where required in national law the investigator should also expedite SARs or SUSARs, to Independent Ethics Committee (IEC)/Institutional Review Board (IRB). Treatment codes will be un-blinded for specific participants.

The Investigator must report initial information on all serious AEs to NN within 24 hours of obtaining knowledge about the event. The information must be provided by mail or telephone to the local NN affiliate. The Investigator must complete and forward electronically copies of the AE form and the SAE supplementary pages to NN within 5 calendar days of obtaining knowledge about the SAE.

The sponsor must inform the regulatory authorities and institutional review boards (IRBs)/independent ethics committees (IECs) in accordance with the local requirements in force and ICH GCP. The sponsor will notify the Investigator of trial product related suspected unexpected serious adverse reactions in accordance with the local requirements. In addition, the Investigator will be informed of any trial related procedure SAE which may warrant a change of any trial procedure. Investigators will be notified of trial-related SAEs in accordance with the local requirements in force and ICH GCP. The monitor must be informed accordingly.

Safety follow-up

It will be left to the Investigator's clinical judgment to decide whether or not an AE is of sufficient severity to require the participant's removal from treatment. A participant may also voluntarily withdraw from treatment due to what he or she perceives as an intolerable AE. If either of these occurs, the participant must undergo an end of trial assessment and be given appropriate care under medical supervision until symptoms cease, or the condition becomes stable. Any need for long-term follow-up will be assessed by a physician.

Randomization code breaking

Un-blinding can be performed under the following circumstances:

- Treatment of an individual in a medical emergency where knowledge of the treatment allocation is required.
- Treatment of an individual for an AE.
- In the event of a SUSAR.
- In the event that the participant's study medication is accidentally taken by a member of their household e.g. a child.
- For the submission of trial data to the Data Monitoring and Safety Committee (DMSC) for the monitoring of safety and/or efficacy.

All individuals directly involved in the trial must not be un-blinded. When the code break is needed, the local NN affiliate safety department must be informed the following points by the representative of the sponsor via email: Date and time of the code break, the reason for that, the actions taken, details of the person(s) involved in the code break.

Protection of humans

Declaration of Helsinki

The Investigator will ensure that this trial is conducted in accordance with the principles of the Declaration of Helsinki.

Guidelines for Good Clinical Practice

The sponsor will ensure that this study is conducted in accordance with relevant regulations and with Good Clinical Practice. The sponsor hereby confirm that the ordinary procedures for quality control and assurance are complied with, cf. sections 3 and 4 of the Danish executive order on GCP and the standard procedures for quality control and quality assurance will be complied with, cf. ICH GCP guidelines.

Approvals

The protocol, informed consent form, participant information sheet and any proposed advertising material will be submitted to an appropriate Research Ethics Committee (REC), Competent Authorities, the Danish Data Protection Agency and host institution for written approval.

Informed consent: after giving extensive oral and written information, a written informed consent form will be obtained from the participants before initiating any study-related activity.

Safety information in the participant informed consent will be in full accordance with risks and safety information included in the latest updated local Summary of Products Characteristics for Saxenda.

The Sponsor will submit and, where necessary, obtain approval from the above parties for all substantial amendments to the original approved documents.

Participant Confidentiality

Information regarding the study participants is protected by the law of personal data (*Persondataloven).*

The trial staff will ensure that the participants' anonymity is maintained. The participants will be identified only by initials and a participants ID number on the CRF and any electronic database. All documents will be stored securely and only accessible by trial staff and authorized personnel. The Danish Data Protection

Agency (*Datatilsynet*) will be notified about the trial. The trial will comply with the Data Protection Act, which requires data to be anonymised as soon as it is practical to do so.

Audits, monitoring and inspection

Direct access to source data/documents, including the permission of investigator to access source data/documents (patient files included), at monitoring, audits and/or inspection from the GCP unit, the Ethics Committee, the Competent Authorities or from Health Authorities of other countries.

Indemnity statement

NN only carries the product indemnity. Investigator institutions, University of Copenhagen and Hvidovre Hospital hold indemnity for patients (this is not covered by NN).

Financial aspect

The study is financed by Synergistic effects of GLP-1 and exercise on immuno-metabolic health (Excellence grant from The Novo Nordisk Foundation (NNF), 5 million DKK) (salary, purchase of materials, running operating expenses and analyses), the Danish Diabetes Academy (PhD scholarship, 550,000 DKK) (salary), Faculty of Health Sciences (PhD scholarship, 1,650,000 DKK) (salary), Helsefonden (200,000 DKK) (salary) and NNF center for basic metabolic research synergy grant (556,000 DKK) (salary, running operating expenses, analyses). The GLP-1 analogue (Liraglutide 3 mg, Saxenda) and placebo is provided by Novo Nordisk (approximately 5 million DKK). Cambridge weight plan diet products for the initial 8 weeks weight loss program is donated by Cambridge weight plan (approximately 1 million DKK). All other expenses, incl. salary as well as the planning and conduct of the study, interpretation of data and writing of the manuscripts, are completely independent of the companies.

In regards to the aspect of insurance, study participants are equated to hospital patients. We will be handing out the pamphlet “Forsikringer, klage- og erstatningsmuligheder”.

Practical opportunities for implementation of the project

The clinical trial will be performed at Department of Endocrinology, Hvidovre University Hospital and Department of Biomedical Sciences, University of Copenhagen where all necessary expertise, apparatus, and guidance are present. The sponsor and sub-investigators (Prof Signe Torekov, Prof Jens Juul Holst Prof Sten Madsbad, and Prof Bente Stallknecht) has long experience in organizing this type of clinical trials (3; 4; 6; 11; 12; 15; 29-34). The clinical Fellow who will run the flow mediated dilation examinations will be trained in Oxford (Antoniades’ group), and the image analysis will be performed in the Oxford’s workstations. The contact person of the trial is the sponsor, Signe Torekov.

Publication

Results

The results (positive, negative and inconclusive) will be presented at international conferences as oral presentations or poster presentations. In addition, results will be published in peer-reviewed scientific journals. If not published in journals, the results will be disclosed in other ways, for example on www.clinicaltrials.gov.

Agreements regarding publication of results have been mutually made between sponsor, investigator, sub-investigators and other collaborators.

Authorities

As according to the rules, sponsor will notify the Competent Authorities and the Ethics Committee no later than 90 days after the end of trial.

Biological material and establishment of a bio-bank

All biological material obtained from the study participants will be kept in a research bio-bank. During the meal test, 350 ml of blood is drawn. Of these, 322 ml will be frozen in a bio-bank. When taking fasting blood samples (at week 4, 13, 26 and 39) 75 ml of blood is drawn and frozen in a bio-bank. Glasses are labelled with a participant ID number. For risks and disadvantages see next section. Furthermore, we also collect faecal (sample size vary as it is the study participant who collects and freezes the sample at home), urine (maximum 5 mL/test), saliva (maximum 5 mL/test) and semen samples (maximum 10 mL/test) and fat biopsies (maximum 1 gram/test) which are stored in the bio-bank. From the blood samples we will extract DNA material which will be used for genome analyses.

The purpose of the bio-bank is to be able to analyse all samples at once to avoid large instrumental variations. The research bio-bank is terminated no later than August 1st 2036. After this date, the remains of the material are transferred to a regular bio-bank in Denmark. In order to access this material, a new protocol has to be approved from an ethics committee.

Risks and disadvantages

Obtaining blood for measurement of blood glucose may cause transient slight discomfort. Applying of periphery venous catheter for taking blood samples can cause transient discomfort, irritation and redness around the puncture site. Participants will be informed of this.

Injection with GLP-1 (Saxenda) may cause mild and transient nausea, which often will vanish after a couple of weeks. Other side effects include dizziness, insomnia (transient) and gall stones. Uncommon/rare side include dehydration, inflamed gall bladder, allergic reactions and reduced kidney function. For full information about side effects please refer to the summary of product characteristics. There should not be any discomfort to the injection if performed as prescribed.

Some discomfort might be experienced when applying local anaesthesia for the fat biopsy. The biopsy itself should not be uncomfortable.

DEXA scans use radiation with a radiation dose of approximately 0.02 mSv per examination. This dose is very low compared to the background radiation in Denmark (approximately 3 mSv/year).

The FMD and carotid IMT ultrasound scans do not use radiation. Applying nitro-glycerine may cause transient headache, dizziness, decrease in blood pressure and increase in heart rate. Half-life of nitro-glycerine is 1-3 minutes. Nitro-glycerine will not be used if systolic blood pressure is under 100 mmHg.

Ethical considerations

All participants are informed orally and in writing about the examinations and written consent is collected. With regards to the follow-up visit, oral and written information will be given and a new written informed consent will be obtained before any of the follow-up examinations are performed.

The participants are recruited through newspapers and online media. The discomfort, in regards to the examinations, is minimal. Any discomfort will primarily be experienced when taking blood samples and when applying local anaesthesia for the fat biopsies. The overall blood loss is less than the amount of blood

collected when donating blood. Only participants with normal blood percentage will participate and will be offered iron supplements.
Injection with GLP-1 is given with Saxenda injection pen. Saxenda is an approved drug and the dosage is kept within the approved maximum (3.0 mg). Saxenda is safe and the only discomfort might be a transient nausea during the first weeks. Placebo injections should not cause any discomfort. The use of placebo is to minimize bias.
The diet (Cambridge Weight Plan) is used in daily clinical practice, and there are no side effects associated with ingestion of the diet.
The exercise program does not exceed the recommendations from WHO.
The risks that are associated with this study are assessed as minimal. By participating in this project, the participants will contribute with new important knowledge about the interaction between GLP-1 and exercise and their importance for weight loss maintenance and metabolic health.
Based on the blood, fat, and semen samples, we will perform genomic examinations, which highly unlikely, but in theory can generate accidental findings that can have a considerable impact on the examined test person. However, the risks of generating such findings are considered to be minimal.
Unless the test participant declines it explicitly in the consent, the test participant will be informed about serious genetic conditional disease if:

- there is a certain chance for a genetic disposition to be present;
- there is a well-documented correlation between the genetic disposition and the development of disease;
- the tests that are applied to determine the genetic disposition are well-established and accurate;
- the disease to a certain extent can be prevented or treated; and
- the correlation has a substantial impact on the test participant.

All individuals included in the present protocol are competent adults. In the unlikely event that this research project by change identifies a genetic variant known to cause a serious disease, for which there is treatment and /or prevention option for the project participant, the genetic finding will be verified by Sanger sequencing. Provided that the mutation is confirmed and is known to cause a disease with significant penetrance, the responsible project manager will set up a local genetically-ethics panel consisting of a specialist in molecular genetics, a specialist in clinical genetics with experience in genetic counselling and a specialist in the specific disease that is triggered by the mutation, in order to provide an action and communication plan in relation to the mutation carrier. If the panel is in doubt about the action plan, the genetic findings will presented to the The National Committee on Health Research Ethics. If it is concluded that there are medical reasons (see above) to inform the genetic findings to the project participant, the panel specialist in clinical genetics will contact the project participant and inform about the project. The project participant will in this regard be given the option in writing to opt in or opt out of information on critical health information that may appear in the project. If the project participant wants to be informed, she or he will be offered genetic counselling through the panel specialist in clinical genetics and in consultation with his or hers own general practitioner. All study participants will be informed that an objective of the study, in which they were included, also was examinations of the role of genetics in disease processes related to obesity and diabetes. Overall, we consider that any potential risks and side effects are outweighed by the advantages of achieving new knowledge about weight loss maintenance and health.
If a study participant wish to receive further information about the trial, sponsor Signe Torekov, can be contacted.

Compensation

A payment of 3,000 DKK will be given to the participants when they complete the study (complete the final test at week 52). Participants who provide semen samples (at screening, baseline and week 52) will be given an additional 1,000 DKK.

In case of drop-out, no compensation will be given.

A payment of 1,000 DKK will be given to participants who attend the follow-up visit.

Reference List

1. Kelly T, Yang W, Chen CS, Reynolds K, He J: Global burden of obesity in 2005 and projections to 2030. IntJ Obes (Lond) 2008;32:1431-1437
2. Akram DS, Astrup AV, Atinmo T, Boissin JL, Bray GA, Carroll KK, Chitson P, Chunming C, Dietz WH, Hill JO, Jequier E, Komodiki C, Matsuzawa Y, Mollentze WF, Moosa K, Noor MI, Reddy KS, Seidell J, Tanphaichitr V, Uauy R, Zimmet P: Obesity: Preventing and managing the global epidemic. World Health Organization 2000;Technical Report Series
3. Iepsen EW, Lundgren J, Dirksen C, Jensen JE, Pedersen O, Hansen T, Madsbad S, Holst JJ, Torekov SS: Treatment with a GLP-1 receptor agonist diminishes the decrease in free plasma leptin during maintenance of weight loss. Int J Obes 2014;
4. Iepsen EW, Lundgren JR, Hartmann B, Pedersen O, Hansen T, Jorgensen NR, Jensen JE, Holst JJ, Madsbad S, Torekov SS: GLP-1 receptor agonist treatment increases bone formation and prevents bone loss in weight-reduced obese women. The Journal of clinical endocrinology and metabolism 2015:jc20151176
5. Flint A, Raben A, Astrup A, Holst JJ: Glucagon-like peptide 1 promotes satiety and suppresses energy intake in humans. J ClinInvest 1998;101:515-520
6. Faerch K, Torekov SS, Vistisen D, Johansen NB, Witte DR, Jonsson A, Pedersen O, Hansen T, Lauritzen T, Sandbaek A, Holst JJ, Jorgensen ME: Glucagon-Like Peptide-1 (GLP-1) Response to Oral Glucose is Reduced in Pre-diabetes, Screen-detected Type 2 Diabetes and Obesity, and Influenced by Sex: The ADDITION-PRO Study. Diabetes 2015;
7. Wadden TA, Hollander P, Klein S, Niswender K, Woo V, Hale PM, Aronne L: Weight maintenance and additional weight loss with liraglutide after low-calorie-diet-induced weight loss: the SCALE Maintenance randomized study. International journal of obesity (2005) 2013;37:1443-1451
8. Pi-Sunyer X, Astrup A, Fujioka K, Greenway F, Halpern A, Krempf M, Lau DC, le Roux CW, Violante Ortiz R, Jensen CB, Wilding JP: A Randomized, Controlled Trial of 3.0 mg of Liraglutide in Weight Management. N Engl J Med 2015;373:11-22
9. Wing RR, Hill JO: Successful weight loss maintenance. Annual review of nutrition 2001;21:323-341
10. Wing RR, Phelan S: Long-term weight loss maintenance. Am J Clin Nutr 2005;82:222s-225s
11. Rosenkilde M, Reichkendler MH, Auerbach P, Torang S, Gram AS, Ploug T, Holst JJ, Sjodin A, Stallknecht B: Appetite regulation in overweight, sedentary men after different amounts of endurance exercise: a randomized controlled trial. Journal of applied physiology (Bethesda, Md : 1985) 2013;115:1599-1609
12. Rosenkilde M, Auerbach P, Reichkendler MH, Ploug T, Stallknecht BM, Sjodin A: Body fat loss and compensatory mechanisms in response to different doses of aerobic exercise--a randomized controlled trial in overweight sedentary males. American journal of physiology Regulatory, integrative and comparative physiology 2012;303:R571-579
13. Wing RR, Bolin P, Brancati FL, Bray GA, Clark JM, Coday M, Crow RS, Curtis JM, Egan CM, Espeland MA, Evans M, Foreyt JP, Ghazarian S, Gregg EW, Harrison B, Hazuda HP, Hill JO, Horton ES, Hubbard VS, Jakicic JM, Jeffery RW, Johnson KC, Kahn SE, Kitabchi AE, Knowler WC, Lewis CE, Maschak-Carey BJ, Montez MG, Murillo A, Nathan DM, Patricio J, Peters A, Pi-Sunyer X, Pownall H, Reboussin D, Regensteiner JG, Rickman AD, Ryan DH, Safford M, Wadden TA, Wagenknecht LE, West DS, Williamson DF, Yanovski SZ: Cardiovascular effects of intensive lifestyle intervention in type 2 diabetes. N Engl J Med 2013;369:145-154

14. Perseghin G, Price TB, Petersen KF, Roden M, Cline GW, Gerow K, Rothman DL, Shulman GI: Increased glucose transport-phosphorylation and muscle glycogen synthesis after exercise training in insulin-resistant subjects. N Engl J Med 1996;335:1357-1362
15. Auerbach P, Nordby P, Bendtsen LQ, Mehlsen JL, Basnet SK, Vestergaard H, Ploug T, Stallknecht B: Differential effects of endurance training and weight loss on plasma adiponectin multimers and adipose tissue macrophages in younger, moderately overweight men. American journal of physiology Regulatory, integrative and comparative physiology 2013;305:R490-498
16. Schubert MM, Sabapathy S, Leveritt M, Desbrow B: Acute exercise and hormones related to appetite regulation: a meta-analysis. Sports medicine (Auckland, NZ) 2014;44:387-403
17. Liang CP, Han S, Li G, Tabas I, Tall AR: Impaired MEK signaling and SERCA expression promote ER stress and apoptosis in insulin-resistant macrophages and are reversed by exenatide treatment. Diabetes 2012;61:2609-2620
18. Kim Chung le T, Hosaka T, Yoshida M, Harada N, Sakaue H, Sakai T, Nakaya Y: Exendin-4, a GLP-1 receptor agonist, directly induces adiponectin expression through protein kinase A pathway and prevents inflammatory adipokine expression. Biochem Biophys Res Commun 2009;390:613-618
19. Hadjiyanni I, Siminovitch KA, Danska JS, Drucker DJ: Glucagon-like peptide-1 receptor signalling selectively regulates murine lymphocyte proliferation and maintenance of peripheral regulatory T cells. Diabetologia 2010;53:730-740
20. Chaudhuri A, Ghanim H, Vora M, Sia CL, Korzeniewski K, Dhindsa S, Makdissi A, Dandona P: Exenatide exerts a potent antiinflammatory effect. J Clin Endocrinol Metab 2012;97:198-207
21. Hogan AE, Gaoatswe G, Lynch L, Corrigan MA, Woods C, O'Connell J, O'Shea D: Glucagon-like peptide 1 analogue therapy directly modulates innate immune-mediated inflammation in individuals with type 2 diabetes mellitus. Diabetologia 2014;57:781-784
22. Parkner T, Sorensen LP, Nielsen AR, Fischer CP, Bibby BM, Nielsen S, Pedersen BK, Moller HJ: Soluble CD163: a biomarker linking macrophages and insulin resistance. Diabetologia 2012;55:1856-1862
23. Moller HJ, Frikke-Schmidt R, Moestrup SK, Nordestgaard BG, Tybjaerg-Hansen A: Serum soluble CD163 predicts risk of type 2 diabetes in the general population. Clinical chemistry 2011;57:291-297
24. Seufert J, Gallwitz B: The extra-pancreatic effects of GLP-1 receptor agonists: a focus on the cardiovascular, gastrointestinal and central nervous systems. Diabetes Obes Metab 2014;16:673-688
25. Xie L, Jiang Y, Ouyang P, Chen J, Doan H, Herndon B, Sylvester JE, Zhang K, Molteni A, Reichle M, Zhang R, Haub MD, Baybutt RC, Wang W: Effects of dietary calorie restriction or exercise on the PI3K and Ras signaling pathways in the skin of mice. The Journal of biological chemistry 2007;282:28025-28035
26. Cheng S-M, Ho T-J, Yang A-L, Chen IJ, Kao C-L, Wu F-N, Lin JA, Kuo C-H, Ou H-C, Huang C-Y, Lee S-D: Exercise training enhances cardiac IGFI-R/PI3K/Akt and Bcl-2 family associated pro-survival pathways in streptozotocin-induced diabetic rats. International Journal of Cardiology 167:478-485
27. Tousoulis D, Antoniades C, Stefanadis C: Evaluating endothelial function in humans: a guide to invasive and non-invasive techniques. Heart 2005;91:553-558
28. Lee R, Margaritis M, Channon KM, Antoniades C: Evaluating oxidative stress in human cardiovascular disease: methodological aspects and considerations. Curr Med Chem 2012;19:2504-2520
29. Torekov SS, Holst JJ, Ehlers MR: Dose response of continuous subcutaneous infusion of recombinant glucagon-like peptide-1 in combination with metformin and sulphonylurea over 12 weeks in patients with type 2 diabetes mellitus. Diabetes ObesMetab 2013;

30. Torekov SS, Madsbad S, Holst JJ: Obesity - an indication for GLP-1 treatment? Obesity pathophysiology and GLP-1 treatment potential. ObesRev 2011;12:593-601
31. Torekov SS, Kipnes MS, Harley RE, Holst JJ, Ehlers MR: Dose response of subcutaneous GLP-1 infusion in patients with type 2 diabetes. Diabetes ObesMetab 2011;13:639-643
32. Madsbad S, Kielgast U, Asmar M, Deacon CF, Torekov SS, Holst JJ: An overview of once-weekly glucagon-like peptide-1 receptor agonists--available efficacy and safety data and perspectives for the future. Diabetes ObesMetab 2011;13:394-407
33. Iepsen EW, Torekov SS, Holst JJ: Therapies for inter-relating diabetes and obesity - GLP-1 and obesity. Expert opinion on pharmacotherapy 2014:1-14
34. Asmar M, Simonsen L, Madsbad S, Stallknecht B, Holst JJ, Bulow J: Glucose-dependent insulinotropic polypeptide may enhance fatty acid re-esterification in subcutaneous abdominal adipose tissue in lean humans. Diabetes 2010;59:2160-2163
35.Eva W. Iepsen, Julie Lundgren, Sten Madsbad, DMSci, Jens J. Holst, Signe S. Torekov Successful weight loss maintenance includes long-lasting increases in the appetite inhibiting hormones GLP-1 and PYY_{3-36} American Diabetes Association (abstract), Diabetes 2015
36. Bodil J. Christensen, Eva W. Iepsen, Julie Lundgren, Sten Madsbad, Jens J. Holst, Signe S Torekov Sustained weight loss is associated with beneficial health outcomes and behavioural, affective and contextual factors are important psycho-social mediators in successful weight loss maintenance. European Congress of Obesity (abstract) 2015.
37. Fjeldborg K, Christiansen T, Bennetzen M, Moller J, Pedersen SB, Richelsen B. The macrophage-specific serum marker, soluble CD163, is increased in obesity and reduced after dietary-induced weight loss. Obesity (Silver Spring) 2013 Dec;21(12):2437-43.
38. Kaati G, Bygren LO, Pembrey M, Sjostrom M. Transgenerational response to nutrition, early life circumstances and longevity. Eur J Hum Genet 2007 Jul;15(7):784-90.
39. Pembrey ME, Bygren LO, Kaati G, Edvinsson S, Northstone K, Sjostrom M, et al. Sex-specific, male-line transgenerational responses in humans. Eur J Hum Genet 2006 Feb;14(2):159-66.
40. Donkin I, Versteyhe S, Ingerslev LR, Qian K, Mechta M, Nordkap L, et al. Obesity and Bariatric Surgery Drive Epigenetic Variation of Spermatozoa in Humans. Cell Metab 2015 Dec 6.
41. Crane JD, Mottillo EP, Farncombe TH, Morrison KM, Steinberg GR. A standardized infrared imaging technique that specifically detects UCP1-mediated thermogenesis in vivo. Mol Metab 2014 Jul;3(4):490-4.
42. Lee P, Linderman JD, Smith S, Brychta RJ, Wang J, Idelson C, et al. Irisin and FGF21 are cold-induced endocrine activators of brown fat function in humans. Cell Metab 2014 Feb 4;19(2):302-9.
43. Finlayson G, King N, Blundell J. The role of implicit wanting in relation to explicit liking and wanting for food: implications for appetite control. Appetite 2008 Jan;50(1):120-7
44. Flint A, Raben A, Blundell JE, Astrup A. Reproducibility, power and validity of visual analogue scales in assessment of appetite sensations in single test meal studies. Int J Obes Relat Metab Disord 2000 Jan;24(1):38-48.

Summary of changes from original protocol to final protocol

Amendment number (issue date)	Timing of change (before/after FPFV 12-Sep-2016)	Rationale and key changes
1 (08-Dec-2016)	After	• Increasing the upper body mass index limit (inclusion criteria) from 40 kg/m^2 to 43 kg/m^2 to increase the possibility that the aimed number of study participants will be reached within the planned trial period.<br>• Adding an exclusion criteria (upper limit of max 2 hours of regular exercise training at vigorous intensity) to ensure that the physical activity levels of the study participants are comparable.<br>• Specifying methodological details on the exercise intervention (e.g. target intensity)<br>• Specifying methodological details and add ethical considerations of the genomic examinations from blood and semen samples (cf. new Danish regulations on this area from 2016), including which genomic tests are performed and why.

2<br><br>(08-10-2018)	After	• Adding a post-treatment follow-up visit to investigate anthropometric and metabolic outcomes one year after completion of the trial.<br>• Prolonging the trial period from August 2016-August 2021 to August 2016-August 2023 due to addition of the post-treatment follow-up visit.<br>• Increasing the number of included study participants from 180 to 200 to increase the possibility that the aimed number of study participants of minimum 30 persons in each treatment arm complete the trial within the planned trial period.<br>• Removing statement of two planned exploratory examinations (MR scan for hepatic fat content and activation of brown fat tissue) as these were not performed.
3<br><br>(20-11-2019)	After	• Increasing the total number of included study participants from 200 to 222 because some of the individuals who had been

		given a study ID number at pre-screening never attended the first day of the run-in phase and thus did never initiate the trial (e.g. due to waiting time from pre-screening to the first day of run-in).<br>• Specifying details of the definition of the intention-to-treat analysis set to include all randomized participants<br>• Specifying details on the statistical method and refer to the statistical analysis plan

FPFV, first patient first visit.

Statistical Analysis Plan for clinical outcomes in the S-LiTE study

Section 1: Administrative information

Title: **Synergy effect of the appetite hormone GLP-1 (LiragluTide) and Exercise on maintenance of weight loss and health after a low calorie diet – the *S-LiTE* randomized trial**

Publication date: 27 November 2019
SAP version: 1
Protocol version: 10
The Universal Trial Number (UTN): U1111-1173-3104
EudraCT no: 2015-005585-32
ClinicalTrials.gov Identifier: NCT04122716

This document is a supplement to the S-LiTE study protocol (1) and contains the statistical analysis plan for the article with the tentative title "A randomized controlled trial of the combined effects of the GLP-1 receptor agonist liraglutide and exercise on maintenance of weight loss, body composition and health after a very low-calorie diet" This document follows the guidelines for content of statistical analysis plans in clinical trials (2).

Scientific board
Signe Sørensen Torekov, Professor MSO
Sten Madsbad, Professor, DMSci
Bente Stallknecht, Professor, DMSci
Jens Juul Holst, Professor, DMSci
Julie Rehné Lundgren, MD, PhD Student
Charlotte Janus, MSC, PhD student
Christian Rimer Juhl, MD, PhD student
Simon Birk Kjær Jensen, MSc

Statistical advisor
Martin Bæk Blond, PhD, postdoc

Sponsor-Investigator
Signe Sørensen Torekov, Professor MSO
University of Copenhagen
Faculty of Health Sciences
Department of Biomedical Sciences
Blegdamsvej 3B
DK-2200 Copenhagen N
Denmark
TEL +45 35327509/ +45 22983827
torekov@sund.ku.dk

Signature page

To be signed by persons writing the Statistical Analysis Plan (SAP), senior statistician responsible, contributors to the SAP, principal investigator and co-investigators.

Title: **<u>S</u>ynergy effect of the appetite hormone GLP-1 (*<u>Li</u>*raglu*<u>T</u>*ide) and <u>E</u>xercise on maintenance of weight loss and health after a low calorie diet – the *S-LiTE* randomized trial**

ClinicalTrials.gov Identifier: NCT04122716
EudraCT no: 2015-005585-32

I hereby declare that I have reviewed and approved the Statistical Analysis Plan

Name	Title	Role	Signature	Date
Signe Sørensen Torekov[1,2]	Professor MSO, Phd	Person writing the SAP and sponsor-investigator	[illegible]	20/11-19
Simon Birk Kjær Jensen[1,2]	MSc	Person writing the SAP Co-investigator	Simon Jensen	20/11-19
Martin Bæk Blond[3]	PhD	Senior statistician responsible	[illegible]	22/11-19
Charlotte Janus[1,2]	MSc, PhD-student	Contributor to SAP Co-investigator	[illegible]	20/11-19
Julie Rehné Lundgren[1,2]	MD, PhD-student	Contributor to SAP Co-investigator	[illegible]	21/11-19
Sten Madsbad[4]	Professor, DMSci	Principal investigator	[illegible]	20/11-19
Christian Rimer Juhl[1,2]	MD, PhD-student	Co-investigator	[illegible]	20/11-19
Bente Merete Stallknecht[1]	Professor, DMSci	Co-investigator	[illegible]	22/11-19
Jens Juul Holst[1,2]	Professor, DMSci	Co-investigator	[illegible]	22/11-2019

<u>Affiliations</u>
[1]Department of Biomedical Sciences, University of Copenhagen, Copenhagen, Denmark
[2]Novo Nordisk Foundation Center for Basic Metabolic Research, University of Copenhagen, Copenhagen, Denmark
[3]Steno Diabetes Center Copenhagen, Gentofte, Denmark
[4]Department of Endocrinology, Hvidovre Hospital, Hvidovre, Denmark

Contents

Section 1: Administrative information 1
Signature page 2
Section 2: Introduction 4
7 Background and Rationale (adapted from published protocol(1)) 4
8 Objectives 5
Section 3: Study Methods 6
9 Trial design (from published protocol(1)) 6
10 Randomization 7
11 Sample size 7
12 Framework 7
13 Statistical interim analyses and stopping guidance 7
14 Timing of final analyses 7
15 Timing of outcome assessments 7
Section 4: Statistical Principles 8
16-18 Confidence intervals and P values 8
19-20 Adherence and protocol deviations and Analysis populations 8
Section 5: Trial Population 9
21 Screening data 9
22 Eligibility 9
23 Recruitment 10
24 Withdrawal/follow-up 10
25 Baseline participant characteristics 10
Section 6: Analysis 11
26 Outcome definitions 11
27 Analysis methods 13
28 Missing data 13
29 Additional analyses 14
30 Harms 14
31 Statistical software 14
References 14

Section 2: Introduction

7 Background and Rationale (adapted from published protocol(1))

Obesity is associated with increased risk of developing cardiovascular disease and type 2 diabetes (T2D), along with increased risk of all-cause mortality (3,4). Obesity management guidelines recommends weight loss of more than 5 % of initial body weight to improve cardiometabolic risk factors, with greater weight loss producing greater benefits (5,6). However, weight regain reverse these health benefits (7,8). Furthermore, intentional weight loss is typically followed by a 30 to 50 % regain of lost weight within the first year (9–11). The main biological reasons for the rapid weight regain may be that weight loss causes a decrease in total energy expenditure to a degree that is greater than predicted from the decrease in fat and lean mass (12,13) in combination with increased appetite in the weight-reduced state (14,15).

Increasing energy expenditure by increasing physical activity is the first-line lifestyle modification in the treatment of obesity along with reducing food intake. For exercise interventions targeting general public health recommendations (at least 150 min/week of moderate intensity aerobic exercise), the associated weight loss is often modest (0-3 %) without concomitant calorie restriction (16–18). However, with exercise almost exclusively fat mass is lost whereas lean mass is close to unchanged (19–22) thereby improving body composition. Furthermore, independent of weight loss, exercise improves glycemic control, low grade inflammatory profile and cardiorespiratory fitness in individuals with overweight and obesity (21,23–25).

Glucagon-like peptide-1 (GLP-1) is an incretin hormone secreted from L-cells in the gut after food intake. GLP-1 stimulates glucose-dependent insulin secretion thereby lowering blood glucose and reduces appetite and thereby food intake (26,27). Treatment for 56 weeks with the GLP-1 receptor agonist (GLP-1 RA), liraglutide (3.0 mg), as an adjunct to regular diet and physical activity recommendations has been shown to improve glycemic control and induce moderate weight loss of 4.0 % in patients with T2D (28) and 5.4 % in non-diabetic individuals with overweight or obesity (29) compared to placebo. In addition, liraglutide has been shown to maintain a diet-induced weight loss over 56 weeks (30) and maintain very low-calorie diet-induced improvements of fasting plasma glucose and triglycerides over 52 weeks of weight loss maintenance superior to similar diet-induced weight loss maintenance in obese individuals (15). Weight loss with liraglutide is a result of both reduced fat and lean mass (31,32).

Obesity is associated with chronic low-grade inflammation (33,34) which is linked to the development of atherosclerosis and insulin resistance (35–37). Physically active individuals have lower inflammatory biomarker concentrations than their inactive counterparts (24), possibly explained by anti-inflammatory effects of an acute bout of exercise (38) and lower levels of visceral adipose tissue (39). GLP-1 has also emerged as an immunomodulatory agent, as illustrated by GLP-1 analogue administration exerting anti-inflammatory actions in various cells, including endothelial cells, adipocytes, peripheral blood mononuclear cells, and in plasma (40–44). Notably, in patients with T2D and high cardiovascular disease risk, GLP-1 RAs reduced the rate of occurrence of first major cardiovascular event (45,46). Thus, both physical activity and GLP-1 RA treatment seem to facilitate weight loss maintenance, improve metabolic health, and reduce systemic inflammation. However, diet-induced weight loss decreases energy expenditure and increases appetite.

We hypothesize that the combination of physical activity and liraglutide treatment improves weight loss maintenance, body composition and immunometabolic health since the decreased energy expenditure is targeted with exercise and the increased appetite with liraglutide.

8 Objectives

The overall objectives of this study are to investigate the maintenance of weight loss, body composition and immunometabolic health outcomes over 52 weeks with liraglutide treatment, physical exercise, and the combination in individuals with obesity after a very low-calorie diet.
The hypothesis hierarchy was not clearly specified in the protocol and we hereby define it as described below.

Hypothesis hierarchy for the primary outcome:

1. We hypothesize that the combined treatment (exercise+liraglutide) is superior to placebo for maintaining total body weight loss. Superiority is claimed if the estimated difference between the changes in total body weight for the two groups (delta for exercise+liraglutide from V1 to V3 – delta for placebo from V1 to V3) favorizes the exercise+liraglutide group and that the lower limit of the CI95% for the estimated difference exceeds 0.

2. We hypothesize that liraglutide is superior to placebo for maintaining total body weight loss. Superiority is claimed if the estimated difference between the changes in total body weight for the two groups (delta for liraglutide from V1 to V3 – delta for placebo from V1 to V3) favorizes the liraglutide group and that the lower limit of the CI95% for the estimated difference exceeds 0.

3. We hypothesize that the combined treatment (exercise+liraglutide) is non-inferior to liraglutide for maintaining total body weight loss.

4. We hypothesize that exercise is non-inferior to placebo for maintaining total body weight loss.

5. We hypothesize that the combined treatment (exercise+liraglutide) is superior to exercise for maintaining total body weight loss.

Secondary outcome:

6. We hypothesize that the combined treatment (liraglutide+exercise) is superior to liraglutide for body composition (total body fat percentage). Superiority is claimed if the estimated difference between the changes in total body fat percentage for the two groups (delta for liraglutide+exercise from V1 to V3 – delta for liraglutide from V1 to V3) favorizes the liraglutide+exercise group and that the lower limit of the CI95% for the estimated difference exceeds 0.

7. We hypothesize that exercise is superior to placebo for body composition (total body fat percentage). Superiority is claimed if the estimated difference between the changes in total body fat percentage for the two groups (delta for exercise from V1 to V3 – delta for placebo from V1 to V3) favorizes the exercise group and that the lower limit of the CI95% for the estimated difference exceeds 0.

8. We hypothesize that the combined treatment (exercise+liraglutide) is non-inferior to exercise for body composition (total body fat percentage).

Section 3: Study Methods

9 Trial design (from published protocol(1))

The S-LiTE trial (acronym for 'Synergy effect of the appetite hormone GLP-1 (LiragluTide) and Exercise on maintenance of weight loss and health after a low calorie diet') is an investigator-initiated, randomized, placebo-controlled, parallel group trial. The trial is triple-blinded with regards to study medication but not exercise intervention. The trial is registered at the European Clinical Trials Database (EudraCT Nr.: 2015-005585-32).

Description of Interventions

Diet-induced weight loss: Initially, all participants will undergo eight weeks with a very low-calorie diet (VLCD) (Cambridge Weight Plan, 800 kcal/day) with the objective to lose at least 5 % of body weight. Although benefits are evident already at modest weight loss of 2-3 % (e.g. triglycerides and HbA1c) (6), a ≥ 5 % cut-off after 8 weeks is chosen because fast weight losers have obtained a greater weight reduction and long-term maintenance, and were not more susceptible to weight regain than gradual weight losers (47). Participants who have lost at least 5 % of body weight after the eight-week weight loss phase will be randomized to one of the four study groups: 52 weeks of treatment with 1) placebo, 2) exercise + placebo, 3) liraglutide, or 4) exercise + liraglutide.

Liraglutide or placebo

The GLP-1 RA, liraglutide (3.0 mg), or placebo will be administrated once daily as subcutaneous injections in the abdomen or thigh. The starting dose is 0.6 mg with weekly increments of 0.6 mg until 3.0 mg is achieved. Participants who do not tolerate the 3.0 mg dose may in special circumstances stay at a lower dose (2.4 mg). However, the aim is to reach 3.0 mg for all study participants.

Physical exercise

The exercise intervention follows WHO's global recommendations on physical activity for health of 150 minutes of moderate-intensity aerobic physical activity throughout the week or 75 minutes of vigorous-intensity aerobic physical activity throughout the week or an equivalent combination of moderate- and vigorous-intensity activity (48). The intervention aims for four sessions per week. Two sessions per week will be performed under supervision of the study staff and two sessions will be performed individually. Supervised sessions will consist of structured exercise for a duration of 45 min. Of this, 30 min will be interval-based spinning and 15 min will be circuit training focusing on large muscle groups. Individual exercise sessions will include aerobic exercise such as cycling, rowing, or elliptical training as well as brisk walking or cycling to work. Participants randomized to an exercise group will undergo a 6-week ramp-up phase with exercise before exercising four times per week from week 7 to 52. Participants not randomized to exercise will be instructed to maintain habitual physical activity according to level before entering the trial.

Liraglutide and physical exercise

Combination of the two interventions described above.

Study visits

Identical test days will take place before the initial weight loss phase (V0, week -8), after initial weight loss (V1, week 0) and after 52 weeks of treatment (V3). For details on test days, see study protocol (1).

Weight consultations will be performed weekly during initial VLCD phase and at week 1, 2, 3, 4, 9, 13, 17, 22, 26, 32, 39 and 46. Fasting blood samples will be collected at week 4, 13, 26 and 39.

Finally, participants will be invited to complete a post-trial unsupervised follow-up visit (V4) 1 year after intervention completion.

10 Randomization

After the initial eight-week VLCD phase, participants will be randomized after the test day (V1) to one of the four study groups in a 1:1:1:1 ratio in accordance with a subject randomization list (SRL) provided by Novo Nordisk. An un-blinded study nurse (not otherwise associated with the trial) will allocate study participants according to the SRL. Randomization will be stratified by sex (male/female) and age (below/above 40 years).

11 Sample size

<u>Sample size calculation for total body weight:</u>

Sample size for primary endpoint was calculated in relation to total body weight. Based on previous weight loss studies with liraglutide (15,49), we estimate the response within each treatment group to be normally distributed with a standard deviation of 5.5 kg. Thus, with 30 participants completing each study arm we will be able to detect a difference in delta of 4 kg between groups with a power of 0.8, assuming a two-sided α-level of 0.05. We will need 34 completers in each arm in order to attain a statistical power of 0.85 and 40 completers in each arm to attain a statistical power of 0.90. In our previous study, 10 % of participants who entered the initial VLCD phase did not complete this phase (15). With 222 recruited study participants and an expected dropout rate of 25 % after randomization, we expect to have at least 30 participants from each study arm to complete the trial.

<u>Sample size for total body fat percentage</u>

Sample size for secondary endpoint body composition was calculated in relation to total body fat percentage. Based on previous exercise trials (20,50–52) the response within each treatment group was estimated to have a standard deviation of 2.1%. Thus, with 32 participants completing each study arm we will be able to detect a difference in delta body fat percentage of 1.5% between groups with a power of 0.8, assuming a two-sided α-level of 0.05. We will need 36 completers to attain a statistical power of 0.85. Sample size calculation for fat percentage was performed in relation to writing the SAP; after the intervention was started but prior to the termination of the intervention.

12 Framework

See point 8.

13 Statistical interim analyses and stopping guidance

No interim analyses were planned and no guidelines for terminating the trial early was made.

14 Timing of final analyses

Results from V1 to V3 will be analyzed when the final participant completes the trial. Analyses including V4 will be performed when the final participant has completed V4.

15 Timing of outcome assessments

Body weight is measured at week -8 (V0), -7, -6, -5, -4, -3, -2, -1, 0 (V1), 1, 2, 4, 9, 13, 17, 22, 26, 39, 46, 52 (V3) and 104 (V4).

Body composition is measured at V0, V1, V3 and V4.
See point 26 for timing of descriptive/explorative outcome assessments.

Section 4: Statistical Principles

16-18 Confidence intervals and P values

P-values and 95% confidence intervals will be presented for comparisons (between and within group) and will not be adjusted for multiplicity. 95% confidence intervals will be presented for estimated levels and will not be adjusted for multiplicity. A hierarchical testing procedure will be used to control the type 1 error for tests with predefined hypotheses (see point 8); all subsequent tests will be regarded as descriptive/explorative if a test fails to confirm a given hypothesis. All non-hypothesis-based tests are per definition of a descriptive/exploratory nature. Statistical significance will be claimed if the null hypothesis is rejected at the alpha level of 0.05 (two-sided), i.e. the P-value of the null hypothesis test is $\leq$ 0.05. 95% confidence intervals will be required to support the tested hypothesis for hypothesis-based tests (see 8), and for exploratory/descriptive test on an absolute or relative scale not include 0 or 1 respectively.

19-20 Adherence and protocol deviations and Analysis populations

<u>Intention-to-treat (ITT) analysis set:</u>
All participants analyzed as randomized.

<u>Per Protocol (PP) analysis set:</u>
All participants who complete the 52-week randomized treatment period with sufficient compliance to study medication and/or exercise protocol as defined by:
<u>Study medication:</u> Having administered 2.4 or 3.0 mg subcutaneous (sc) liraglutide/placebo for at least 75% of the intervention period (measured by self-reporting during the 12 visits from week 1 to 52 after up-titration). Compliance to study medication will be summarized as number and percentage of participants equal to or above 75 %.
<u>Exercise:</u> Sports watches with heart rate monitors will be worn during all exercise sessions. For all participants in an exercise group, we will report duration of exercise (minutes per week), mean intensity during exercise (percentage of maximum heart rate) and exercise frequency (times per week). Exercise duration, intensity and frequency will be summarized as median plus 25th and 75th percentiles. Heart rate will be measured with a frequency of 1 Hz during all exercise. Relative exercise intensity for each heart rate measurement will be classified based on percentage of maximum heart rate (determined during an maximal incremental cycle ergometer test) in accordance with ACSM's position stand on prescribing exercise(53), i.e. very light intensity (<57% of HR_{max}), light intensity (57-63% of HR_{max}), moderate intensity (64-76% HR_{max}), vigorous intensity (77-95% HR_{max}) and near-maximal to maximal intensity ($\geq$ 96% HR_{max}).
For exercise sessions where heart rate data is missing (e.g. due to forgotten watch for supervised exercise), the exercise duration will be noted and the participants average intensity and time at different intensity zones will be imputed. Exercise will be averaged for all weeks after the ramp-up phase and until end-of-trial test day and exercise compliance will be calculated as percentage of WHO's global recommendations on physical activity for health (48): *Adults aged 18–64 should do at least 150 minutes of moderate-intensity aerobic physical activity throughout the week or do at least 75 minutes of vigorous-intensity aerobic physical activity throughout the week or an equivalent combination of moderate- and vigorous-intensity*

activity. Thus, for example, one minute of moderate-intensity exercise will account for 1/150 and one minute of vigorous- or near-maximal to maximal intensity exercise will account for 1/75 of prescribed weekly exercise. Per protocol analysis set will be defined as meeting at least 75% of global recommendations.

For participants to be included in the per protocol analysis:
a) Liraglutide group: Having administered 2.4 or 3.0 mg sc liraglutide for at least 75% of the intervention period.
b) Placebo group: Having administered 2.4 or 3.0 mg sc placebo for at least 75% of the intervention period.
c) Exercise + placebo group: Having administered 2.4 or 3.0 mg sc placebo for at least 75% of the intervention period and having met at least 75% of global recommendations on physical activity.
d) Exercise + liraglutide group: Having administered 2.4 or 3.0 mg sc liraglutide for at least 75% of the intervention period and having met at least 75% of global recommendations on physical activity.

Section 5: Trial Population

21 Screening data

Screening data will not be reported.

22 Eligibility

Inclusion criteria:

- BMI > 32 and < 43 (kg/m^2)
- Age >18 and <65 years
- Safe contraceptive method

Exclusion criteria:

- Patients diagnosed with *known* serious chronic illness including type 1 or 2 diabetes (or a randomly measured fasting plasma glucose > 7 mmol/l)
- Angina pectoris, coronary heart disease, congestive heart failure (NYHA III-IV)
- Severe renal impairment (creatinine clearance (GFR) <30 mL/min)
- Severe hepatic impairment
- Inflammatory bowel disease
- Diabetic gastroparesis
- Cancer
- Chronic obstructive lung disease
- Psychiatric disease, a history of major depressive or other severe psychiatric disorders
- The use of medications that cause clinically significant weight gain or loss
- Previous bariatric surgery
- A history of idiopathic acute pancreatitis
- A family or personal history of multiple endocrine neoplasia type 2 or familial medullary thyroid carcinoma
- Osteoarthritis which is judged to be too severe to manage the exercise programme. As intended per study design the intervention will include a 5% weight loss prior to randomization, thus it is

expected that possible participants with mild form of osteoarthritis will be able to manage exercise prescriptions.

- Pregnancy, expecting pregnancy or breast feeding. If a study participant is in doubt whether she could be pregnant, a urine pregnancy test is performed. Females of childbearing potential who are not using adequate contraceptive methods (as required by local law or practice). Adequate contraception must be used throughout the study period and at least 65 hours after discontinuation of trial medication (65 hours corresponds to 5 times the half-life of liraglutide).
- Allergy to any of the ingredients/excipients of the study medication: liraglutide, disodium phosphate dihydrate, propylene glycol, phenol, hydrochloric acid, sodium hydroxide.
- Regular exercise training at high intensity (e.g. spinning) >2 hours per week.

23 Recruitment

The flow chart of the trial will follow the CONSORT guidelines and will include the number of participants who a) received oral information, b) were assessed for eligibility at prescreening, c) were included in the trial, d) attended first test day and initiated VLCD, e) were withdrawn or excluded from the VLCD phase, f) were randomized, g) were allocated to the four intervention groups, h) lost to follow-up, i) discontinued the intervention, and j) were analyzed in the end.

24 Withdrawal/follow-up

If a randomized participant withdraws, we will offer to perform all or some of the investigations planned at V3. The number/frequency of participants lost to follow-up (those not attending a weight measurement at V3) will be provided for each group and for each time point. If possible, the reasons for participants not completing the trial will be given. Summary of baseline levels for variables reported in the baseline table will be provided for completers and non-completers. Spaghetti plots will be used to visualize levels of the main outcome for completers and non-completers.

25 Baseline participant characteristics

The distribution of all outcomes included in baseline characteristics will be visually inspected using QQ-plots and histograms; those with a Gaussian distribution will be presented as means and standard deviations and those with a non-Gaussian distribution will be presented as medians plus 25th and 75th percentiles.

The following outcomes will be included in the baseline participant characteristics table for all participants combined at V0, all participants combined at V1 and participants divided by randomization group at V1:

- Number of participants (men/women)
- Age (years)
- Weight (kg)
- BMI (kg/m^2)
- Waist circumference (cm)
- Total fat mass (kg)
- Total fat free mass (kg)
- Body fat percentage (%)

- Systolic blood pressure (mmHg)
- Diastolic blood pressure (mmHg)
- HbA1c (mmol/mol)
- Fasting glucose (mmol/L)
- Fasting insulin (pmol/L)
- Fasting C-peptide (pmol/L)
- Glucose AUC (mmol/L*min)
- Insulin AUC (pmol/L*min)
- Cholesterol: total, LDL and HDL (mmol/L)
- Triglycerides (mmol/L)
- Peak oxygen consumption (ml/min)
- Peak oxygen consumption (ml/min/kg)
- HOMA-IR (fasting insulin (pmol/L) * fasting glucose (mmol/L) / 22.5)
- Matsuda index (10000/sqrt(fasting glucose * fasting insulin * mean glucose * mean insulin)
- SF-36 (scoring of eight health concepts ranging from 0-100: physical functioning, role limitations due to physical health problems, role limitations due to personal or emotional problems, energy/fatigue, emotional well-being, social functioning, pain, and general health perception)
- ALAT (U/L)
- Amylase (U/L)
- CRP (mg/L)

Section 6: Analysis

26 Outcome definitions

Primary outcome: Primary outcome is the change in total body weight (measured to the nearest 0.1 kg) from baseline (V1) to end-of-treatment (V3). A weight difference of 2.5-4.5 % body weight, obtained with liraglutide or semaglutide compared to placebo, have been associated with beneficial cardiovascular outcomes (45,46). A weight difference of 1-5% of body weight obtained with exercise has been associated with beneficial cardiovascular disease risk factors (17,18) and prevention of diabetes (54).
Thus, a difference in delta of 3-5% of total body weight has been defined as the minimal important difference. With a study population consisting of men and women (expected mean height of 170-174 cm based on previous lifestyle trials performed in Denmark (15,55,56) and a BMI range of 32-43 kg /m^2, a difference of 3-5% of total body weight will correspond to 4 kg.

Secondary outcome: Secondary outcome body composition is defined as change in total body fat percentage (measured to the nearest 0.01 % in fasted state with dual-energy X-ray absorptiometry (DXA) scans) from V1 to V3. In most exercise interventions with aerobic exercise, almost exclusively fat mass is lost whereas lean mass is close to unchanged (19–22). In weight loss studies with liraglutide, a combination of fat and lean mass is lost (31,32). The clinically relevant effect size for changes in fat percentage is not well described or investigated. However, based on the results of previous exercise interventions (20,57), a decrease in fat percentage equivalent of approximately 1.5 % seems to be physiologically relevant.

<u>Descriptive/explorative outcomes:</u>
Change from V1 to V3:

- Body composition (fat free mass (kg) and fat mass (kg))
- Circulating biomarkers of metabolic regulation to evaluate metabolic health (fasting and 3h AUC glucose (mmol/L and mmol/L*min) and fasting and 3h AUC insulin (pmol/L and pmol/L*min) for HOMA-IR and Matsuda index, HbA1c (mmol/mol), waist circumference (cm), systolic and diastolic blood pressure (mmHg), lipids (total cholesterol, HDL, LDL, and triglycerides (mmol/L))
- Safety (adverse events). Adverse events with an incident of ≥ 5 % in any treatment group and all serious adverse events will be reported by system organ class and preferred term for participants in all four treatment groups separately and for liraglutide pooled (exercise and non-exercise) and placebo pooled (exercise and non-exercise). Events will be reported as percentage of individuals experiencing an adverse event.
- Self-rated quality of life will be measured with the SF-36 questionnaire. Eight health concepts ranging from 0-100 will be scored: physical functioning, role limitations due to physical health problems, role limitations due to personal or emotional problems, energy/fatigue, emotional well-being, social functioning, pain, and general health perception.
- Peak oxygen uptake (absolute (ml/min) and relative (ml/min/kg))
- Stair test (S) and maximal isometric strenght test (N)
- Use of medication (n, frequency)
- Meal related appetite response
- Determination of daily physical activity and sleep
- Systemic markers of immune-metabolism and oxidation
- Endothelial function
- Immuno-metabolic changes in the subcutaneous adipose tissue
- Gene expression profile of circulating inflammatory cells
- Bone health
- Food preference questionnaire (LFPQ) and subjective appetite sensation
- Faecal bacterial composition
- Plasma metabolomics and proteomics
- Epigenetics of spermatozoa
- Questionnaires (TFEQ, IPAQ, G-SES, PSQI)

Outcomes measured at other time points:

- Body weight (week -7, -6, -5, -4, -3, -2, -1, 1, 2, 4, 9, 13, 17, 22, 26, 32, 39, 46 and V4)
- Number/proportion of participants that have reduced total body weight by ≥5%, ≥10% and ≥15% at V3 compared with V0
- Body composition (V0 and V4)
- Metabolic health (week 4, 13, 26, 39 and V4)
- Safety (adverse events) (week 1, 2, 3, 4, 9, 13, 17, 22, 26, 32, 39 and 46)
- Meal related appetite response (V0)
- Peak oxygen uptake, stair test and maximal isometric strenght test (V0)
- Systemic markers of immune-metabolism and oxidation (V0)

- Endothelial function (V0)
- Immuno-metabolic changes in the subcutaneous adipose tissue (V0 and week 26)
- Gene expression profile of circulating inflammatory cells (V0)
- Bone health (V0 and V4)
- Food preference questionnaire (LFPQ) (V0 and V4) and subjective appetite sensation (V0)
- Faecal bacterial composition (V0)
- Plasma metabolomics and proteomics (V0, week 4, 13, 26, 39 and V4)
- Epigenetics of spermatozoa (V0)
- Questionnaries (SF-36, TFEQ, IPAQ, G-SES, PSQI) (V0 and V4)
- Measurement of sleep and physical activity levels (V0, week 13, 26 and V4)

27 Analysis methods

Analyses of primary and secondary outcome (except for the pre-planned sensitivity analyses) will be performed based on the intention-to-treat (ITT) principle. All continuous endpoints/outcomes will be modelled using linear mixed effects models with the following fixed effects and interactions: Time (factorial), Treatment, Time (factorial)*Treatment, sex (female, male) and age group (<40 years, >40 years). The models will be specified with a restricted maximum likelihood estimation method, the Kenward-Roger degrees of freedom method, an unstructured covariance structure and a random intercept on participant level. Model fit will be evaluated using graphical methods and if necessary, outcomes will be log-transformed. Estimated mean differences (CI95%) for changes between groups (main study effects), conditional means (CI95%), and within group changes (CI95%) will be extracted from the model. For log-transformed outcomes the results will be back-transformed and be presented as the ratio between estimated relative changes (CI95%), estimated conditional geometric means (CI95%) and within group relative changes (CI95%), respectively. Between group differences in changes will be null-hypothesis tested and presented with P-values.
The following predefined sensitivity analyses will be performed for the primary and secondary outcome; an additional ITT analysis with adjustment for initial weight loss, an ITT analysis using multiple imputation to assess effects of missing data (see also 28), and finally an analysis of per protocol completers.

The analysis of the primary and secondary outcome will be performed blinded to group allocation by a researcher (Martin Bæk Blond) that have not been involved in the execution of the trial. The statistical and clinical/physiological implications of the results will be evaluated by the research team before un-blinding.

28 Missing data

The number/frequency of missing values for the primary and secondary outcome in each group at each time point will be provided. For the main analysis, the results will be based on likelihood inference and missing data will be assumed to be missing completely at random or missing at random. An additional analysis based on multiple imputation of missing values will be performed (see also 27). The dataset used for the imputations will include all observations for total body weight measured at or between V1-V3, and the auxiliary variables Sex (male or female) and Age (continuous, years). Under the assumption that weight changes in participants after loss to follow-up would resemble the development in the placebo groups rather than the development in the group to which they were originally randomized, the participants with

missing values at V3 will be pooled with the placebo group and using a Markov chain Monte Carlo method all missing values will be imputed, assuming a multivariate normal distribution for the data, to create 1000 new datasets. Subsequently, the imputed datasets will be analyzed using the same mixed linear model used for the main analysis and averaged estimates calculated. If the main outcome has been transformed to fit the statistical model used for the main analysis this transformation will be applied in the imputation procedure.

29 Additional analyses

Not relevant

30 Harms

Adverse events (defined in the trial protocol) with an incident of ≥ 5 % in any treatment group and all serious adverse events will be reported by system organ class and preferred term for participants in all four treatment groups separately and for liraglutide pooled (exercise and non-exercise) and placebo pooled (exercise and non-exercise). Events will be reported as the number of and percentage of individuals experiencing an adverse event. Rates of adverse events will not be compared by null-hypothesis testing.

31 Statistical software

R version 3.6.0 or newer version (The R Foundation for Statistical Computing, www.R-project.org) and SAS version 9.4 or newer version (SAS Institute, Cary, NC, USA).

References

1 Jensen SBK, Lundgren JR, Janus C, Juhl CR, Olsen LM, Rosenkilde M *et al.* Protocol for a randomised controlled trial of the combined effects of the GLP-1 receptor agonist liraglutide and exercise on maintenance of weight loss and health after a very low-calorie diet. *BMJ Open* 2019; **9**: e031431.

2 Gamble C, Krishan A, Stocken D, Lewis S, Juszczak E, Doré C *et al.* Guidelines for the content of statistical analysis plans in clinical trials. *JAMA - J Am Med Assoc* 2017; **318**: 2337–2343.

3 Akram D, Astrup A, Atinmo T, Boissi J, Bray G, Carroll K *et al.* Obesity: Preventing and managing the global epidemic. World Health Organization. *Tech Rep Ser* 2000.

4 Abdelaal M, le Roux CW, Docherty NG. Morbidity and mortality associated with obesity. *Ann Transl Med* 2017; **5**: 161–161.

5 Garvey WT, Mechanick JI, Brett EM, Garber AJ, Hurley DL, Jastreboff AM *et al.* American association of clinical endocrinologists and American college of endocrinology comprehensive clinical practice guidelines for medical care of patients with obesity. *Endocr Pract* 2016; **22**: 1–203.

6 Jensen MD, Ryan DH, Apovian CM, Ard JD, Comuzzie AG, Donato KA *et al.* 2013 AHA/ACC/TOS Guideline for the Management of Overweight and Obesity in Adults. *Circulation* 2014; **129**: S102–S138.

7 Kroeger CM, Hoddy KK, Varady KA. Impact of weight regain on metabolic disease risk: A review of human trials. J Obes. 2014; **2014**: 614519.

8 Thomas TR, Warner SO, Dellsperger KC, Hinton PS, Whaley-Connell AT, Rector RS *et al.* Exercise and the metabolic syndrome with weight regain. *J Appl Physiol* 2010; **109**: 3–10.

9 Anderson JW, Konz EC, Frederich RC, Wood CL. Long-term weight-loss maintenance: a meta-analysis of US studies. *Am J Clin Nutr* 2001; **74**: 579–584.

10 Barte JCM, Ter Bogt NCW, Bogers RP, Teixeira PJ, Blissmer B, Mori TA *et al.* Maintenance of weight loss after lifestyle interventions for overweight and obesity, a systematic review. Obes Rev. 2010; **11**: 899–906.

11 Curioni CC, Lourenc -O PM. Long-term weight loss after diet and exercise: a systematic review. *Int J Obes* 2005; **29**: 1168–1174.

12 Rosenbaum M, Leibel RL. Adaptive thermogenesis in humans. *Int J Obes* 2010; **34**: S47–S55.

13 Leibel RL, Rosenbaum M, Hirsch J. Changes in Energy Expenditure Resulting from Altered Body Weight. *N Engl J Med* 1995; **332**: 621–628.
14 Iepsen EW, Lundgren J, Holst JJ, Madsbad S, Torekov SS. Successful weight loss maintenance includes long-term increased meal responses of GLP-1 and PYY3-36. *Eur J Endocrinol* 2016; **174**: 775–784.
15 Iepsen EW, Lundgren J, Dirksen C, Jensen JE, Pedersen O, Hansen T *et al.* Treatment with a GLP-1 receptor agonist diminishes the decrease in free plasma leptin during maintenance of weight loss. *Int J Obes* 2015; **39**: 834–841.
16 Swift DL, Johannsen NM, Lavie CJ, Earnest CP, Church TS. The Role of Exercise and Physical Activity in Weight Loss and Maintenance. *Prog Cardiovasc Dis* 2014; **56**: 441–447.
17 Donnelly JE, Blair SN, Jakicic JM, Manore MM, Rankin JW, Smith BK. Appropriate physical activity intervention strategies for weight loss and prevention of weight regain for adults. *Med Sci Sports Exerc* 2009; **41**: 459–471.
18 Shaw K, Gennat H, O'Rourke P, Del Mar C. Exercise for overweight or obesity. *Cochrane Database Syst Rev* 2006. doi:10.1002/14651858.CD003817.pub3.www.cochranelibrary.com.
19 Donnelly JE, Jacobsen DJ, Snyder Heelan K, Seip R, Smith S. The effects of 18 months of intermittent vs continuous exercise on aerobic capacity, body weight and composition, and metabolic ®tness in previously sedentary, moderately obese females. 2000www.nature.com/ijo (accessed 12 Nov2019).
20 Willis LH, Slentz CA, Bateman LA, Shields AT, Piner LW, Bales CW *et al.* Effects of aerobic and/or resistance training on body mass and fat mass in overweight or obese adults. *J Appl Physiol* 2012; **113**: 1831–1837.
21 Nordby P, Auerbach PL, Rosenkilde M, Kristiansen L, Thomasen JR, Rygaard L *et al.* Endurance training per se increases metabolic health in young, moderately overweight men. *Obesity* 2012; **20**: 2202–2212.
22 Foster-Schubert KE, Alfano CM, Duggan CR, Xiao L, Campbell KL, Kong A *et al.* Effect of diet and exercise, alone or combined, on weight and body composition in overweight-to-obese postmenopausal women. *Obesity* 2012; **20**: 1628–1638.
23 Bruce CR, Thrush AB, Mertz VA, Bezaire V, Chabowski A, Heigenhauser GJF *et al.* Endurance training in obese humans improves glucose tolerance and mitochondrial fatty acid oxidation and alters muscle lipid content. *Am J Physiol Endocrinol Metab* 2010; **291**: 99–107.
24 Beavers KM, Brinkley TE, Nicklas BJ. Effects of Exercise Training on Chronic Inflammation. *Clin Chim Acta* 2010; **411**: 785–793.
25 Lavie CJ, Arena R, Swift DL, Johannsen NM, Sui X, Lee DC *et al.* Exercise and the cardiovascular system: Clinical science and cardiovascular outcomes. *Circ Res* 2015; **117**: 207–219.
26 Flint A, Raben A, Astrup A, Holst JJ. Glucagon-like peptide 1 promotes satiety and suppresses energy intake in humans. *J Clin Invest* 1998; **101**: 515–520.
27 Holst JJ, Deacon CF, Vilsbøll T, Krarup T, Madsbad S. Glucagon-like peptide-1, glucose homeostasis and diabetes. *Trends Mol Med* 2008; **14**: 161–168.
28 Davies MJ, Bergenstal R, Bode B, Kushner RF, Lewin A, Skjøth TV *et al.* Efficacy of liraglutide for weight loss among patients with type 2 diabetes: The SCALE diabetes randomized clinical trial. *JAMA - J Am Med Assoc* 2015; **314**: 687–699.
29 Pi-Sunyer X, Astrup A, Fujioka K, Greenway F, Halpern A, Krempf M *et al.* A Randomized, Controlled Trial of 3.0 mg of Liraglutide in Weight Management. *N Engl J Med* 2015; **373**: 11–22.
30 Wadden TA, Hollander P, Klein S, Niswender K, Woo V, Hale PM *et al.* Weight maintenance and additional weight loss with liraglutide after low-calorie-diet-induced weight loss: The SCALE Maintenance randomized study. *Int J Obes* 2013; **37**: 1443–1451.
31 Jendle J, Nauck MA, Matthews DR, Frid A, Hermansen K, Düring M *et al.* Weight loss with liraglutide, a once-daily human glucagon-like peptide-1 analogue for type 2 diabetes treatment as monotherapy or added to metformin, is primarily as a result of a reduction in fat tissue. *Diabetes, Obes Metab* 2009; **11**: 1163–1172.
32 Frøssing S, Nylander M, Chabanova E, Frystyk J, Holst JJ, Kistorp C *et al.* Effect of liraglutide on ectopic fat in polycystic ovary syndrome: A randomized clinical trial. *Diabetes, Obes Metab* 2018; **20**: 215–218.
33 Stępień M, Stępień A, Wlazeł RN, Paradowski M, Banach M, Rysz J. Obesity indices and inflammatory markers in obese non-diabetic normo- and hypertensive patients: A comparative pilot study. *Lipids Health Dis* 2014; **13**: 10–13.
34 Panagiotakos DB, Pitsavos C, Yannakoulia M, Chrysohoou C, Stefanadis C. The implication of obesity and central fat on markers of chronic inflammation: The ATTICA study. *Atherosclerosis* 2005; **183**: 308–315.
35 Libby P, Ridker PM, Maseri A. Inflammation and Atherosclerosis. *Circulation* 2002; **105**.
36 Duncan BB, Schmidt MI, Pankow JS, Ballantyne CM, Couper D, Vigo A *et al.* Low-grade systemic inflammation and the development of type 2 diabetes: The atherosclerosis risk in communities study. *Diabetes* 2003; **52**:

1799–1805.
37 Vozarova B, Weyer C, Lindsay RS, Pratley RE, Bogardus C, Antonio Tataranni P. High white blood cell count is associated with a worsening of insulin sensitivity and predicts the development of type 2 diabetes. *Diabetes* 2002; **51**: 455–461.
38 Starkie R, Ostrowski SR, Jauffred S, Febbraio M, Pedersen BK. Exercise and IL-6 infusion inhibit endotoxin-induced TNF-α production in humans. *FASEB J* 2003; **17**: 884–886.
39 Gleeson M, Bishop NC, Stensel DJ, Lindley MR, Mastana SS, Nimmo MA. The anti-inflammatory effects of exercise: Mechanisms and implications for the prevention and treatment of disease. *Nat Rev Immunol* 2011; **11**: 607–610.
40 Chaudhuri A, Ghanim H, Vora M, Sia CL, Korzeniewski K, Dhindsa S *et al.* Exenatide exerts a potent antiinflammatory effect. *J Clin Endocrinol Metab* 2012; **97**: 198–207.
41 Hogan AE, Gaoatswe G, Lynch L, Corrigan MA, Woods C, O'Connell J *et al.* Glucagon-like peptide 1 analogue therapy directly modulates innate immune-mediated inflammation in individuals with type 2 diabetes mellitus. *Diabetologia* 2014; : 781–784.
42 Garczorz W, Gallego-Colon E, Kosowska A, Kłych-Ratuszny A, Woźniak M, Marcol W *et al.* Exenatide exhibits anti-inflammatory properties and modulates endothelial response to tumor necrosis factor α-mediated activation. *Cardiovasc Ther* 2018; **36**. doi:10.1111/1755-5922.12317.
43 von Scholten BJ, Persson F, Rosenlund S, Eugen-Olsen J, Pielak T, Faber J *et al.* Effects of liraglutide on cardiovascular risk biomarkers in patients with type 2 diabetes and albuminuria: A sub-analysis of a randomized, placebo-controlled, double-blind, crossover trial. *Diabetes, Obes Metab* 2017; **19**: 901–905.
44 Lee YS, Park MS, Choung JS, Kim SS, Oh HH, Choi CS *et al.* Glucagon-like peptide-1 inhibits adipose tissue macrophage infiltration and inflammation in an obese mouse model of diabetes. *Diabetologia* 2012; **55**: 2456–2468.
45 Marso SP, Daniels GH, Brown-Frandsen K, Kristensen P, Mann JFE, Nauck MA *et al.* Liraglutide and Cardiovascular Outcomes in Type 2 Diabetes. *N Engl J Med* 2016; **375**: 311–322.
46 Marso SP, Bain SC, Consoli A, Eliaschewitz FG, Jodar E, Leiter LA *et al.* Semaglutide and cardiovascular outcomes in patients with type 2 diabetes. *N Engl J Med* 2016; **375**: 1834–1844.
47 Nackers LM, Ross KM, Perri MG. The association between rate of initial weight loss and long-term success in obesity treatment: Does slow and steady win the race? *Int J Behav Med* 2010; **17**: 161–167.
48 World Health Organization. Physical Activity and Adults - Recommended levels of physical activity for adults aged 18 - 64 years. https://www.who.int/dietphysicalactivity/factsheet_adults/ (accessed 8 Apr2019).
49 Astrup A, Rössner S, Van Gaal L, Rissanen A, Niskanen L, Al Hakim M *et al.* Effects of liraglutide in the treatment of obesity: a randomised, double-blind, placebo-controlled study. *Lancet* 2009; **374**: 1606–1616.
50 Mensberg P, Nyby S, Jørgensen PG, Storgaard H, Jensen MT, Sivertsen J *et al.* Near-normalization of glycaemic control with glucagon-like peptide-1 receptor agonist treatment combined with exercise in patients with type 2 diabetes. *Diabetes, Obes Metab* 2017; **19**: 172–180.
51 Skrypnik D, Bogdański P, Mądry E, Karolkiewicz J, Ratajczak M, Kryściak J *et al.* Effects of Endurance and Endurance Strength Training on Body Composition and Physical Capacity in Women with Abdominal Obesity. *Obes Facts* 2015; **8**: 175–187.
52 Slentz CA, Duscha BD, Johnson JL, Ketchum K, Aiken LB, Samsa GP *et al.* Effects of the Amount of Exercise on Body Weight, Body Composition, and Measures of Central Obesity: STRRIDE - A Randomized Controlled Study. *Arch Intern Med* 2004; **164**: 31–39.
53 Garber CE, Blissmer B, Deschenes MR, Franklin BA, Lamonte MJ, Lee IM *et al.* Quantity and quality of exercise for developing and maintaining cardiorespiratory, musculoskeletal, and neuromotor fitness in apparently healthy adults: Guidance for prescribing exercise. *Med Sci Sports Exerc* 2011; **43**: 1334–1359.
54 Knowler WC, Barrett-Connor E, Fowler SE, Hamman RF, Lachin JM, Walker EA *et al.* Reduction in the incidence of type 2 diabetes with lifestyle intervention or metformin. *N Engl J Med* 2002; **346**: 393–403.
55 Blond MB, Rosenkilde M, Gram AS, Tindborg M, Christensen AN, Quist JS *et al.* How does 6 months of active bike commuting or leisure-time exercise affect insulin sensitivity, cardiorespiratory fitness and intra-abdominal fat? A randomised controlled trial in individuals with overweight and obesity. *Br J Sports Med* 2019; **53**: 1183–1192.
56 Iepsen EW, Zhang J, Thomsen HS, Hansen EL, Hollensted M, Madsbad S *et al.* Patients with Obesity Caused by Melanocortin-4 Receptor Mutations Can Be Treated with a Glucagon-like Peptide-1 Receptor Agonist. *Cell Metab* 2018; **28**: 23-32.e3.
57 Sigal RJ, Kenny GP, Boulé NG, Wells GA, Prud'homme D, Fortier M *et al.* Effects of aerobic training, resistance

training, or both on glycemic control in type 2 diabetes: A randomized trial. *Ann Intern Med* 2007; **147**: 357–369.

Statistical Analysis Plan for clinical outcomes in the S-LiTE study

Section 1: Administrative information

Title: **_S_ynergy effect of the appetite hormone GLP-1 (_Li_raglu_T_ide) and _E_xercise on maintenance of weight loss and health after a low calorie diet – the *S-LiTE* randomized trial**

Publication date: 4 March 2020
SAP version: 2
Protocol version: 10
The Universal Trial Number (UTN): U1111-1173-3104
EudraCT no: 2015-005585-32
ClinicalTrials.gov Identifier: NCT04122716

This document is a supplement to the S-LiTE study protocol (1) and contains the statistical analysis plan for the article with the tentative title "A randomized controlled trial of the combined effects of the GLP-1 receptor agonist liraglutide and exercise on maintenance of weight loss, body composition and health after a very low-calorie diet" This document follows the guidelines for content of statistical analysis plans in clinical trials (2).

Scientific board
Signe Sørensen Torekov, Professor MSO
Sten Madsbad, Professor, DMSci
Bente Stallknecht, Professor, DMSci
Jens Juul Holst, Professor, DMSci
Julie Rehné Lundgren, MD, PhD Student
Charlotte Janus, MSC, PhD student
Christian Rimer Juhl, MD, PhD student
Simon Birk Kjær Jensen, MSc

Statistical advisor
Martin Bæk Blond, PhD, postdoc

Sponsor-Investigator
Signe Sørensen Torekov, Professor MSO
University of Copenhagen
Faculty of Health Sciences
Department of Biomedical Sciences
Blegdamsvej 3B
DK-2200 Copenhagen N
Denmark
TEL +45 35327509/ +45 22983827
torekov@sund.ku.dk

SAP revisions

Changes from version 1 to 2

1. Item 8 (Page 6-7): The minimally important differences (physiologically relevant changes) for the primary and secondary outcomes (which was defined in item 26) have now also been added to the hypotheses for clarity. In the hypotheses section of SAP version 1 it was stated that superiority would be claimed if the changes to an outcome favorised the given group (see individual hypotheses on page 6-7) and the lower limit of the CI95% for the estimated difference did not *exceed* 0. The latter part has been corrected to "does not *include* 0" to reflect that a reduction is favorable as stated in the hypothesis.
2. Item 27 (Page 14): The model specification for the model used to assess changes to the primary and secondary outcomes was changed from a random intercept model to a repeated measures model in order to avoid the risk of falsely narrow confidence intervals and low P-values produced by the assumption of constant variance and covariance across repeated measurements.

Signature page

To be signed by persons writing the Statistical Analysis Plan (SAP), senior statistician responsible, contributors to the SAP, principal investigator and co-investigators.

Title: **<u>S</u>ynergy effect of the appetite hormone GLP-1 (*<u>Li</u>*raglu*<u>T</u>*ide) and <u>E</u>xercise on maintenance of weight loss and health after a low calorie diet – the *S-LiTE* randomized trial**

ClinicalTrials.gov Identifier: NCT04122716
EudraCT no: 2015-005585-32

Name	Title	Role	Signature	Date
Signe Sørensen Torekov[1,2]	Professor, Phd	Person writing the SAP and sponsor		28.2.20
Simon Birk Kjær Jensen[1,2]	MSc	Person writing the SAP		28/2-20
Martin Bæk Blond[3]	MD, PhD, postdoc	Senior statistician responsible		03/03-20
Charlotte Janus[1,2]	PhD-student	Contributor to SAP		28/2-20
Julie Rehné Lundgren[1,2]	MD, PhD-student	Contributor to SAP		4/3-20
Sten Madsbad[4]	Professor, DMSci	Principal investigator		29/2-20
Christian Rimer Juhl[1,2]	MD, PhD-student	Co-investigator		28.2-20
Bente Merete Stallknecht[1]	Professor, DMSci	Co-investigator		28/2-20
Jens Juul Holst[1,2]	Professor, DMSci	Co-investigator		3/3-20

<u>Affiliations</u>
[1]Department of Biomedical Sciences, University of Copenhagen, Copenhagen, Denmark
[2]Novo Nordisk Foundation Center for Basic Metabolic Research, University of Copenhagen, Copenhagen, Denmark
[3]Steno Diabetes Center Copenhagen, Gentofte, Denmark
[4]Department of Endocrinology, Hvidovre Hospital, Hvidovre, Denmark

Contents

Section 1: Administrative information 1
Signature page 2
Section 2: Introduction 5
7 Background and Rationale (adapted from published protocol(1)) 5
8 Objectives 6
Section 3: Study Methods 7
9 Trial design (from published protocol(1)) 7
10 Randomization 8
11 Sample size 8
12 Framework 8
13 Statistical interim analyses and stopping guidance 9
14 Timing of final analyses 9
15 Timing of outcome assessments 9
Section 4: Statistical Principles 9
16-18 Confidence intervals and P values 9
19-20 Adherence and protocol deviations and Analysis populations 9
Section 5: Trial Population 10
21 Screening data 10
22 Eligibility 10
23 Recruitment 11
24 Withdrawal/follow-up 11
25 Baseline participant characteristics 11
Section 6: Analysis 12
26 Outcome definitions 12
27 Analysis methods 14
28 Missing data 15
29 Additional analyses 15
30 Harms 15
31 Statistical software 15
References 15

Section 2: Introduction

7 Background and Rationale (adapted from published protocol(1))

Obesity is associated with increased risk of developing cardiovascular disease and type 2 diabetes (T2D), along with increased risk of all-cause mortality (3,4). Obesity management guidelines recommends weight loss of more than 5 % of initial body weight to improve cardiometabolic risk factors, with greater weight loss producing greater benefits (5,6). However, weight regain reverse these health benefits (7,8). Furthermore, intentional weight loss is typically followed by a 30 to 50 % regain of lost weight within the first year (9–11). The main biological reasons for the rapid weight regain may be that weight loss causes a decrease in total energy expenditure to a degree that is greater than predicted from the decrease in fat and lean mass (12,13) in combination with increased appetite in the weight-reduced state (14,15).

Increasing energy expenditure by increasing physical activity is the first-line lifestyle modification in the treatment of obesity along with reducing food intake. For exercise interventions targeting general public health recommendations (at least 150 min/week of moderate intensity aerobic exercise), the associated weight loss is often modest (0-3 %) without concomitant calorie restriction (16–18). However, with exercise almost exclusively fat mass is lost whereas lean mass is close to unchanged (19–22) thereby improving body composition. Furthermore, independent of weight loss, exercise improves glycemic control, low grade inflammatory profile and cardiorespiratory fitness in individuals with overweight and obesity (21,23–25).

Glucagon-like peptide-1 (GLP-1) is an incretin hormone secreted from L-cells in the gut after food intake. GLP-1 stimulates glucose-dependent insulin secretion thereby lowering blood glucose and reduces appetite and thereby food intake (26,27). Treatment for 56 weeks with the GLP-1 receptor agonist (GLP-1 RA), liraglutide (3.0 mg), as an adjunct to regular diet and physical activity recommendations has been shown to improve glycemic control and induce moderate weight loss of 4.0 % in patients with T2D (28) and 5.4 % in non-diabetic individuals with overweight or obesity (29) compared to placebo. In addition, liraglutide has been shown to maintain a diet-induced weight loss over 56 weeks (30) and maintain very low-calorie diet-induced improvements of fasting plasma glucose and triglycerides over 52 weeks of weight loss maintenance superior to similar diet-induced weight loss maintenance in obese individuals (15). Weight loss with liraglutide is a result of both reduced fat and lean mass (31,32).

Obesity is associated with chronic low-grade inflammation (33,34) which is linked to the development of atherosclerosis and insulin resistance (35–37). Physically active individuals have lower inflammatory biomarker concentrations than their inactive counterparts (24), possibly explained by anti-inflammatory effects of an acute bout of exercise (38) and lower levels of visceral adipose tissue (39). GLP-1 has also emerged as an immunomodulatory agent, as illustrated by GLP-1 analogue administration exerting anti-inflammatory actions in various cells, including endothelial cells, adipocytes, peripheral blood mononuclear cells, and in plasma (40–44). Notably, in patients with T2D and high cardiovascular disease risk, GLP-1 RAs reduced the rate of occurrence of first major cardiovascular event (45,46). Thus, both physical activity and GLP-1 RA treatment seem to facilitate weight loss maintenance, improve metabolic health, and reduce systemic inflammation. However, diet-induced weight loss decreases energy expenditure and increases appetite.

We hypothesize that the combination of physical activity and liraglutide treatment improves weight loss maintenance, body composition and immunometabolic health since the decreased energy expenditure is targeted with exercise and the increased appetite with liraglutide.

8 Objectives

The overall objectives of this study are to investigate the maintenance of weight loss, body composition and immunometabolic health outcomes over 52 weeks with liraglutide treatment, physical exercise, and the combination in individuals with obesity after a very low-calorie diet.
The hypothesis hierarchy was not clearly specified in the protocol and we hereby define it as described below.

Hypothesis hierarchy for the primary outcome:

1. We hypothesize that the combined treatment (exercise+liraglutide) is superior to placebo for maintaining total body weight loss. Superiority is claimed if the estimated difference between the changes in total body weight for the two groups (delta for exercise+liraglutide from V1 to V3 – delta for placebo from V1 to V3) favorizes the exercise+liraglutide group (diffence in change ≤ -4kg) and the CI95% for the estimated difference does not include 0.

2. We hypothesize that liraglutide is superior to placebo for maintaining total body weight loss. Superiority is claimed if the estimated difference between the changes in total body weight for the two groups (delta for liraglutide from V1 to V3 – delta for placebo from V1 to V3) favorizes the liraglutide group (diffence in change ≤ -4kg) and the CI95% for the estimated difference does not include 0.

3. We hypothesize that the combined treatment (exercise+liraglutide) is non-inferior to liraglutide for maintaining total body weight loss. Non-inferiority is claimed if the upper limit of the CI95% for the estimated difference between the changes in total body weight for the two groups (delta for exercise+liraglutide from V1 to V3 – delta for liraglutide from V1 to V3) is < +4kg.

4. We hypothesize that exercise is non-inferior to placebo for maintaining total body weight loss.
   Non-inferiority is claimed if the upper limit of the CI95% for the estimated difference between the changes in total body weight for the two groups (delta for exercise from V1 to V3 – delta for placebo from V1 to V3) is < +4kg.

5. We hypothesize that the combined treatment (exercise+liraglutide) is superior to exercise for maintaining total body weight loss. Superiority is claimed if the estimated difference between the changes in total body weight for the two groups (delta for exercise+liraglutide from V1 to V3 – delta for exercise from V1 to V3) favorizes the exercise+liraglutide group (diffence in change ≤ -4kg) and the CI95% for the estimated difference does not include 0.

Secondary outcome:

6. We hypothesize that the combined treatment (liraglutide+exercise) is superior to liraglutide for body composition (total body fat percentage). Superiority is claimed if the estimated difference between the changes in total body fat percentage for the two groups (delta for liraglutide+exercise from V1 to V3 – delta for liraglutide from V1 to V3) favorizes the liraglutide+exercise group (diffence in change ≤ -1.5%) andthe CI95% for the estimated difference does not include 0.

7. We hypothesize that exercise is superior to placebo for body composition (total body fat percentage). Superiority is claimed if the estimated difference between the changes in total body fat percentage for the two groups (delta for exercise from V1 to V3 – delta for placebo from V1 to V3) favorizes the exercise group (diffence in change ≤ -1.5%) and the CI95% for the estimated difference does not include 0.

8. We hypothesize that the combined treatment (exercise+liraglutide) is non-inferior to exercise for body composition (total body fat percentage). Non-inferiority is claimed if the upper limit of the CI95% for the estimated difference between the changes in total body weight for the two groups (delta for exercise from V1 to V3 – delta for placebo from V1 to V3) is < +1.5%.

Section 3: Study Methods

9 Trial design (from published protocol(1))

The S-LiTE trial (acronym for 'Synergy effect of the appetite hormone GLP-1 (LiragluTide) and Exercise on maintenance of weight loss and health after a low calorie diet') is an investigator-initiated, randomized, placebo-controlled, parallel group trial. The trial is triple-blinded with regards to study medication but not exercise intervention. The trial is registered at the European Clinical Trials Database (EudraCT Nr.: 2015-005585-32).

Description of Interventions

Diet-induced weight loss: Initially, all participants will undergo eight weeks with a very low-calorie diet (VLCD) (Cambridge Weight Plan, 800 kcal/day) with the objective to lose at least 5 % of body weight. Although benefits are evident already at modest weight loss of 2-3 % (e.g. triglycerides and HbA1c) (6), a ≥ 5 % cut-off after 8 weeks is chosen because fast weight losers have obtained a greater weight reduction and long-term maintenance, and were not more susceptible to weight regain than gradual weight losers (47). Participants who have lost at least 5 % of body weight after the eight-week weight loss phase will be randomized to one of the four study groups: 52 weeks of treatment with 1) placebo, 2) exercise + placebo, 3) liraglutide, or 4) exercise + liraglutide.

Liraglutide or placebo

The GLP-1 RA, liraglutide (3.0 mg), or placebo will be administrated once daily as subcutaneous injections in the abdomen or thigh. The starting dose is 0.6 mg with weekly increments of 0.6 mg until 3.0 mg is achieved. Participants who do not tolerate the 3.0 mg dose may in special circumstances stay at a lower dose (2.4 mg). However, the aim is to reach 3.0 mg for all study participants.

Physical exercise

The exercise intervention follows WHO's global recommendations on physical activity for health of 150 minutes of moderate-intensity aerobic physical activity throughout the week or 75 minutes of vigorous-intensity aerobic physical activity throughout the week or an equivalent combination of moderate- and vigorous-intensity activity (48). The intervention aims for four sessions per week. Two sessions per week will be performed under supervision of the study staff and two sessions will be performed individually. Supervised sessions will consist of structured exercise for a duration of 45 min. Of this, 30 min will be interval-based spinning and 15 min will be circuit training focusing on large muscle groups. Individual exercise sessions will include aerobic exercise such as cycling, rowing, or elliptical training as well as brisk walking or cycling to work. Participants randomized to an exercise group will undergo a 6-week ramp-up phase with exercise

before exercising four times per week from week 7 to 52. Participants not randomized to exercise will be instructed to maintain habitual physical activity according to level before entering the trial.

Liraglutide and physical exercise

Combination of the two interventions described above.

Study visits

Identical test days will take place before the initial weight loss phase (V0, week -8), after initial weight loss (V1, week 0) and after 52 weeks of treatment (V3). For details on test days, see study protocol (1).

Weight consultations will be performed weekly during initial VLCD phase and at week 1, 2, 3, 4, 9, 13, 17, 22, 26, 32, 39 and 46. Fasting blood samples will be collected at week 4, 13, 26 and 39.

Finally, participants will be invited to complete a post-trial unsupervised follow-up visit (V4) 1 year after intervention completion.

10 Randomization

After the initial eight-week VLCD phase, participants will be randomized after the test day (V1) to one of the four study groups in a 1:1:1:1 ratio in accordance with a subject randomization list (SRL) provided by Novo Nordisk. An un-blinded study nurse (not otherwise associated with the trial) will allocate study participants according to the SRL. Randomization will be stratified by sex (male/female) and age (below/above 40 years).

11 Sample size

Sample size calculation for total body weight:

Sample size for primary endpoint was calculated in relation to total body weight. Based on previous weight loss studies with liraglutide (15,49), we estimate the response within each treatment group to be normally distributed with a standard deviation of 5.5 kg. Thus, with 30 participants completing each study arm we will be able to detect a difference in delta of 4 kg between groups with a power of 0.8, assuming a two-sided α-level of 0.05. We will need 34 completers in each arm in order to attain a statistical power of 0.85 and 40 completers in each arm to attain a statistical power of 0.90. In our previous study, 10 % of participants who entered the initial VLCD phase did not complete this phase (15). With 222 recruited study participants and an expected dropout rate of 25 % after randomization, we expect to have at least 30 participants from each study arm to complete the trial.

Sample size for total body fat percentage

Sample size for secondary endpoint body composition was calculated in relation to total body fat percentage. Based on previous exercise trials (20,50–52) the response within each treatment group was estimated to have a standard deviation of 2.1%. Thus, with 32 participants completing each study arm we will be able to detect a difference in delta body fat percentage of 1.5% between groups with a power of 0.8, assuming a two-sided α-level of 0.05. We will need 36 completers to attain a statistical power of 0.85. Sample size calculation for fat percentage was performed in relation to writing the SAP; after the intervention was started but prior to the termination of the intervention.

12 Framework

See point 8.

13 Statistical interim analyses and stopping guidance

No interim analyses were planned and no guidelines for terminating the trial early was made.

14 Timing of final analyses

Results from V1 to V3 will be analyzed when the final participant completes the trial. Analyses including V4 will be performed when the final participant has completed V4.

15 Timing of outcome assessments

Body weight is measured at week -8 (V0), -7, -6, -5, -4, -3, -2, -1, 0 (V1), 1, 2, 4, 9, 13, 17, 22, 26, 39, 46, 52 (V3) and 104 (V4).
Body composition is measured at V0, V1, V3 and V4.
See point 26 for timing of descriptive/explorative outcome assessments.

Section 4: Statistical Principles

16-18 Confidence intervals and P values

P-values and 95% confidence intervals will be presented for comparisons (between and within group) and will not be adjusted for multiplicity. 95% confidence intervals will be presented for estimated levels and will not be adjusted for multiplicity. A hierarchical testing procedure will be used to control the type 1 error for tests with predefined hypotheses (see point 8); all subsequent tests will be regarded as descriptive/explorative if a test fails to confirm a given hypothesis. All non-hypothesis-based tests are per definition of a descriptive/exploratory nature. Statistical significance will be claimed if the null hypothesis is rejected at the alpha level of 0.05 (two-sided), i.e. the P-value of the null hypothesis test is $\leq$ 0.05. 95% confidence intervals will be required to support the tested hypothesis for hypothesis-based tests (see 8), and for exploratory/descriptive test on an absolute or relative scale not include 0 or 1 respectively.

19-20 Adherence and protocol deviations and Analysis populations

Intention-to-treat (ITT) analysis set:
All participants analyzed as randomized.

Per Protocol (PP) analysis set:
All participants who complete the 52-week randomized treatment period with sufficient compliance to study medication and/or exercise protocol as defined by:
Study medication: Having administered 2.4 or 3.0 mg subcutaneous (sc) liraglutide/placebo for at least 75% of the intervention period (measured by self-reporting during the 12 visits from week 1 to 52 after up-titration). Compliance to study medication will be summarized as number and percentage of participants equal to or above 75 %.
Exercise: Sports watches with heart rate monitors will be worn during all exercise sessions. For all participants in an exercise group, we will report duration of exercise (minutes per week), mean intensity during exercise (percentage of maximum heart rate) and exercise frequency (times per week). Exercise duration, intensity and frequency will be summarized as median plus 25th and 75th percentiles. Heart rate will be measured with a frequency of 1 Hz during all exercise. Relative exercise intensity for each heart rate measurement will be

classified based on percentage of maximum heart rate (determined during an maximal incremental cycle ergometer test) in accordance with ACSM's position stand on prescribing exercise(53), i.e. very light intensity (<57% of HR_{max}), light intensity (57-63% of HR_{max}), moderate intensity (64-76% HR_{max}), vigorous intensity (77-95% HR_{max}) and near-maximal to maximal intensity ($\geq$ 96% HR_{max}).
For exercise sessions where heart rate data is missing (e.g. due to forgotten watch for supervised exercise), the exercise duration will be noted and the participants average intensity and time at different intensity zones will be imputed. Exercise will be averaged for all weeks after the ramp-up phase and until end-of-trial test day and exercise compliance will be calculated as percentage of WHO's global recommendations on physical activity for health (48): *Adults aged 18–64 should do at least 150 minutes of moderate-intensity aerobic physical activity throughout the week or do at least 75 minutes of vigorous-intensity aerobic physical activity throughout the week or an equivalent combination of moderate- and vigorous-intensity activity.* Thus, for example, one minute of moderate-intensity exercise will account for 1/150 and one minute of vigorous- or near-maximal to maximal intensity exercise will account for 1/75 of prescribed weekly exercise. Per protocol analysis set will be defined as meeting at least 75% of global recommendations.

For participants to be included in the per protocol analysis:
a) Liraglutide group: Having administered 2.4 or 3.0 mg sc liraglutide for at least 75% of the intervention period.
b) Placebo group: Having administered 2.4 or 3.0 mg sc placebo for at least 75% of the intervention period.
c) Exercise + placebo group: Having administered 2.4 or 3.0 mg sc placebo for at least 75% of the intervention period and having met at least 75% of global recommendations on physical activity.
d) Exercise + liraglutide group: Having administered 2.4 or 3.0 mg sc liraglutide for at least 75% of the intervention period and having met at least 75% of global recommendations on physical activity.

Section 5: Trial Population

21 Screening data

Screening data will not be reported.

22 Eligibility

Inclusion criteria:

- BMI > 32 and < 43 (kg/m^2)
- Age >18 and <65 years
- Safe contraceptive method

Exclusion criteria:

- Patients diagnosed with *known* serious chronic illness including type 1 or 2 diabetes (or a randomly measured fasting plasma glucose > 7 mmol/l)
- Angina pectoris, coronary heart disease, congestive heart failure (NYHA III-IV)
- Severe renal impairment (creatinine clearance (GFR) <30 mL/min)
- Severe hepatic impairment
- Inflammatory bowel disease
- Diabetic gastroparesis

- Cancer
- Chronic obstructive lung disease
- Psychiatric disease, a history of major depressive or other severe psychiatric disorders
- The use of medications that cause clinically significant weight gain or loss
- Previous bariatric surgery
- A history of idiopathic acute pancreatitis
- A family or personal history of multiple endocrine neoplasia type 2 or familial medullary thyroid carcinoma
- Osteoarthritis which is judged to be too severe to manage the exercise programme. As intended per study design the intervention will include a 5% weight loss prior to randomization, thus it is expected that possible participants with mild form of osteoarthritis will be able to manage exercise prescriptions.
- Pregnancy, expecting pregnancy or breast feeding. If a study participant is in doubt whether she could be pregnant, a urine pregnancy test is performed. Females of childbearing potential who are not using adequate contraceptive methods (as required by local law or practice). Adequate contraception must be used throughout the study period and at least 65 hours after discontinuation of trial medication (65 hours corresponds to 5 times the half-life of liraglutide).
- Allergy to any of the ingredients/excipients of the study medication: liraglutide, disodium phosphate dihydrate, propylene glycol, phenol, hydrochloric acid, sodium hydroxide.
- Regular exercise training at high intensity (e.g. spinning) >2 hours per week.

23 Recruitment

The flow chart of the trial will follow the CONSORT guidelines and will include the number of participants who a) received oral information, b) were assessed for eligibility at prescreening, c) were included in the trial, d) attended first test day and initiated VLCD, e) were withdrawn or excluded from the VLCD phase, f) were randomized, g) were allocated to the four intervention groups, h) lost to follow-up, i) discontinued the intervention, and j) were analyzed in the end.

24 Withdrawal/follow-up

If a randomized participant withdraws, we will offer to perform all or some of the investigations planned at V3. The number/frequency of participants lost to follow-up (those not attending a weight measurement at V3) will be provided for each group and for each time point. If possible, the reasons for participants not completing the trial will be given. Summary of baseline levels for variables reported in the baseline table will be provided for completers and non-completers. Spaghetti plots will be used to visualize levels of the main outcome for completers and non-completers.

25 Baseline participant characteristics

The distribution of all outcomes included in baseline characteristics will be visually inspected using QQ-plots and histograms; those with a Gaussian distribution will be presented as means and standard deviations and those with a non-Gaussian distribution will be presented as medians plus 25th and 75th percentiles.

The following outcomes will be included in the baseline participant characteristics table for all participants combined at V0, all participants combined at V1 and participants divided by randomization group at V1:

- Number of participants (men/women)
- Age (years)
- Weight (kg)
- BMI (kg/m^2)
- Waist circumference (cm)
- Total fat mass (kg)
- Total fat free mass (kg)
- Body fat percentage (%)
- Systolic blood pressure (mmHg)
- Diastolic blood pressure (mmHg)
- HbA1c (mmol/mol)
- Fasting glucose (mmol/L)
- Fasting insulin (pmol/L)
- Fasting C-peptide (pmol/L)
- Glucose AUC (mmol/L*min)
- Insulin AUC (pmol/L*min)
- Cholesterol: total, LDL and HDL (mmol/L)
- Triglycerides (mmol/L)
- Peak oxygen consumption (ml/min)
- Peak oxygen consumption (ml/min/kg)
- HOMA-IR (fasting insulin (pmol/L) * fasting glucose (mmol/L) / 22.5)
- Matsuda index (10000/sqrt(fasting glucose * fasting insulin * mean glucose * mean insulin)
- SF-36 (scoring of eight health concepts ranging from 0-100: physical functioning, role limitations due to physical health problems, role limitations due to personal or emotional problems, energy/fatigue, emotional well-being, social functioning, pain, and general health perception)
- ALAT (U/L)
- Amylase (U/L)
- CRP (mg/L)

Section 6: Analysis

26 Outcome definitions

Primary outcome: Primary outcome is the change in total body weight (measured to the nearest 0.1 kg) from baseline (V1) to end-of-treatment (V3). A weight difference of 2.5-4.5 % body weight, obtained with liraglutide or semaglutide compared to placebo, have been associated with beneficial cardiovascular outcomes (45,46). A weight difference of 1-5% of body weight obtained with exercise has been associated with beneficial cardiovascular disease risk factors (17,18) and prevention of diabetes (54).
Thus, a difference in delta of 3-5% of total body weight has been defined as the minimal important difference. With a study population consisting of men and women (expected mean height of 170-174 cm based on previous lifestyle trials performed in Denmark (15,55,56) and a BMI range of 32-43 kg /m^2, a difference of 3-5% of total body weight will correspond to 4 kg.

Secondary outcome: Secondary outcome body composition is defined as change in total body fat percentage (measured to the nearest 0.01 % in fasted state with dual-energy X-ray absorptiometry (DXA) scans) from V1 to V3. In most exercise interventions with aerobic exercise, almost exclusively fat mass is lost whereas lean mass is close to unchanged (19–22). In weight loss studies with liraglutide, a combination of fat and lean mass is lost (31,32). The clinically relevant effect size for changes in fat percentage is not well described or investigated. However, based on the results of previous exercise interventions (20,57), a decrease in fat percentage equivalent of approximately 1.5 % seems to be physiologically relevant.

Descriptive/explorative outcomes:
Change from V1 to V3:

- Body composition (fat free mass (kg) and fat mass (kg))
- Circulating biomarkers of metabolic regulation to evaluate metabolic health (fasting and 3h AUC glucose (mmol/L and mmol/L*min) and fasting and 3h AUC insulin (pmol/L and pmol/L*min) for HOMA-IR and Matsuda index, HbA1c (mmol/mol), waist circumference (cm), systolic and diastolic blood pressure (mmHg), lipids (total cholesterol, HDL, LDL, and triglycerides (mmol/L))
- Safety (adverse events). Adverse events with an incident of ≥ 5 % in any treatment group and all serious adverse events will be reported by system organ class and preferred term for participants in all four treatment groups separately and for liraglutide pooled (exercise and non-exercise) and placebo pooled (exercise and non-exercise). Events will be reported as percentage of individuals experiencing an adverse event.
- Self-rated quality of life will be measured with the SF-36 questionnaire. Eight health concepts ranging from 0-100 will be scored: physical functioning, role limitations due to physical health problems, role limitations due to personal or emotional problems, energy/fatigue, emotional well-being, social functioning, pain, and general health perception.
- Peak oxygen uptake (absolute (ml/min) and relative (ml/min/kg))
- Stair test (S) and maximal isometric strenght test (N)
- Use of medication (n, frequency)
- Meal related appetite response
- Determination of daily physical activity and sleep
- Systemic markers of immune-metabolism and oxidation
- Endothelial function
- Immuno-metabolic changes in the subcutaneous adipose tissue
- Gene expression profile of circulating inflammatory cells
- Bone health
- Food preference questionnaire (LFPQ) and subjective appetite sensation
- Faecal bacterial composition
- Plasma metabolomics and proteomics
- Epigenetics of spermatozoa
- Questionnaires (TFEQ, IPAQ, G-SES, PSQI)

Outcomes measured at other time points:

- Body weight (week -7, -6, -5, -4, -3, -2, -1, 1, 2, 4, 9, 13, 17, 22, 26, 32, 39, 46 and V4)
- Number/proportion of participants that have reduced total body weight by ≥5%, ≥10% and ≥15% at V3 compared with V0
- Body composition (V0 and V4)
- Metabolic health (week 4, 13, 26, 39 and V4)
- Safety (adverse events) (week 1, 2, 3, 4, 9, 13, 17, 22, 26, 32, 39 and 46)
- Meal related appetite response (V0)
- Peak oxygen uptake, stair test and maximal isometric strenght test (V0)
- Systemic markers of immune-metabolism and oxidation (V0)
- Endothelial function (V0)
- Immuno-metabolic changes in the subcutaneous adipose tissue (V0 and week 26)
- Gene expression profile of circulating inflammatory cells (V0)
- Bone health (V0 and V4)
- Food preference questionnaire (LFPQ) (V0 and V4) and subjective appetite sensation (V0)
- Faecal bacterial composition (V0)
- Plasma metabolomics and proteomics (V0, week 4, 13, 26, 39 and V4)
- Epigenetics of spermatozoa (V0)
- Questionnaries (SF-36, TFEQ, IPAQ, G-SES, PSQI) (V0 and V4)
- Measurement of sleep and physical activity levels (V0, week 13, 26 and V4)

27 Analysis methods

Analyses of primary and secondary outcome (except for the pre-planned sensitivity analyses) will be performed based on the intention-to-treat (ITT) principle. All continuous endpoints/outcomes will be modelled using linear mixed effects models with the following fixed effects and interactions: Time (factorial), Treatment, Time (factorial)*Treatment, sex (female, male) and age group (<40 years, >40 years). The models will be specified with a restricted maximum likelihood estimation method, the Kenward-Roger degrees of freedom method, an unstructured covariance structure and a repeated effect for visit. Model fit will be evaluated using graphical methods and if necessary, outcomes will be log-transformed. Estimated mean differences (CI95%) for changes between groups (main study effects), conditional means (CI95%), and within group changes (CI95%) will be extracted from the model. For log-transformed outcomes the results will be back-transformed and be presented as the ratio between estimated relative changes (CI95%), estimated conditional geometric means (CI95%) and within group relative changes (CI95%), respectively. Between group differences in changes will be null-hypothesis tested and presented with P-values.
The following predefined sensitivity analyses will be performed for the primary and secondary outcome; an additional ITT analysis with adjustment for initial weight loss, an ITT analysis using multiple imputation to assess effects of missing data (see also 28), and finally an analysis of per protocol completers.

The analysis of the primary and secondary outcome will be performed blinded to group allocation by a researcher (Martin Bæk Blond) that have not been involved in the execution of the trial. The statistical and clinical/physiological implications of the results will be evaluated by the research team before un-blinding.

28 Missing data

The number/frequency of missing values for the primary and secondary outcome in each group at each time point will be provided. For the main analysis, the results will be based on likelihood inference and missing data will be assumed to be missing completely at random or missing at random. An additional analysis based on multiple imputation of missing values will be performed (see also 27). The dataset used for the imputations will include all observations for total body weight measured at or between V1-V3, and the auxiliary variables Sex (male or female) and Age (continuous, years). Under the assumption that weight changes in participants after loss to follow-up would resemble the development in the placebo groups rather than the development in the group to which they were originally randomized, the participants with missing values at V3 will be pooled with the placebo group and using a Markov chain Monte Carlo method all missing values will be imputed, assuming a multivariate normal distribution for the data, to create 1000 new datasets. Subsequently, the imputed datasets will be analyzed using the same mixed linear model used for the main analysis and averaged estimates calculated. If the main outcome has been transformed to fit the statistical model used for the main analysis this transformation will be applied in the imputation procedure.

29 Additional analyses

Not relevant

30 Harms

Adverse events (defined in the trial protocol) with an incident of ≥ 5 % in any treatment group and all serious adverse events will be reported by system organ class and preferred term for participants in all four treatment groups separately and for liraglutide pooled (exercise and non-exercise) and placebo pooled (exercise and non-exercise). Events will be reported as the number of and percentage of individuals experiencing an adverse event. Rates of adverse events will not be compared by null-hypothesis testing.

31 Statistical software

R version 3.6.0 or newer version (The R Foundation for Statistical Computing, www.R-project.org) and SAS version 9.4 or newer version (SAS Institute, Cary, NC, USA).

References

1 Jensen SBK, Lundgren JR, Janus C, Juhl CR, Olsen LM, Rosenkilde M *et al.* Protocol for a randomised controlled trial of the combined effects of the GLP-1 receptor agonist liraglutide and exercise on maintenance of weight loss and health after a very low-calorie diet. *BMJ Open* 2019; **9**: e031431.

2 Gamble C, Krishan A, Stocken D, Lewis S, Juszczak E, Doré C *et al.* Guidelines for the content of statistical analysis plans in clinical trials. *JAMA - J Am Med Assoc* 2017; **318**: 2337–2343.

3 Akram D, Astrup A, Atinmo T, Boissi J, Bray G, Carroll K *et al.* Obesity: Preventing and managing the global epidemic. World Health Organization. *Tech Rep Ser* 2000.

4 Abdelaal M, le Roux CW, Docherty NG. Morbidity and mortality associated with obesity. *Ann Transl Med* 2017; **5**: 161–161.

5 Garvey WT, Mechanick JI, Brett EM, Garber AJ, Hurley DL, Jastreboff AM *et al.* American association of clinical endocrinologists and American college of endocrinology comprehensive clinical practice guidelines for medical care of patients with obesity. *Endocr Pract* 2016; **22**: 1–203.

6 Jensen MD, Ryan DH, Apovian CM, Ard JD, Comuzzie AG, Donato KA *et al.* 2013 AHA/ACC/TOS Guideline for the Management of Overweight and Obesity in Adults. *Circulation* 2014; **129**: S102–S138.

7 Kroeger CM, Hoddy KK, Varady KA. Impact of weight regain on metabolic disease risk: A review of human trials. J Obes. 2014; **2014**: 614519.
8 Thomas TR, Warner SO, Dellsperger KC, Hinton PS, Whaley-Connell AT, Rector RS *et al.* Exercise and the metabolic syndrome with weight regain. *J Appl Physiol* 2010; **109**: 3–10.
9 Anderson JW, Konz EC, Frederich RC, Wood CL. Long-term weight-loss maintenance: a meta-analysis of US studies. *Am J Clin Nutr* 2001; **74**: 579–584.
10 Barte JCM, Ter Bogt NCW, Bogers RP, Teixeira PJ, Blissmer B, Mori TA *et al.* Maintenance of weight loss after lifestyle interventions for overweight and obesity, a systematic review. Obes Rev. 2010; **11**: 899–906.
11 Curioni CC, Lourenc -O PM. Long-term weight loss after diet and exercise: a systematic review. *Int J Obes* 2005; **29**: 1168–1174.
12 Rosenbaum M, Leibel RL. Adaptive thermogenesis in humans. *Int J Obes* 2010; **34**: S47–S55.
13 Leibel RL, Rosenbaum M, Hirsch J. Changes in Energy Expenditure Resulting from Altered Body Weight. *N Engl J Med* 1995; **332**: 621–628.
14 Iepsen EW, Lundgren J, Holst JJ, Madsbad S, Torekov SS. Successful weight loss maintenance includes long-term increased meal responses of GLP-1 and PYY3-36. *Eur J Endocrinol* 2016; **174**: 775–784.
15 Iepsen EW, Lundgren J, Dirksen C, Jensen JE, Pedersen O, Hansen T *et al.* Treatment with a GLP-1 receptor agonist diminishes the decrease in free plasma leptin during maintenance of weight loss. *Int J Obes* 2015; **39**: 834–841.
16 Swift DL, Johannsen NM, Lavie CJ, Earnest CP, Church TS. The Role of Exercise and Physical Activity in Weight Loss and Maintenance. *Prog Cardiovasc Dis* 2014; **56**: 441–447.
17 Donnelly JE, Blair SN, Jakicic JM, Manore MM, Rankin JW, Smith BK. Appropriate physical activity intervention strategies for weight loss and prevention of weight regain for adults. *Med Sci Sports Exerc* 2009; **41**: 459–471.
18 Shaw K, Gennat H, O'Rourke P, Del Mar C. Exercise for overweight or obesity. *Cochrane Database Syst Rev* 2006. doi:10.1002/14651858.CD003817.pub3.www.cochranelibrary.com.
19 Donnelly JE, Jacobsen DJ, Snyder Heelan K, Seip R, Smith S. The effects of 18 months of intermittent vs continuous exercise on aerobic capacity, body weight and composition, and metabolic ®tness in previously sedentary, moderately obese females. 2000www.nature.com/ijo (accessed 12 Nov2019).
20 Willis LH, Slentz CA, Bateman LA, Shields AT, Piner LW, Bales CW *et al.* Effects of aerobic and/or resistance training on body mass and fat mass in overweight or obese adults. *J Appl Physiol* 2012; **113**: 1831–1837.
21 Nordby P, Auerbach PL, Rosenkilde M, Kristiansen L, Thomasen JR, Rygaard L *et al.* Endurance training per se increases metabolic health in young, moderately overweight men. *Obesity* 2012; **20**: 2202–2212.
22 Foster-Schubert KE, Alfano CM, Duggan CR, Xiao L, Campbell KL, Kong A *et al.* Effect of diet and exercise, alone or combined, on weight and body composition in overweight-to-obese postmenopausal women. *Obesity* 2012; **20**: 1628–1638.
23 Bruce CR, Thrush AB, Mertz VA, Bezaire V, Chabowski A, Heigenhauser GJF *et al.* Endurance training in obese humans improves glucose tolerance and mitochondrial fatty acid oxidation and alters muscle lipid content. *Am J Physiol Endocrinol Metab* 2010; **291**: 99–107.
24 Beavers KM, Brinkley TE, Nicklas BJ. Effects of Exercise Training on Chronic Inflammation. *Clin Chim Acta* 2010; **411**: 785–793.
25 Lavie CJ, Arena R, Swift DL, Johannsen NM, Sui X, Lee DC *et al.* Exercise and the cardiovascular system: Clinical science and cardiovascular outcomes. *Circ Res* 2015; **117**: 207–219.
26 Flint A, Raben A, Astrup A, Holst JJ. Glucagon-like peptide 1 promotes satiety and suppresses energy intake in humans. *J Clin Invest* 1998; **101**: 515–520.
27 Holst JJ, Deacon CF, Vilsbøll T, Krarup T, Madsbad S. Glucagon-like peptide-1, glucose homeostasis and diabetes. *Trends Mol Med* 2008; **14**: 161–168.
28 Davies MJ, Bergenstal R, Bode B, Kushner RF, Lewin A, Skjøth TV *et al.* Efficacy of liraglutide for weight loss among patients with type 2 diabetes: The SCALE diabetes randomized clinical trial. *JAMA - J Am Med Assoc* 2015; **314**: 687–699.
29 Pi-Sunyer X, Astrup A, Fujioka K, Greenway F, Halpern A, Krempf M *et al.* A Randomized, Controlled Trial of 3.0 mg of Liraglutide in Weight Management. *N Engl J Med* 2015; **373**: 11–22.
30 Wadden TA, Hollander P, Klein S, Niswender K, Woo V, Hale PM *et al.* Weight maintenance and additional weight loss with liraglutide after low-calorie-diet-induced weight loss: The SCALE Maintenance randomized study. *Int J Obes* 2013; **37**: 1443–1451.
31 Jendle J, Nauck MA, Matthews DR, Frid A, Hermansen K, Düring M *et al.* Weight loss with liraglutide, a once-daily human glucagon-like peptide-1 analogue for type 2 diabetes treatment as monotherapy or added to

metformin, is primarily as a result of a reduction in fat tissue. *Diabetes, Obes Metab* 2009; **11**: 1163–1172.
32 Frøssing S, Nylander M, Chabanova E, Frystyk J, Holst JJ, Kistorp C *et al.* Effect of liraglutide on ectopic fat in polycystic ovary syndrome: A randomized clinical trial. *Diabetes, Obes Metab* 2018; **20**: 215–218.
33 Stępień M, Stępień A, Wlazeł RN, Paradowski M, Banach M, Rysz J. Obesity indices and inflammatory markers in obese non-diabetic normo- and hypertensive patients: A comparative pilot study. *Lipids Health Dis* 2014; **13**: 10–13.
34 Panagiotakos DB, Pitsavos C, Yannakoulia M, Chrysohoou C, Stefanadis C. The implication of obesity and central fat on markers of chronic inflammation: The ATTICA study. *Atherosclerosis* 2005; **183**: 308–315.
35 Libby P, Ridker PM, Maseri A. Inflammation and Atherosclerosis. *Circulation* 2002; **105**.
36 Duncan BB, Schmidt MI, Pankow JS, Ballantyne CM, Couper D, Vigo A *et al.* Low-grade systemic inflammation and the development of type 2 diabetes: The atherosclerosis risk in communities study. *Diabetes* 2003; **52**: 1799–1805.
37 Vozarova B, Weyer C, Lindsay RS, Pratley RE, Bogardus C, Antonio Tataranni P. High white blood cell count is associated with a worsening of insulin sensitivity and predicts the development of type 2 diabetes. *Diabetes* 2002; **51**: 455–461.
38 Starkie R, Ostrowski SR, Jauffred S, Febbraio M, Pedersen BK. Exercise and IL-6 infusion inhibit endotoxin-induced TNF-α production in humans. *FASEB J* 2003; **17**: 884–886.
39 Gleeson M, Bishop NC, Stensel DJ, Lindley MR, Mastana SS, Nimmo MA. The anti-inflammatory effects of exercise: Mechanisms and implications for the prevention and treatment of disease. *Nat Rev Immunol* 2011; **11**: 607–610.
40 Chaudhuri A, Ghanim H, Vora M, Sia CL, Korzeniewski K, Dhindsa S *et al.* Exenatide exerts a potent antiinflammatory effect. *J Clin Endocrinol Metab* 2012; **97**: 198–207.
41 Hogan AE, Gaoatswe G, Lynch L, Corrigan MA, Woods C, O'Connell J *et al.* Glucagon-like peptide 1 analogue therapy directly modulates innate immune-mediated inflammation in individuals with type 2 diabetes mellitus. *Diabetologia* 2014; : 781–784.
42 Garczorz W, Gallego-Colon E, Kosowska A, Kłych-Ratuszny A, Woźniak M, Marcol W *et al.* Exenatide exhibits anti-inflammatory properties and modulates endothelial response to tumor necrosis factor α-mediated activation. *Cardiovasc Ther* 2018; **36**. doi:10.1111/1755-5922.12317.
43 von Scholten BJ, Persson F, Rosenlund S, Eugen-Olsen J, Pielak T, Faber J *et al.* Effects of liraglutide on cardiovascular risk biomarkers in patients with type 2 diabetes and albuminuria: A sub-analysis of a randomized, placebo-controlled, double-blind, crossover trial. *Diabetes, Obes Metab* 2017; **19**: 901–905.
44 Lee YS, Park MS, Choung JS, Kim SS, Oh HH, Choi CS *et al.* Glucagon-like peptide-1 inhibits adipose tissue macrophage infiltration and inflammation in an obese mouse model of diabetes. *Diabetologia* 2012; **55**: 2456–2468.
45 Marso SP, Daniels GH, Brown-Frandsen K, Kristensen P, Mann JFE, Nauck MA *et al.* Liraglutide and Cardiovascular Outcomes in Type 2 Diabetes. *N Engl J Med* 2016; **375**: 311–322.
46 Marso SP, Bain SC, Consoli A, Eliaschewitz FG, Jodar E, Leiter LA *et al.* Semaglutide and cardiovascular outcomes in patients with type 2 diabetes. *N Engl J Med* 2016; **375**: 1834–1844.
47 Nackers LM, Ross KM, Perri MG. The association between rate of initial weight loss and long-term success in obesity treatment: Does slow and steady win the race? *Int J Behav Med* 2010; **17**: 161–167.
48 World Health Organization. Physical Activity and Adults - Recommended levels of physical activity for adults aged 18 - 64 years. https://www.who.int/dietphysicalactivity/factsheet_adults/ (accessed 8 Apr2019).
49 Astrup A, Rössner S, Van Gaal L, Rissanen A, Niskanen L, Al Hakim M *et al.* Effects of liraglutide in the treatment of obesity: a randomised, double-blind, placebo-controlled study. *Lancet* 2009; **374**: 1606–1616.
50 Mensberg P, Nyby S, Jørgensen PG, Storgaard H, Jensen MT, Sivertsen J *et al.* Near-normalization of glycaemic control with glucagon-like peptide-1 receptor agonist treatment combined with exercise in patients with type 2 diabetes. *Diabetes, Obes Metab* 2017; **19**: 172–180.
51 Skrypnik D, Bogdański P, Mądry E, Karolkiewicz J, Ratajczak M, Kryściak J *et al.* Effects of Endurance and Endurance Strength Training on Body Composition and Physical Capacity in Women with Abdominal Obesity. *Obes Facts* 2015; **8**: 175–187.
52 Slentz CA, Duscha BD, Johnson JL, Ketchum K, Aiken LB, Samsa GP *et al.* Effects of the Amount of Exercise on Body Weight, Body Composition, and Measures of Central Obesity: STRRIDE - A Randomized Controlled Study. *Arch Intern Med* 2004; **164**: 31–39.
53 Garber CE, Blissmer B, Deschenes MR, Franklin BA, Lamonte MJ, Lee IM *et al.* Quantity and quality of exercise for developing and maintaining cardiorespiratory, musculoskeletal, and neuromotor fitness in apparently

healthy adults: Guidance for prescribing exercise. *Med Sci Sports Exerc* 2011; **43**: 1334–1359.
54 Knowler WC, Barrett-Connor E, Fowler SE, Hamman RF, Lachin JM, Walker EA *et al.* Reduction in the incidence of type 2 diabetes with lifestyle intervention or metformin. *N Engl J Med* 2002; **346**: 393–403.
55 Blond MB, Rosenkilde M, Gram AS, Tindborg M, Christensen AN, Quist JS *et al.* How does 6 months of active bike commuting or leisure-time exercise affect insulin sensitivity, cardiorespiratory fitness and intra-abdominal fat? A randomised controlled trial in individuals with overweight and obesity. *Br J Sports Med* 2019; **53**: 1183–1192.
56 Iepsen EW, Zhang J, Thomsen HS, Hansen EL, Hollensted M, Madsbad S *et al.* Patients with Obesity Caused by Melanocortin-4 Receptor Mutations Can Be Treated with a Glucagon-like Peptide-1 Receptor Agonist. *Cell Metab* 2018; **28**: 23-32.e3.
57 Sigal RJ, Kenny GP, Boulé NG, Wells GA, Prud'homme D, Fortier M *et al.* Effects of aerobic training, resistance training, or both on glycemic control in type 2 diabetes: A randomized trial. *Ann Intern Med* 2007; **147**: 357–369.

Summary of changes from original to final statistical analysis plan:

**Amendment number (issue date)**	1 (04-Mar-2020)
**Timing of change (before/after FPFV 12-Sep-2016)**	After
**Rationale and key changes**	• Item 8 (Page 6-7): The minimally important differences (physiologically relevant changes) for the primary and secondary outcomes (which was defined in item 26) have now also been added to the hypotheses for clarity. In the hypotheses section of SAP version 1 it was stated that superiority would be claimed if the changes to an outcome favorised the given group (see individual hypotheses on page 6-7) and the lower limit of the CI95% for the estimated difference did not exceed 0. The latter part has been corrected to “does not include 0” to reflect that a reduction is favorable as stated in the hypothesis.<br>• Item 27 (Page 14): The model specification for the model used to assess changes to the primary and secondary outcomes was changed from a random intercept model to a repeated measures model in order to avoid the risk of falsely narrow confidence intervals and low P-values produced by the assumption of constant variance and covariance across repeated measurements.

FPFV, first patient first visit.
