# Supplementary material for: Effects of exercise and liraglutide on vascular health and inflammation during weight loss maintenance: a prespecified secondary analysis of the S-LiTE trial
Source: Nat Metab. 2026 Jun 24;8(7):1483–8. doi: 10.1038/s42255-026-01554-4 (PMC13400309; doi:10.1038/s42255-026-01554-4)
Supplement: Supplementary file 2 — Reporting Summary [file 42255_2026_1554_MOESM2_ESM.pdf]

nature portfolio

Corresponding author(s): Signe Sørensen Torekov
Rasmus Michael Sandsdal

Last updated by author(s): 05/26/2026

Reporting Summary

Nature Portfolio wishes to improve the reproducibility of the work that we publish. This form provides structure for consistency and transparency in reporting. For further information on Nature Portfolio policies, see our Editorial Policies and the Editorial Policy Checklist.

Statistics

For all statistical analyses, confirm that the following items are present in the figure legend, table legend, main text, or Methods section.

n/a	Confirmed	
☐	☒	The exact sample size (n) for each experimental group/condition, given as a discrete number and unit of measurement
☐	☒	A statement on whether measurements were taken from distinct samples or whether the same sample was measured repeatedly
☐	☒	The statistical test(s) used AND whether they are one- or two-sided<br>*Only common tests should be described solely by name; describe more complex techniques in the Methods section.*
☐	☒	A description of all covariates tested
☐	☒	A description of any assumptions or corrections, such as tests of normality and adjustment for multiple comparisons
☐	☒	A full description of the statistical parameters including central tendency (e.g. means) or other basic estimates (e.g. regression coefficient) AND variation (e.g. standard deviation) or associated estimates of uncertainty (e.g. confidence intervals)
☐	☒	For null hypothesis testing, the test statistic (e.g. F, t, r) with confidence intervals, effect sizes, degrees of freedom and P value noted<br>*Give P values as exact values whenever suitable.*
☒	☐	For Bayesian analysis, information on the choice of priors and Markov chain Monte Carlo settings
☒	☐	For hierarchical and complex designs, identification of the appropriate level for tests and full reporting of outcomes
☒	☐	Estimates of effect sizes (e.g. Cohen's d, Pearson's r), indicating how they were calculated

Our web collection on statistics for biologists contains articles on many of the points above.

Software and code

Policy information about availability of computer code

Data collection	QLAB v15.0, Philips; Research electronic data capture (REDCap)
Data analysis	Statistical analysis in SAS Enterprise Guide 8.1; Figures created in R version 4.5.1 using ggplot2 package version 4.0.3.

For manuscripts utilizing custom algorithms or software that are central to the research but not yet described in published literature, software must be made available to editors and reviewers. We strongly encourage code deposition in a community repository (e.g. GitHub). See the Nature Portfolio guidelines for submitting code & software for further information.

Data

Policy information about availability of data

All manuscripts must include a data availability statement. This statement should provide the following information, where applicable:

- Accession codes, unique identifiers, or web links for publicly available datasets
- A description of any restrictions on data availability
- For clinical datasets or third party data, please ensure that the statement adheres to our policy

Data from the study is not available for download due to restrictions under the General Data Protection Regulation (GDPR). De-identified data under the GDPR and local regulations of Denmark and the University of Copenhagen may be available for research collaboration purposes upon reasonable request to the corresponding author and will require the approval of the corresponding author and the completion of a data processing agreement.

Research involving human participants, their data, or biological material

Policy information about studies with human participants or human data. See also policy information about sex, gender (identity/presentation), and sexual orientation and race, ethnicity and racism.

Reporting on sex and gender	Participants were included in the study if they fulfilled all eligibility criteria, irrespective of sex (assigned at birth) and gender. The proportion of female or male is provided in Table 1. Sex was considered in the study design, as weight loss trials often comprise a vast majority of females, e.g., 80% in SCALE Obesity and Prediabetes. In our trial, we succeeded in having 38% male and 62% female participants. Sex was defined as assigned at birth and was registered by a physician in an electronic case report form.<br><br>Given the small sample sizes when disaggregating sex within the four-arm groups, we did not conduct post hoc sex-specific analyses, as the study was not adequately powered to draw meaningful conclusions from such comparisons. However, sex (assigned at birth) was an inclusion stratum in our trial, and we have accordingly included sex as a covariate in our mixed-effects model to adjust for potential differences between male and female participants in the analysis.
Reporting on race, ethnicity, or other socially relevant groupings	Participants were included in the study if they fulfilled all eligibility criteria, irrespective of race and ethnicity. The included participants reflected the local area of inclusion at Copenhagen University Hospital, Hvidovre.
Population characteristics	Participant characteristics are provided in Table 1. Body weight at inclusion was 36.9±2.9 kg and age 45±12 years. 38% were male and 62% female.
Recruitment	Recruitment was done via newspapers, online media, and flyers from the Department of Endocrinology, Copenhagen University Hospital – Amager and Hvidovre, Denmark, and Department of Biomedical Sciences, University of Copenhagen, Denmark. The trial population reflected the local area of Hvidovre, Denmark. All participants provided written informed consent before enrollment. All participants provided written informed consent before enrollment. Participants who completed the trial received compensation of 3,000 Danish kroner.
Ethics oversight	Protocol approval: H-16027082: The Ethical Committee of the Capital Region of Denmark and the Danish Medicines Agency. Oversight: Good Clinical Practice (GCP).

Note that full information on the approval of the study protocol must also be provided in the manuscript.

Field-specific reporting

Please select the one below that is the best fit for your research. If you are not sure, read the appropriate sections before making your selection.

☒ Life sciences ☐ Behavioural & social sciences ☐ Ecological, evolutionary & environmental sciences

For a reference copy of the document with all sections, see nature.com/documents/nr-reporting-summary-flat.pdf

Life sciences study design

All studies must disclose on these points even when the disclosure is negative.

Sample size	195 participants completed the low-calorie diet and were randomized to exercise (49), exercise+placebo (48), liraglutide (49), exercise +liraglutide (49), of which 130 participants completed the trial per-protocol. See CONSORT flow diagram, Extended Data Figure 1.
Data exclusions	The Statistical Analysis Plan included all participants who received at least 1 dose of randomized treatment. Two participants were randomized but did not receive a single dose of their randomized treatment and are not included in the analysis set.
Replication	N/A
Randomization	Randomization was done in a 1:1:1:1 ratio stratified by sex assigned at birth and age group (<40 years and ≥40 years). A non-blinded study nurse, not otherwise involved in any study-related procedures, performed randomization using an allocation list provided by Novo Nordisk.
Blinding	Allocation of participants was implemented by a qualified, unblinded study nurse who was not otherwise involved in trial conduct. The study participants, personnel, and investigators were blinded regarding study medication but not exercise during data collection. Investigators were blinded to group allocation in the assessment of the prespecified secondary outcome of this study.

Reporting for specific materials, systems and methods

We require information from authors about some types of materials, experimental systems and methods used in many studies. Here, indicate whether each material, system or method listed is relevant to your study. If you are not sure if a list item applies to your research, read the appropriate section before selecting a response.

Materials & experimental systems

n/a	Involved in the study
☐	☒ Antibodies
☒	☐ Eukaryotic cell lines
☒	☐ Palaeontology and archaeology
☒	☐ Animals and other organisms
☐	☒ Clinical data
☒	☐ Dual use research of concern
☒	☐ Plants

Methods

n/a	Involved in the study
☒	☐ ChIP-seq
☒	☐ Flow cytometry
☒	☐ MRI-based neuroimaging

Antibodies

Antibodies used

Rabbit anti-human vWF polyclonal IgG (DAKO, Glostrup, Denmark, Ref. Nr. A0082); mouse anti-human tPA monoclonal IgG as capture (clone 15-4-21) and detection (clone 15-4-6) antibodies, V-PLEX MSD MULTI-SPOT Assay System (Vascular Injury Panel 2 (human) Kits, K15198D; V-PLEX MSD Proinflammatory Panel 1 (human), K15049D.

Validation

vWF antigen (vWF, %) was determined by ELISA using rabbit anti-human vWF polyclonal IgG as capture and detection antibodies (DAKO, Glostrup, Denmark, Ref. Nr. A0082). tPA antigen (ng/mL) was determined by an in-house ELISA using mouse anti-human tPA monoclonal IgG as capture (clone 15-4-21) and detection (clone 15-4-6) antibodies. sICAM-1, sVCAM-1, and high-sensitivity CRP (hsCRP) were analyzed with a human V-PLEX MSD Multi-Spot Assay System. Pro-inflammatory cytokines were measured in duplicates using a V-PLEX MSD Proinflammatory Panel. See Methods for references. See Methods for references.

Clinical data

Policy information about clinical studies

All manuscripts should comply with the ICMJE guidelines for publication of clinical research and a completed CONSORT checklist must be included with all submissions.

Clinical trial registration

EudraCT number: 2015-005585-32; ClinicaTrials.gov number: NCT04122716.

Study protocol

The study protocol is provided with this paper.

Data collection

Participants were recruited between August 2016 and September 2018. Lats participant's last visit was November 2019. Data collection was done at the Department of Endocrinology, Copenhagen University Hospital – Amager and Hvidovre, Denmark, and Department of Biomedical Sciences, University of Copenhagen, Denmark.

Outcomes

Outcomes related to cardiovascular health were prespecified in the Statistical Analysis Plan. Main outcomes were carotid intima-media thickness (via ultrasound) and inflammatory and endothelial markers (via blood samples and assays). Please see details under Methods, Outcomes.

Plants

Seed stocks

NA

Novel plant genotypes

NA

Authentication

NA
