# Supplementary material for: Effects of exercise and liraglutide on vascular health and inflammation during weight loss maintenance: a prespecified secondary analysis of the S-LiTE trial
Source: Nat Metab. 2026 Jun 24;8(7):1483–8. doi: 10.1038/s42255-026-01554-4 (PMC13400309; doi:10.1038/s42255-026-01554-4)
Supplement: Supplementary file 3 — Peer Review File [file 42255_2026_1554_MOESM3_ESM.pdf]

nature portfolio

Peer Review File

Effects of exercise and liraglutide on vascular health and inflammation during weight loss maintenance: a prespecified secondary analysis of the S-LiTE trial

Corresponding Author: Professor Signe Torekov

Version 0:

Reviewer comments:

Reviewer #1

(Remarks to the Author)
In this manuscript, Sandsdal and colleagues present the findings of a study of individuals with obesity and without diabetes, who underwent a low calorie diet for 8 weeks with weight loss and then were randomized in a two-by-two factorial design to the presence or absence of exercise and the GLP-1 receptor agonist liraglutide. They report changes in carotid IMT, as a surrogate measure of atherosclerosis, and report differences – in particular a reduction in cIMT at 52 weeks in the exercise group, but not those treated with liraglutide. This is complemented by favorable effects on systemic biomarkers associated with atherosclerosis. The findings are interesting, although in its current form there is considerable ambiguity of the representation of the study and results. The authors should consider a number of points.
1. The data is presented in a form that is not consistent with the reported methodology. If I've read this correctly, there was a 2x2 design – in other words, half the patients received the exercise intervention and half did not, similarly half received the GLP-1 receptor agonist and the other half did not. The results present exercise and liraglutide as completely separate groups, which is not correct as one quarter of participants must have received both. The findings should be presented more accurately.
2. On the basis of this, I can't make any further assessment of the findings of this study.
3. Beyond, this there appears to be no control for baseline measurements, which is critical in serial imaging studies. While the authors have applied a linear model, accounting for time, baseline cIMT does not appear to be a covariate that has been accounted for.
4. It is also intriguing that the only data presented is change, there is no baseline data. The reviewer cannot be convinced that there were not differences between the groups that may have accounted for differences in changes in cIMT.
5. The authors should remove the references in the manuscript and figures suggesting potential reductions in clinical risk with the cIMT changes.
6. There is no other data given on medications.
7. The authors should acknowledge that cIMT is not the earliest feature of atherosclerosis. There is no literature, whatsoever, that demonstrates that cIMT appears first and then develops into plaque. It is simply a barometer of vascular health.
8. The lack of correlation between blood pressure and cIMT is problematic, it is particularly sensitive to blood pressure.
9. There is no data provided on variability between reading assessments.
10. It should be acknowledged that liraglutide is a relatively modest GLP-1 receptor agonist and the findings do not necessarily imply what may be encountered with more commonly used GLP-1 receptor agonists.
11. The study design is odd. Everyone underwent weight loss and then the interventions were applied, yet that is not how this would play out in clinical practice – the interventions are used to promote (and then hopefully maintain) the weight loss.

Reviewer #2

(Remarks to the Author)
I thank the authors for their manuscript. I offer the following methodological comments

1) Secondary analysis
i) Ensure that this is clear in the title and abstract that readers are aware this is a secondary analysis of an RCT - at present this is not clear
ii) Include the CONSORT checklist and Flowchart that is relevant for this study
iii) Include the primary study sample size
iv) Include the method of sequence generation

v) Introduction - state why this is an important question and how this was different to the primary publication

2) Methods
i) State the method for handling the missing data
ii) State the analysis model in full and justify the included covariates and compare this with the primary publication.
iii) Outcomes : list these clearly
iv) Blinding status of personnel - state clearly

3) Discussion
i) State how these findings fit with the primary publication
ii) Weaknesses - type-1 error due to a secondary publication

4) Protocol and SAP
i) Include both as supplementary material with version number and dates

5) Output
i) Table 1 - remove the between group comparison and include all 4 randomised groups with subtotals for the key comparison here
ii) Figure 1 - show both factors on each bar - improve the quality of the figure.

Reviewer #3

(Remarks to the Author)
Many thanks for asking me to review this article. The authors should be congratulated in conducting this exploratory study with such attention to detail and achieving high levels of follow up. There are a number of things that should be altered or included to improve the scientific validity of this study.

The purpose of this study is to determine the effect of liraglutide and exercise on imaging and circulating biomarkers of cardiovascular disease. The primary outcome of this study was change in the intima-media thickness of the carotid artery.

Firstly, the title “Exercise but not GLP-1-based therapy reduces early development of atherosclerosis during weight loss maintenance: a randomized controlled trial” is misleading. The study demonstrated that exercise reduced carotid intimal media thickness which is associated with a reduction in cardiovascular events. The reviewer feels the title should be tempered. The title implied that this study is comparing exercise with GLP-1 in the development of atherosclerosis but actually this has not been evaluated. There is no mention of plaque volume in this evaluation.

Results

The results are impressive even before randomisation. The reviewer would suggest that in the results section the final weight loss could be stated in parenthesis next to the subsequent weight loss or gain in each arm.

Was there any correlation with reduction in cIMT and reduction in markers of inflammation? If so this could be a helpful visual in the supplement for the reader and give more credit to the discussion.

Discussion
“In this randomized, controlled trial, we evaluated the impact of exercise and GLP-1-based therapy on the early development of atherosclerosis and vascular inflammation during weight loss maintenance in adults with obesity without diabetes”. The reviewer would suggest changing the early development of atherosclerosis to carotid intimal-media thickness.

The study asked all participants to eat a calorie deficient diet prior to being randomised. The reviewer feels that the rationale for this should be explained. It is clearly a strength of the study as it helps to standardise participants nutritional status at baseline and reduce any variation in circulating biomarkers that could be attributed to dietary factors alone.

The discussion section makes little in the way to explain whether this neutral effect of liraglutide on biomarkers and cIMT is drug specific or a class effect of GLP1. The reviewer feels this is an important aspect to address given the global use and demand for other GLP-1 agents.

Strengths and limitations

The reviewer feels that the section needs more thought.

To me the strengths are;
- Comprehensive follow up
- Enrollment of only those that manages to lose >5% of their body mass
- Standardisation of diet in the lead up to randomisation
- Inclusion of a number of emerging circulating biomarkers

Limitations

Relatively short follow up and whether these results are sustainable in the longer term is is uncertain

The impact of these results on clinical outcomes is unknown

There was intensive input to deliver the exercise intervention and this has important implications for the generalisability of the findings of this study into routine clinical practice.

Reproducibility of doppler USS

Methodology

Definition of obesity
The authors state a BMI 32-43kg/m2 as opposed to >30kg/m2. Why was this higher threshold selected and why was there an upper limit to this?

Timing of randomisation
The timing of randomisation should be more explicit. Suggest something like “On completion of an 8-week low calorie diet, only participants who lost at least 5% of their initial body weight were eligible for enrolment and subsequently randomised in this study”.

Reproducibility of doppler USS
There are no Bland-Altman plots to show reproducibility. Reproducibility may not have been done but then the authors should state this in the limitations.

The reviewer also feels that the use of carotid USS needs a little more explanation in the discussion. Why was this modality selected? The use of carotid USS has no RCT data to show its efficacy in the primary prevention of cardiovascular disease and this should be stated.

Exercise intervention
Participants randomised to exercise were subject to several support strategies that those randomised to nothing or liraglutide did not receive. These patients, by the nature of the study protocol, had more contact with researchers and this in as of itself may have led to a Hawthorne effect.

Circulating biomarkers

The measurement of several biomarkers is a strength of the study. The reviewer would suggest that the paper could be made stronger by including the coefficient of variation, limit of detection, and limit of quantification each biomarker in a supplementary table. The reviewer feels that a little more discussion on why the changes observed in the conduct of this study are beneficial to participants is warranted to strengthen the study findings and highlight potential relevance to clinical practice.

Statistical analysis
Power calculation appears to be missing here. What was the level of power? How was this calculated.

Table 1
Sex needs percent added to this
How was blood pressure recorded – in office or at home? Was this averaged over three readings?

Summary

Overall this is an interesting and well conducted piece of work. The comments and suggestions have been provided to improve the scientific rigor of the paper and to tease out interesting comments.

Version 1:

Reviewer comments:

Reviewer #1

(Remarks to the Author)
In this revised manuscript, now presented as a brief communication, Sandsdal and colleagues report the findings of a 2x2 factorial trial of exercise and liraglutide on changes in carotid IMT. The authors are thanked for the substantial changes that have been made and responses to the reviewer comments. It makes this much easier to understand and review. The main findings, that exercise interventions, but not liraglutide, has favorable effects on changes in cIMT are clear. The authors should consider a number of points.
1. My main concern remains the study design. The run of weight loss for all is problematic for interpretation of all subsequent findings and as pointed out in my previous commentary is artificial compared with the real world.

2. The interpretation that the findings suggest that GLP-1 receptor agonists effects on cardiovascular health must be mediated via weight loss on the basis of the lack of findings on cIMT should be toned down. I’m not convinced this study provides any mechanistic insights and given the pre-existing weight loss, it is impossible to interpret this. It also contrasts with findings of large cardiovascular outcomes trials in the GLP-1 space, in which reported benefits appear earlier than substantial weight loss, arguing the opposite point from the authors of this manuscript.
3. Triglycerides should be presented as median (interquartile range).
4. It is surprising that hsCRP was not measured, when a number of inflammatory markers were included.

Reviewer #2

(Remarks to the Author)
I thank the editors and authors for their responses. No further comments

Reviewer #3

(Remarks to the Author)

Open Access This Peer Review File is licensed under a Creative Commons Attribution 4.0 International License, which permits use, sharing, adaptation, distribution and reproduction in any medium or format, as long as you give appropriate credit to the original author(s) and the source, provide a link to the Creative Commons license, and indicate if changes were made.
In cases where reviewers are anonymous, credit should be given to 'Anonymous Referee' and the source.
The images or other third party material in this Peer Review File are included in the article’s Creative Commons license, unless indicated otherwise in a credit line to the material. If material is not included in the article’s Creative Commons license and your intended use is not permitted by statutory regulation or exceeds the permitted use, you will need to obtain permission directly from the copyright holder.
To view a copy of this license, visit https://creativecommons.org/licenses/by/4.0/

Point-by-point

Your manuscript "Exercise but not GLP-1-based therapy reduces early development of atherosclerosis during weight loss maintenance: a randomized controlled trial" has now been seen by 3 referees, whose comments are appended below. Although they find your work of potential interest, they have raised substantive concerns which in our view are sufficiently important to preclude publication of the work in Nature Metabolism, at least in its present form.

In general, we agree with the reviewers that the work would be of interest to the broad audience of Nature Metabolism. At the same time, the review process identified several opportunities to strengthen the work. We would be happy to look at a revised manuscript that has been revised accordingly (unless something similar has been accepted at Nature Metabolism or appeared elsewhere in the meantime). Please also note that any revised manuscript, submitted later than one year after this decision, will be re-evaluated for conceptual advance and overall interest to the wider metabolism research community, to determine its suitability for publication in Nature Metabolism at that time.

Response to editors' comments:
Dear Jean,

Thank you for considering our manuscript for publication in *Nature Metabolism* and for the opportunity to improve our work.

We are grateful to the three reviewers for their constructive feedback, which has helped us strengthen the manuscript. We have carefully considered each comment and have revised the manuscript accordingly. Below, we provide a detailed point-by-point response to all concerns raised.

Comments by you and the Reviewers are shown in **bold**, and our responses follow in plain text with blue font.

More specifically, it will be particularly important to the editors that you address the following concerns:

1) As requested by Reviewers #1 and #2, we ask you to revise the results section to reflect the four randomized groups (placebo, exercise, liraglutide, exercise + liraglutide), rather than collapsing them into a 2x2 factorial design. This applies to the text, statistical analyses, as well as figures and tables presented as Main Display Items and Extended Data Items. The 2x2 factorial design analysis can be moved to Supplementary Data.

Authors' response:

We agree and have revised the manuscript accordingly:

- The manuscript now presents outcomes for all four randomized intervention groups separately (i.e., placebo, exercise, liraglutide, and exercise + liraglutide).
- All primary inferences in this revised manuscript are based on the four-group parallel randomized comparisons. The 2x2 factorial analyses have been moved to Supplementary Data.
- Data visualizations have been remade into a Main Display Item, and tables have been updated to show the four intervention groups individually.

Importantly, the analysis of the individual intervention groups shows that, after a diet-induced weight loss and standardization of nutritional and metabolic status, exercising participants were able to improve their carotid intima-media thickness and inflammation levels in a weight-loss-independent manner, whereas GLP-1 RA treatment alone showed no such improvements despite a similarly maintained weight loss. These findings show that the cardiovascular benefits of exercise extend beyond weight reduction, while the effects of GLP-1-based therapy on vascular health may be predominantly mediated by weight loss.

2) Please test and report the association between changes in cIMT and inflammatory/endothelial markers measured in the study.

Author's response:

We have tested the association between changes in cIMT and biomarkers and reported the results in Supplementary Table S4. No significant associations were observed between changes in circulating biomarkers and changes in cIMT. This may reflect the relatively small sample size and homogeneous study population, which limits statistical power to detect such associations.

As mentioned by a reviewer, we also tested the association between changes in cIMT and blood pressure. The results are now reported in the Supplementary Table S4. Regarding blood pressure, during the low-calorie diet, systolic blood pressure was reduced substantially by 10.3 mmHg to a mean of 122 mmHg. In the follow-up period, blood pressure did not differ significantly across the groups, which may explain the absence of a correlation, as blood pressure was normalized during the one-year intervention. We interpret the lack of association as supporting cIMT as an independent marker of vascular health in the context of our study, as has been previously suggested in the literature.[1]

Collectively, we now report in the Results paragraph, lines 90-92.

"At inclusion, cIMT was positively associated with blood pressure, but changes in cIMT during the one-year treatment were not associated with changes in blood pressure or biomarkers (Supplementary Table S4)."

3) As requested by Reviewers #1 and #3, we ask you to tone down overstated claims regarding clinical atherosclerotic risk, and to replace the term "atherosclerosis" throughout the manuscript with "cIMT" and emphasize its role as a surrogate endpoint.

Author's response:

We agree and have revised the manuscript in accordance with the reviewers' requests. We have replaced references to "atherosclerosis" with "cIMT" throughout the text, including the title, abstract, and main body.

We have emphasized the role of cIMT as a surrogate endpoint:

Example from the revised Introduction paragraph, lines 38-40:

"*Carotid intima-media thickness (cIMT), measured non-invasively by ultrasound, serves as a surrogate marker of vascular health and is associated with increased cardiovascular risk.*[2-4]"

Also, we have expanded the Limitations paragraph to further emphasize this distinction, lines 121-122:

"*Additionally, cIMT is a surrogate endpoint regarding vascular health.*"

In the Results section, we have amended the sentence on associated cardiovascular risk, lines 66-69:

From: "*This effect size approximates to an average decrease of 0.04 mm, which is of clinical interest, as meta-analysis has estimated that a decrease of 0.04 mm/year corresponds to a 37% relative reduction in CVD risk.*[2]"

To: *"The observed absolute change in cIMT (~0.04 mm) in this study is within the range reported in meta-analysis to be associated with cardiovascular risk reduction; however, whether such change indeed translates into clinical benefit remains uncertain.*[2]*"*

4) Please expand the strengths and limitations section of the manuscript to ensure a balanced discussion. This applies to design limitations, absence of baseline measurements, possible confounding factors, and generalizability of the findings to other GLP-1R agonists. Follow recommendations of all 3 Reviewers in this regard.

Author's response:

We have carefully incorporated the specific recommendations from the three reviewers when revising this paragraph.

Revised Strength and Limitation paragraph, lines 114-134:

"*Strengths of this analysis include standardization of diet and weight loss via an LCD run-in, the randomized four-group design enabling evaluation of both independent and combined interventions, high adherence to exercise and study medication, and comprehensive profiling using minimally invasive vascular imaging and emerging pro-atherosclerotic biomarkers. Limitations should be considered. This is a prespecified secondary analysis, and findings should be interpreted with appropriate caution regarding type I error. The ultrasound assessments were performed by personnel trained under a protocol that yielded a low coefficient of variation (around 5%) prior to the initiation of the trial. Additionally, cIMT is a surrogate endpoint regarding vascular health.[2] While liraglutide is a first-generation GLP-1 RA with modest weight loss effects compared to newer agents,[5] liraglutide was administered at the highest approved dose and maintained a large weight loss, enabling us to elucidate mechanistic effects of GLP-1 RA with or without exercise. Participants in the exercise groups had more frequent contact with study personnel than those in the placebo and liraglutide groups, which could have led to a Hawthorne effect; nevertheless, adherence to study medication was similar across all groups. The partly supervised exercise intervention helped ensure adequate intensity and volume for mechanistic evaluation but may limit direct generalizability to routine clinical settings. The intervention had a duration of one year, and it is uncertain whether the observed results are sustainable in the long term; although, a follow-up study of the S-LiTE trial showed that the exercising groups had better maintenance of body weight and body composition one year after termination of the supervised exercise program, whereas weight gain was observed after cessation of liraglutide treatment alone.[6]*"

Regarding baseline measurements, these are presented in the revised Table 1 and included in the statistical analysis, as mentioned by Reviewer #1, point 3.

5) Please ensure full transparency and adherence to reporting standards for secondary analyses. Follow recommendations of Reviewer #2 in this regard.

<u>Author's response:</u>

We have carefully followed the recommendations of Reviewer #2 and revised the manuscript accordingly. We have addressed reporting standards for secondary analyses, including updating the title and abstract to clearly identify the work as a prespecified secondary analysis; adding a CONSORT checklist and flowchart; expanding the Methods section with details on randomization, blinding, covariates, and missing data handling; including the protocol and statistical analysis plan as supplementary material; and acknowledging type I error risk as a limitation.

Please see our full responses to the specific recommendations raised by the reviewer below.

6) Editorially, we ask you to consider one of the title suggestions below, to ensure more accuracy in endpoint and secondary analysis reporting:
"Exercise but not liraglutide alone reduces carotid intima media thickness during weight loss maintenance: a prespecified secondary analysis of the S-LITE trial" (if the four-group analyses supports this conclusion)
Or
"Effects of exercise and liraglutide alone or in combination on carotid intima media thickness during weight loss maintenance: a prespecified secondary analysis of the S-LITE trial"

Authors' response:

Thank you for the suggestions. Based on your first title suggestion and our findings of the four-group analysis, we propose this title:

"*Exercise but not liraglutide alone reduces carotid intima-media thickness and inflammation during weight loss maintenance: a prespecified secondary analysis of the S-LiTE trial*"

7) Editorially, we ask you to reformat the manuscript into a Brief Communication. Brief Communications contain up to 1,500 words and 2 Main Display Items, and can be accompanied by Extended Data figures and Supplementary information. As stated above, we ask you to reserve the Main Display Items and Extended Data Items for the four-group design, and present the 2x2 design as Supplementary Data. To find out more about our content types, please see https://www.nature.com/natmetab/content

Although the above points are the most important to us, please do also respond to the other points raised by our referees, ideally with new experiments. If you have any questions or would like to discuss the scope of the revision please do get in touch.

In the event of resubmission, please include a point-by-point response to the referees' comments (in a separate document to any cover letter). At the top of your point-by-point response to the referees' comments, please also include responses to the various editorial points highlighted above as guidance for your revision (this section should be labelled "Response to editors' comments").

Nature journals have recently announced an update to our guidance on reporting on sex and gender in research studies (see here). We strongly encourage

researchers to follow the 'Sex and Gender Equity in Research – SAGER – guidelines' and to include sex and gender considerations for studies involving humans, vertebrate animals and cell lines where relevant to the topic of study (an overview can be found here). Authors should use the terms sex (biological attribute) and gender (shaped by social and cultural circumstances) carefully in order to avoid confusing both terms.

When preparing your revised manuscript, please be aware of our guidance on Sex and Gender reporting). Please note that:

1. If the research findings apply to only one sex or gender, that must be indicated in the title and/or abstract.

Authors' response:

Not applicable. Compared with other obesity trials, which often include predominantly females (>70%),[7,8] we had a more balanced representation of sex (assigned at birth), with approximately 40% male and 60% female participants.

2a. For studies involving vertebrates animal and cell lines- The Reporting Summary should include whether sex was were considered in the study design.

Authors' response:

Not applicable.

2b. For studies involving human research participants- The Reporting Summary should include whether sex and/or gender was considered in the study design and whether sex and/or gender of participants was determined based on self-report or assigned (and methodology used).

Authors' response:

In the Reporting Summary, we have included that sex was considered in the design and recruitment, as obesity trials historically have recruited more female than male participants.[7,8] In our trial, we actively sought a balanced recruitment of sexes. Sex was defined as assigned at birth and was registered by a physician in an electronic case report form.

3. Data should be reported disaggregated for sex and gender where this information has been collected and consent has been obtained for reporting and sharing individual-level data; disaggregated numbers for individual experiments must be provided in the source data as appropriate whereas overall numbers may be provided in the Nature Portfolio Reporting Summary.

Information on the 3 points above should be included in the revised manuscript and detailed in the cover letter.

In addition, please note that if sex- and gender-based analyses have been performed a priori, results should be reported regardless of positive or negative outcome. We discourage conducting post hoc sex- and gender-based analysis if the study design is insufficient (for example, low sample size) to enable meaningful conclusions.

If no sex- and gender-based analyses have been performed, please indicate the reasons for the lack of these analyses in the Reporting Summary.

Authors' response:

Given the small sample sizes when disaggregating sex within the four-arm groups, we did not conduct post hoc sex-specific analyses, as the study was not adequately powered to draw meaningful conclusions from such comparisons. However, sex (assigned at birth) was an inclusion stratum in our trial, and we have accordingly included sex as a covariate in our mixed-effects model to adjust for potential differences between male and female participants in the primary analysis. We have also provided the sex distribution of participants in the baseline characteristics table (the revised Table 1), as requested by a reviewer.

I should stress, however, that we would be reluctant to trouble our referees again unless we thought their comments had been addressed in full. In the meantime we hope that you find our referees' comments helpful.

Best regards,

Jean Nakhle
Editor
Nature Metabolism

Reviewers' expertise:

Reviewer #1: GLP-1R agonists, cardiovascular outcomes
Reviewer #2: Clinical statistics
Reviewer #3: Exercise, cardiovascular outcomes

Authors' responses to reviewers' comments:

Reviewers' comments:

Reviewer #1 (Remarks to the Author):

In this manuscript, Sandsdal and colleagues present the findings of a study of individuals with obesity and without diabetes, who underwent a low calorie diet for 8 weeks with weight loss and then were randomized in a two-by-two factorial design to the presence or absence of exercise and the GLP-1 receptor agonist liraglutide. They report changes in carotid IMT, as a surrogate measure of atherosclerosis, and report differences – in particular a reduction in cIMT at 52 weeks in the exercise group, but not those treated with liraglutide. This is complemented by favorable effects on systemic biomarkers associated with atherosclerosis. The findings are interesting, although in its current form there is considerable ambiguity of the representation of the study and results. The authors should consider a number of points.

1. The data is presented in a form that is not consistent with the reported methodology. If I've read this correctly, there was a 2x2 design – in other words, half the patients received the exercise intervention and half did not, similarly half received the GLP-1 receptor agonist and the other half did not. The results present exercise and liraglutide as completely separate groups, which is not correct as one quarter of participants must have received both. The findings should be presented more accurately.

2. On the basis of this, I can't make any further assessment of the findings of this study.

Authors' response:

We thank Reviewer #1 for taking the time to review our manuscript and for providing constructive feedback. The reviewer is correct that our study employed a factorial design, and we agree that our original presentation of the data did not clearly reflect this structure. As requested, we have revised the manuscript to present outcomes for all four individual randomized groups (placebo, exercise alone, liraglutide alone, and exercise + liraglutide). Please note that we have reformatted the manuscript to fit a "Brief Communication" for the journal, as requested by the Editor.

We have revised the manuscript accordingly:

- The manuscript now presents outcomes for all four randomized intervention groups separately (i.e., placebo, exercise, liraglutide, and exercise + liraglutide).
- All primary inferences in this revised manuscript are based on the four-group parallel randomized comparisons. The 2x2 factorial analyses have been moved to Supplementary Data.

- Data visualizations have been remade into a Main Display Item, and tables have been updated to show the four intervention groups individually.

The analysis of the individual intervention groups shows that after a diet-induced weight loss, standardizing nutritional and metabolic status, exercising participants were able to improve their carotid intima-media thickness and inflammation levels in a weight-loss-independent manner, while GLP-1 RA treatment alone showed no such improvements despite a similarly maintained weight loss. These findings show that the cardiovascular benefits of exercise can extend beyond weight reduction, whereas the effects of GLP-1-based therapy on vascular health may be predominantly mediated by weight loss.

3. Beyond, this there appears to be no control for baseline measurements, which is critical in serial imaging studies. While the authors have applied a linear model, accounting for time, baseline cIMT does not appear to be a covariate that has been accounted for.

<u>Authors' response:</u>

We agree and have revised the model to include baseline cIMT (at randomization) as a covariate. The results of this analysis show that the exercising participants (exercise and combination treatment) reduced cIMT by a similar magnitude, supporting the robustness of our conclusions. This analysis is now reported in the Main Display Item 1, and Results, Discussion, and Conclusion paragraphs in the revised manuscript.

We have revised the statistical analysis in the Methods section, lines 267-271:

"*Changes in outcomes were analyzed using a linear mixed model with time (factorial), treatment group (placebo, exercise, liraglutide, or exercise + liraglutide), time-treatment interactions, age (<40 years or ≥40 years), and sex as fixed effects. For serial imaging, the baseline value (at randomization) was included as a covariate. The model assumed an unstructured covariance and a repeated effect for time.*"

We present the results of the analysis of baseline-adjusted cIMT in the Results paragraph, lines 62-65:

"*No changes in cIMT were observed during the LCD-induced weight loss (Supplementary Table S1). During the 52-week weight maintenance phase, cIMT decreased in the exercise-containing arms; exercise group -7% (95% CI: -12 to -2), p=0.01, and the combination group -6% (-11 to -1), p=0.02 (Main Display Item 1, Supplementary Table S3). […] No changes were observed with liraglutide treatment alone.*"

4. It is also intriguing that the only data presented is change, there is no baseline data. The reviewer cannot be convinced that there were not differences between the groups that may have accounted for differences in changes in cIMT.

Authors' response:

We have revised the baseline characteristics table to present data for all four intervention groups at the time of randomization (after completion of the low-calorie diet phase), please see Table 1. Furthermore, as described above, we have included baseline cIMT as a covariate in our linear mixed model analysis of change in cIMT to directly account for any potential baseline differences. Changes during the LCD have been relocated to Supplementary Table S1.

5. The authors should remove the references in the manuscript and figures suggesting potential reductions in clinical risk with the cIMT changes.

Authors' response:

We acknowledge that the original manuscript overstated the potential effects of the reduced cIMT on cardiovascular risk. Thus, we have removed the reference from Figure 1 (now Main Display Item 1).

In the Results section, we have amended the sentence on associated cardiovascular risk, lines 66-69:

From: "*This effect size approximates to an average decrease of 0.04 mm, which is of clinical interest, as meta-analysis has estimated that a decrease of 0.04 mm/year corresponds to a 37% relative reduction in CVD risk.[2]*"

To: *"The observed absolute change in cIMT (~0.04 mm) in this study is within the range reported in meta-analysis to be associated with cardiovascular risk reduction; however, whether such change indeed translates into clinical benefit remains uncertain.[2]"*

Also, we now state in the Limitations paragraph, line 121-122:

"*Additionally, cIMT is a surrogate endpoint regarding vascular health.*[2]"

6. There is no other data given on medications.

Authors' response:

We thank the reviewer for raising this point, as medication status, particularly blood pressure and lipid-lowering drugs, is important in the context of investigations into vascular health. In response, we have added the information to Supplementary Table S6, which shows the use of antihypertensive and lipid-lowering medications.

7. The authors should acknowledge that cIMT is not the earliest feature of atherosclerosis. There is no literature, whatsoever, that demonstrates that cIMT appears first and then develops into plaque. It is simply a barometer of vascular health.

Authors' response:

We agree with the reviewer that this should be clarified. We have revised the manuscript accordingly, now describing cIMT as a marker of vascular health rather than a stage of atherosclerotic disease and eventually plaques. This distinction is now reflected throughout the manuscript, including:

the Introduction, lines 38-40:

"*Carotid intima-media thickness (cIMT), measured non-invasively by ultrasound, serves as a surrogate marker of vascular health and is associated with increased cardiovascular risk.*[2-4]"

the Results paragraph, lines 60-62:

"*To assess the effects of exercise and liraglutide on vascular health, we quantified intimal thickening in the carotid artery by measuring the intima-media thickness using standardized ultrasound.*[9,10]"

… and the Limitations paragraph, line 121-122:

"*Additionally, cIMT is a surrogate endpoint regarding vascular health.*[2]"

Additionally, as requested by the Editor, we have replaced references to "atherosclerosis" with "cIMT" throughout the manuscript and revised the title to better reflect this point.

From: "*Exercise but not GLP-1-based therapy reduces early development of atherosclerosis during weight loss maintenance: a randomized controlled trial*"

To: "*Exercise but not liraglutide alone reduces carotid intima-media thickness and inflammation during weight loss maintenance: a prespecified secondary analysis of the S-LiTE trial*"

8. The lack of correlation between blood pressure and cIMT is problematic, it is particularly sensitive to blood pressure.

Authors' response:

We agree that cIMT is known to be sensitive to blood pressure. We have now tested for correlations between changes in blood pressure and cIMT from randomization through the end of the trial and found no significant association. At inclusion (before

the low-calorie diet), cIMT and systolic blood pressure were positively correlated. During the low-calorie diet, blood pressure dropped substantially (around 10 mmHg) and normalized to a mean of 122 mmHg. In the follow-up period, blood pressure did not change significantly across the groups after one year of treatment, which may explain the absence of a correlation, given the limited variability in blood pressure changes.

We have added these findings to the Results paragraph, lines 90-92, and reported the association analysis of blood pressure in Supplementary Table S4, including biomarkers, as requested by another reviewer:

"At inclusion, cIMT was positively associated with blood pressure, but changes in cIMT during the one-year treatment were not associated with changes in blood pressure or biomarkers (Supplementary Table S4)."

Supplementary Table S4. Association of change in biomarkers with change in cIMT

Parameter	***Unstandardized β***
Tumor necrosis factor-alpha (TNF-alpha)	*-0.020 (-0.10 to 0.06)*
Interferon-gamma (IFN-gamma)	*0.004 (-0.02 to 0.03)*
Interleukin 6 (IL-6)	*0.02 (-0.02 to 0.06)*
Interleukin 8 (IL-8)	*-0.06 (-0.13 to 0.01)*
Interleukin 10 (IL-10)	*0.02 (-0.02 to 0.07)*
Von Willebrand factor (vWF)	*0.11 (-0.02 to 0.24)*
Tissue plasminogen activator (tPA)	*-0.002 (-0.08 to 0.07)*
Soluble Intercellular adhesion molecule 1 (ICAM-1)	*0.03 (-0.17 to 0.24)*
Soluble Vascular adhesion molecule 1 (VCAM-1)	*0.09 (-0.03 to 0.22)*
Systolic blood pressure	*-0.0005 (-0.002 to 0.001)*
Diastolic blood pressure	*-0.001 (-0.004 to 0.001)*

Association of change from baseline (week 0) to end-of-treatment (week 52) in outcomes (independent variable) with change from baseline (week 0) to end-of-treatment (week 52) in carotid intima-media thickness (cIMT, as dependent variable). Changes in cIMT and biomarkers are on a log scale. The analyses are adjusted for sex, age (above/below 40 years), and intervention group.

9. There is no data provided on variability between reading assessments.

Authors' response:

Thank you for this comment. The cIMT was measured by trained personnel, and in accordance with the principles stated here,[10] which yielded a low coefficient of variation (around 5%) prior to the initiation of the trial.

We have now included this in the Methods section, lines 241-242:

"Assessments were performed by trained personnel and in accordance with the method described in detail by Potter et al.[10] which provides a coefficient of variation of 5.1%."

We have now included a table with coefficient of variation, limit of detection, and limit of quantification of each biomarker in *Supplementary Table S7:*

Variable	***Inter-assay CV (%)***	***LLOD***	***LLOQ***
IL-6 (pg/mL)[a]	*7.3*	*0.06*	*0.633*
IL-8 (pg/mL) [a]	*7.1*	*0.07*	*0.591*
IL-10 (pg/mL) [a]	*10.1*	*0.04*	*0.298*
IFN-gamma (pg/mL) [a]	*7.3*	*0.37*	*1.76*
TNF-alfa (pg/mL) [a]	*10.1*	*0.04*	*0.690*
sVCAM (pg/mL) [a]	*8.4*	*6.00*	*37.6*
sICAM (pg/mL) [a]	*13.4*	*1.94*	*15.0*
tPA (ng/mL)	*5*	*0.31*	*0.75*
vWF (%)	*6*	*3.7*	*12.4*

Inter-assay coefficient of variation in percent (CV) is the variability across different plates (usually 96-well plates) used for analyzing various biomarkers. LLOD: Lower limit of detection is defined as the calculated concentration corresponding to the average signal 2.5 standard deviations above background signaling. LLOQ: Lower limit of quantification: For [a] is defined as the lowest concentration (of the control samples) where the CVs of the calculated concentration were below 20% and where recovery of the samples was within 80-120% of the known value of the standard across all used plates. For tPA and vWF, it was defined as the average signal 10 standard deviations above background signaling. [a]Numbers provided by the manufacturer of the kits (MSD).

10. It should be acknowledged that liraglutide is a relatively modest GLP-1 receptor agonist and the findings do not necessarily imply what may be encountered with more commonly used GLP-1 receptor agonists.

Authors' response:

We concur that liraglutide is indeed a first-generation GLP-1 receptor agonist, and newer drugs such as semaglutide and tirzepatide produce greater weight loss. However, a notable strength of our study is that all participants had already achieved a standardized weight loss of approximately 12% through the low-calorie diet phase prior to randomization, which liraglutide effectively maintained during the intervention

period. This design can allow weight-independent interpretations of the effects of liraglutide on the outcomes.

We added the following to the Limitations paragraph, lines 122-125:

"While liraglutide is a first-generation GLP-1 RA with modest weight loss effects compared to newer agents,[5] liraglutide was administered at the highest approved dose and maintained a large weight loss, enabling us to elucidate mechanistic effects of GLP-1 RA with or without exercise."

11. The study design is odd. Everyone underwent weight loss and then the interventions were applied, yet that is not how this would play out in clinical practice – the interventions are used to promote (and then hopefully maintain) the weight loss.

Authors' response:

The rationale for implementing a standardized low-calorie diet phase prior to randomization was to ensure that all participants entered the intervention period in a comparable metabolic and nutritional state, having achieved equivalent weight loss. This design enables mechanistic insights into the individual contributions of these interventions beyond their weight-reducing effects. Notably, our finding that exercise improved cIMT in a weight-stable context suggests that its vascular benefits are at least partly independent of weight loss, which we believe has clinical implications for weight loss maintenance strategies.

We have elaborated on rationale and study design in the revised Introduction, lines 40-46:

"*Both exercise and glucagon-like peptide-1 receptor agonists (GLP-1 RAs) individually seem to promote cardiovascular health.[5,11-13] However, mechanisms by which exercise and GLP-1 RAs ameliorate cardiovascular risk after weight loss remain to be determined. Therefore, we investigated changes in cIMT and circulating inflammatory and endothelial biomarkers in a prespecified secondary analysis of the S-LiTE randomized placebo-controlled trial[14] to elucidate distinct mechanisms on vascular health of exercise, GLP-1 RA, and the combination treatment.*"

… and lines 47-53:

"*To standardize nutritional and metabolic status at randomization and reduce variation in circulating biomarkers attributable to dietary factors alone, participants in the S-LiTE trial had to complete an 8-week LCD to lose at least 5% of their body weight. In total, 130 adults with obesity (BMI 32–43 kg/m^2) without diabetes completed the LCD and lost an average of 13.7 kg (12.7%) of total body weight and were randomized to 52 weeks of treatment with placebo, exercise, liraglutide 3.0 mg/day, or exercise combined with liraglutide (Supplementary Figure S1 and Table S1).*"

Reviewer #2 (Remarks to the Author):

I thank the authors for their manuscript. I offer the following methodological comments

Authors' response:

We thank the reviewer for their time and constructive comments on our manuscript. Below follows our responses to each of the bullets.

1) Secondary analysis
i) Ensure that this is clear in the title and abstract that readers are aware this is a secondary analysis of an RCT - at present this is not clear

Authors' response: We have made clear in the title and the abstract that this is a secondary analysis of the randomized controlled S-LiTE trial

From: "*Exercise but not GLP-1-based therapy reduces early development of atherosclerosis during weight loss maintenance: a randomized controlled trial*"

To: "*Exercise but not liraglutide alone reduces carotid intima-media thickness and inflammation during weight loss maintenance: a prespecified secondary analysis of the S-LiTE trial"*

Specification in the abstract, lines 26-27:

"In this secondary analysis, 130 adults with obesity completed a diet-induced weight loss followed by randomization to weight maintenance with exercise and/or liraglutide for 52 weeks."

ii) Include the CONSORT checklist and Flowchart that is relevant for this study

Author's response: We have included the CONSORT checklist (attached to this revision) and flowchart in the submission, please see Supplementary Figure S1.

iii) Include the primary study sample size

Authors' response: We now describe in the Methods section how the primary study sample size was estimated, lines 284-286:

"In the original trial, it was estimated that 30 participants in each group needed to complete the study to detect a 4 kg difference in weight between the intervention groups with a power of 80% at a significance level of 5%.[14]"

iv) Include the method of sequence generation

Authors' response: The method of sequence generation is now described in the Methods section in detail, lines 174-185:

"On completion of the 8-week low-calorie diet, only participants who lost at least 5% of their initial body weight were eligible for enrolment and subsequently randomized in this study. Participants were randomly allocated to one of four 52-week interventions: placebo with usual activity, exercise with placebo, liraglutide with usual activity, or a combination of exercise and liraglutide, in a 1:1:1:1 ratio to one of four treatment arms using a computer-generated randomization sequence provided by Novo Nordisk A/S (subject randomization list), see study flow, Supplementary Figure S1. The allocation sequence was stratified by sex and age, with separate randomization lists for participants aged ≥40 years and <40 years. Within each stratum, participants were assigned sequentially according to the randomization list. Allocation was implemented by a qualified, unblinded study nurse who was not otherwise involved in trial conduct. Study participants and study personnel were blinded to the study medication."

v) Introduction - state why this is an important question and how this was different to the primary publication

Authors' response: We agree that justification for the secondary analysis is needed, and we now include this in the revised Introduction, lines 40-46:

"*Both exercise and glucagon-like peptide-1 receptor agonists (GLP-1 RAs) individually seem to promote cardiovascular health.*[5,11-13] *However, mechanisms by which exercise and GLP-1 RAs ameliorate cardiovascular risk after weight loss remain to be determined. Therefore, we investigated changes in cIMT and circulating inflammatory and endothelial biomarkers in a prespecified secondary analysis of the S-LiTE randomized placebo-controlled trial*[14] *to elucidate distinct mechanisms on vascular health of exercise, GLP-1 RA, and the combination treatment."*

2) Methods

i) State the method for handling the missing data

Authors' response: Missing data were handled implicitly in the linear mixed model and were assumed to be missing at random.

We now write in the Methods section, lines 282-283:

"*All missing data were assumed to be missing at random and handled implicitly in the mixed model by maximum likelihood estimation."*

ii) State the analysis model in full and justify the included covariates and compare this with the primary publication.

Authors' response: We agree that the analysis model should be included in full, and that the justification for the included covariates should be provided, together with a comparison to the primary publication.

We now state in the Methods section, lines 267-273:

"Changes in outcomes were analyzed using a linear mixed model with time (factorial), treatment group (placebo, exercise, liraglutide, or exercise + liraglutide), time-treatment interactions, age (<40 years or ≥40 years), and sex as fixed effects, as prespecified in the statistical analysis plan for the original trial.[14] For serial imaging, the baseline value (at randomization) was included as a covariate. Participants were stratified on age and sex and, therefore, included as covariates in the model as recommended by European Medicines Agency."

iii) Outcomes : list these clearly

Authors' response: We have revised the Methods section and listed the investigated outcomes accordingly, from lines 227-264.

iv) Blinding status of personnel - state clearly

Authors' response: Regarding the blinding status of the personnel, we now clearly state this procedure in the Methods section, lines 183-185:

"Allocation was implemented by a qualified, unblinded study nurse who was not otherwise involved in trial conduct. Study participants and study personnel were blinded to the study medication."

Regarding blinding of the analysis of the outcomes, the Methods section, lines 245-248, now states:

"*The right common carotid artery cIMT was measured by two blinded assessors at three evenly spaced locations on the image frame of the far carotid artery wall obtained at the R wave of the accompanying ECG recording and reported as the average of these values. Blinded analyses were performed using ultrasound quantification software (QLAB, Philips).*"

3) Discussion
i) State how these findings fit with the primary publication

Authors' response: We now discuss the improved cardiometabolic risk factors observed in the exercise groups (exercise + placebo and exercise + liraglutide), compared with the minimal improvements observed with liraglutide. These findings should be interpreted alongside reference to the primary publication's results, which show that weight loss was maintained in both the exercise and liraglutide groups.

We have elaborated this in the Discussion paragraph, lines 103, 93-99, and 130-134:

"Liraglutide maintained the LCD-induced weight loss[14] but did not reduce cIMT in this study."

"*Our findings underscore the importance of incorporating and adhering to physical activity as an adjunct to GLP-1-based therapy for obesity, as it promotes healthy weight maintenance with reduced levels of pro-inflammatory cytokines IL-6 and IFN-γ and endothelial function markers sICAM-1, sVCAM-1, and tPA. Together with the observed decrease in cIMT captured by ultrasound, our findings suggest that structural vascular adaptations are more sensitive to exercise-induced hemodynamic and anti-inflammatory effects than to liraglutide's effects on metabolism alone during weight loss maintenance.[14]*"

"*The intervention had a duration of one year, and it is uncertain whether the observed results are sustainable in the long term; although, a follow-up study of the S-LiTE trial showed that the exercising groups had better maintenance of body weight and body composition one year after termination of the supervised exercise program, whereas weight gain was observed after cessation of liraglutide treatment alone.[6]*"

ii) Weaknesses - type-1 error due to a secondary publication

Regarding the risk of type 1 error, we have included this in the Limitation paragraph, lines 118-119:

"This is a prespecified secondary analysis, and findings should be interpreted with appropriate caution regarding type I error."

4) Protocol and SAP
i) Include both as supplementary material with version number and dates

Authors' response: We have included the latest version, with dates for both the protocol and the SAP.

5) Output
i) Table 1 - remove the between group comparison and include all 4 randomised groups with subtotals for the key comparison here
ii) Figure 1 - show both factors on each bar - improve the quality of the figure.

Authors' response: We have revised Table 1 and now present all four randomized groups. We have also revised Figure 1 to include all outcomes in the Forrest plot, please see the figure at the end of the revised manuscript.

Reviewer #3 (Remarks to the Author):

Many thanks for asking me to review this article. The authors should be congratulated in conducting this exploratory study with such attention to detail and achieving high levels of follow up. There are a number of things that should be altered or included to improve the scientific validity of this study.

Authors' response:

We thank the reviewer for taking the time to review our manuscript and for the kind words about our study. We appreciate the constructive suggestions and have carefully addressed each point raised in the revised manuscript. Please see responses below.

The purpose of this study is to determine the effect of liraglutide and exercise on imaging and circulating biomarkers of cardiovascular disease. The primary outcome of this study was change in the intima-media thickness of the carotid artery.

Firstly, the title "Exercise but not GLP-1-based therapy reduces early development of atherosclerosis during weight loss maintenance: a randomized controlled trial" is misleading. The study demonstrated that exercise reduced carotid intimal media thickness which is associated with a reduction in cardiovascular events. The reviewer feels the title should be tempered. The title implied that this study is comparing exercise with GLP-1 in the development of atherosclerosis but actually this has not been evaluated.

Authors' response:

We agree and have clarified in the title that it pertains to liraglutide treatment alone, and we have added that this is a prespecified secondary analysis of the randomized controlled S-LiTE trial. We have also omitted the term "atherosclerosis" throughout the manuscript and replaced it with "carotid-intima media thickness".

From: "*Exercise but not GLP-1-based therapy reduces early development of atherosclerosis during weight loss maintenance: a randomized controlled trial*"

To: "*Exercise but not liraglutide alone reduces carotid intima-media thickness and inflammation during weight loss maintenance: a prespecified secondary analysis of the S-LiTE trial"*

The abstract, lines 26-27, now states:

"In this secondary analysis, 130 adults with obesity completed a diet-induced weight loss followed by randomization to weight maintenance with exercise and/or liraglutide for 52 weeks."

There is no mention of plaque volume in this evaluation.

Authors' response:

Plaque volume was not directly assessed in our study. Only one participant was incidentally found to have manifest plaque during the assessment and was referred to the cardiology department at site for further evaluation. Our imaging protocol focused on cIMT as a surrogate marker of early vascular adaptations, which is appropriate for our relatively young study population with obesity but without established cardiovascular disease.[15]

Results
The results are impressive even before randomisation. The reviewer would suggest that in the results section the final weight loss could be stated in parenthesis next to the subsequent weight loss or gain in each arm.

Authors' response:

We agree that this would improve understanding of the results and have presented the total weight losses in a Supplementary Table S2.

We now write in the Results paragraph, lines 53-57:

"After one year, the placebo group regained weight, and the combination further reduced body weight, while the exercise and liraglutide groups maintained the LCD-induced weight loss. This enabled us to investigate weight-loss-independent effects of exercise or liraglutide treatment alone. The total weight loss from the start to end of the trial is presented in Supplementary Table S2."

Was there any correlation with reduction in cIMT and reduction in markers of inflammation? If so this could be a helpful visual in the supplement for the reader and give more credit to the discussion.

Authors' response:

We agree that examining associations between changes in cIMT and changes in inflammatory markers would be of interest. We have performed these analyses; however, no significant associations were observed between changes in circulating biomarkers and changes in cIMT. This may reflect the relatively small sample size and homogeneous study population, which limits statistical power to detect such associations. As requested by another reviewer, we also tested the association between changes in cIMT and blood pressure. The results are now reported in the Supplementary Table S4.

We now state in the Results paragraph, lines 90-92:

"*At inclusion, cIMT was positively associated with blood pressure, but changes in cIMT during the one-year treatment were not associated with changes in blood pressure or biomarkers (Supplementary Table S4)."*

Discussion
“In this randomized, controlled trial, we evaluated the impact of exercise and GLP-1-based therapy on the early development of atherosclerosis and vascular inflammation during weight loss maintenance in adults with obesity without diabetes”. The reviewer would suggest changing the early development of atherosclerosis to carotid intimal-media thickness.

Authors’ response:

We have revised the manuscript accordingly. We have replaced references to "atherosclerosis" with "cIMT" throughout the text, including the title, abstract, and main body.

The study asked all participants to eat a calorie deficient diet prior to being randomised. The reviewer feels that the rationale for this should be explained. It is clearly a strength of the study as it helps to standardise participants nutritional status at baseline and reduce any variation in circulating biomarkers that could be attributed to dietary factors alone.

Authors’ response:

Thank you for raising this point. We have now elaborated on the study design in the revised Introduction, lines 47-53:

“*To standardize nutritional and metabolic status at randomization and reduce variation in circulating biomarkers attributable to dietary factors alone, participants in the S-LiTE trial had to complete an 8-week LCD to lose at least 5% of their body weight. In total, 130 adults with obesity (BMI 32–43 kg/m^2) without diabetes completed the LCD and lost an average of 13.7 kg (12.7%) of total body weight and were randomized to 52 weeks of treatment with placebo, exercise, liraglutide 3.0 mg/day, or exercise combined with liraglutide (Supplementary Figure S1 and Table S1).”*

and in the Strengths paragraph, line 114:

“[…] *standardization of diet and weight loss via an LCD run-in*,”

The discussion section makes little in the way to explain whether this neutral effect of liraglutide on biomarkers and cIMT is drug specific or a class effect of GLP1. The reviewer feels this is an important aspect to address given the global use and demand for other GLP-1 agents.
Weight independent effects vs. lira

Authors’ response:

This is a very relevant comment. We have elaborated this in the Discussion paragraph, lines 103-113:

"Liraglutide maintained the LCD-induced weight loss[14] but did not reduce cIMT in this study. This contrasts with previous clinical trials showing cIMT reduction with GLP-1 RA treatment; however, those studies examined patients with type 2 diabetes, a population characterized by substantially higher baseline inflammation and cardiovascular risk, and did not include an initial standardized weight loss phase.[16-18] In the present study, all participants achieved approximately 13% weight loss through the LCD before randomization, which liraglutide then maintained throughout the intervention period. This design allows for assessment of GLP-1 RA effects independent of ongoing weight change. Our findings indicate that in people with obesity but without diabetes and with no established cardiovascular disease who have already achieved substantial weight loss, liraglutide provides limited additional benefit for reducing cardiovascular risk markers unless combined with exercise."

We have also added the following to the limitations paragraph, lines 122-125:

"While liraglutide is a first-generation GLP-1 RA with modest weight loss effects compared to newer agents,[5] liraglutide was administered at the highest approved dose and maintained a large weight loss, enabling us to elucidate mechanistic effects of GLP-1 RA with or without exercise."

Strengths and limitations
The reviewer feels that the section needs more thought.

To me the strengths are;
- Comprehensive follow up
- Enrollment of only those that manages to lose >5% of their body mass
- Standardisation of diet in the lead up to randomisation
- Inclusion of a number of emerging circulating biomarkers

Authors' response:

Thank you for these comments. We agree that our study has these strengths, and we now mention this in the Strength paragraph in the discussion, lines 114-117:

"Strengths of this analysis include standardization of diet and weight loss via an LCD run-in, the randomized four-group design enabling evaluation of both independent and combined interventions, high adherence to exercise and study medication, and comprehensive profiling using minimally invasive vascular imaging and emerging pro-atherosclerotic biomarkers."

Limitations
Relatively short follow up and whether these results are sustainable in the longer term is uncertain

Authors' response:

We agree that a one-year follow-up period isn't very long-term. However, we conducted a one-year post-treatment follow-up (equivalent to two years of total follow-up), which shows that the exercise groups maintained weight loss better than the liraglutide group, indicating longer-lasting effects of the exercise intervention.

We have added this to the Limitations paragraph, lines 130-134:

"The intervention had a duration of one year, and it is uncertain whether the observed results are sustainable in the long term; although, a follow-up study of the S-LiTE trial showed that the exercising groups had better maintenance of body weight and body composition one year after termination of the supervised exercise program, whereas weight gain was observed after cessation of liraglutide treatment alone.[6]"

The impact of these results on clinical outcomes is unknown

Authors' response:

We agree that the impact of the results on clinical outcomes is only indicative, and we now mention this in the Limitations paragraph, line 121-122:

"*Additionally, cIMT is a surrogate endpoint regarding vascular health.[2]"*

There was intensive input to deliver the exercise intervention and this has important implications for the generalisability of the findings of this study into routine clinical practice.

Authors' response:

We thank the reviewer for raising this point. While the partly supervised nature of the exercise intervention may limit direct translation into current routine clinical practice, this approach was important to ensure adequate intensity and volume to test the mechanistic hypothesis.

In the Limitations paragraph, lines 128-130, we have added:

"The partly supervised exercise intervention helped ensure adequate intensity and volume for mechanistic evaluations but may limit direct generalizability to routine clinical settings."

Reproducibility of doppler USS

Authors' response:

Thank you for this comment. The cIMT was measured by trained personnel, and in accordance with the principles stated here,[10] which yielded a low coefficient of variation (around 5%) prior to the initiation of the trial. We have now included this in the

Methods section as well as stated the lack of reproducibility as a limitation in the discussion.

Added to Limitations paragraph, line 119-121:

"The ultrasound assessments were performed by personnel trained under a protocol that yielded a low coefficient of variation (around 5%) prior to the initiation of the trial."

Added to Methods section, lines 241-242:

"Assessments were performed by trained personnel and in accordance with the method described in detail by Potter et al.[10] which provides a coefficient of variation of 5.1%."

Methodology
Definition of obesity
The authors state a BMI 32-43kg/m2 as opposed to >30kg/m2. Why was this higher threshold selected and why was there an upper limit to this?

Authors' response:

Thank you for your comment. The lower limit of 32 was chosen because we expected that the low-calorie diet would lead to substantial weight loss, potentially causing participants to reach BMI levels below 30 (or even 25) and no longer be classified as at least having overweight or obesity by BMI standards before initiating GLP-1 RA treatment. The upper limit was set to allow participants to engage in the exercise program without too many limitations.

Timing of randomisation
The timing of randomisation should be more explicit. Suggest something like "On completion of an 8-week low calorie diet, only participants who lost at least 5% of their initial body weight were eligible for enrolment and subsequently randomised in this study".

Authors' response:

Thank you for pointing this out. We now write the timing of randomization more explicitly in the Results paragraph, lines 47-53:

"*To standardize nutritional and metabolic status at randomization and reduce variation in circulating biomarkers attributable to dietary factors alone, participants in the S-LiTE trial had to complete an 8-week LCD to lose at least 5% of their body weight. In total, 130 adults with obesity (BMI 32–43 kg/m^2) without diabetes completed the LCD and lost an average of 13.7 kg (12.7%) of total body weight and were randomized to 52 weeks of treatment with placebo, exercise, liraglutide 3.0 mg/day, or exercise combined with liraglutide (Supplementary Figure S1 and Table S1).*"

And in the Methods section, lines 174-176:

"On completion of the 8-week low-calorie diet, only participants who lost at least 5% of their initial body weight were eligible for enrolment and subsequently randomized in this study."

Reproducibility of doppler USS
There are no Bland-Altman plots to show reproducibility. Reproducibility may not have been done but then the authors should state this in the limitations.

Authors' response:

Thank you for this comment. The cIMT was measured by trained personnel, and in accordance with the principles stated here,[10] which yielded a low coefficient of variation (around 5%) prior to the initiation of the trial. We have now included this as a limitation in the discussion.

Added to Limitations paragraph, lines 119-121:

"The ultrasound assessments were performed by personnel trained under a protocol that yielded a low coefficient of variation (around 5%) prior to the initiation of the trial."

The reviewer also feels that the use of carotid USS needs a little more explanation in the discussion. Why was this modality selected? The use of carotid USS has no RCT data to show its efficacy in the primary prevention of cardiovascular disease and this should be stated.

Authors' response:

We acknowledge the need to clarify the use of cIMT in the trial.

We have elaborated on the outcome in the Methods section, lines 238-241:

"Intima-media thickness of the common carotid artery provides a validated, reproducible, and non-invasive measure of subclinical atherogenic burden that is particularly informative in asymptomatic, middle-aged individuals with obesity and without previously established atherosclerotic disease.[9]"

We have also expanded the Discussion paragraph, lines 96-99:

"*Together with the observed decrease in cIMT captured by ultrasound, our findings suggest that structural vascular adaptations are more sensitive to exercise-induced hemodynamic and anti-inflammatory effects than to liraglutide's effects on metabolism alone during weight loss maintenance.[14]*"

And in the Limitations paragraph, lines 121-122:

"Additionally, cIMT is a surrogate endpoint regarding vascular health.[2]"

Exercise intervention

Participants randomised to exercise were subject to several support strategies that those randomised to nothing or liraglutide did not receive. These patients, by the nature of the study protocol, had more contact with researchers and this in as of itself may have led to a Hawthorne effect.

Authors' response:

We acknowledge that this might have affected the adherence and have included this in the Limitation paragraph, lines 125-128:

"Participants in the exercise groups had more frequent contact with study personnel than those in the placebo and liraglutide groups, which could have led to a Hawthorne effect; nevertheless, adherence to study medication was similar across all groups."

Circulating biomarkers

The measurement of several biomarkers is a strength of the study. The reviewer would suggest that the paper could be made stronger by including the coefficient of variation, limit of detection, and limit of quantification each biomarker in a supplementary table. The reviewer feels that a little more discussion on why the changes observed in the conduct of this study are beneficial to participants is warranted to strengthen the study findings and highlight potential relevance to clinical practice.

Authors' response:

Thank you for this comment. We have now included a table with coefficient of variation, limit of detection, and limit of quantification of each biomarker in *Supplementary Table S7:*

Variable	Inter-assay CV (%)	LLOD	LLOQ
IL-6 (pg/mL)[a]	7.3	0.06	0.633
IL-8 (pg/mL) [a]	7.1	0.07	0.591
IL-10 (pg/mL) [a]	10.1	0.04	0.298
IFN-gamma (pg/mL) [a]	7.3	0.37	1.76
TNF-alfa (pg/mL) [a]	10.1	0.04	0.690
sVCAM (pg/mL) [a]	8.4	6.00	37.6
sICAM (pg/mL) [a]	13.4	1.94	15.0
tPA (ng/mL)	5	0.31	0.75
vWF (%)	6	3.7	12.4
Inter-assay coefficient of variation in percent (CV) is the variability across different plates (usually 96-well plates) used for analyzing various biomarkers. LLOD: Lower limit of detection is defined as the calculated concentration corresponding to the average signal 2.5 standard deviations above background signaling. LLOQ: Lower limit of quantification: For [a] is defined as the lowest concentration (of			

the control samples) where the CVs of the calculated concentration were below 20% and where recovery of the samples was within 80-120% of the known value of the standard across all used plates. For tPA and vWF, it was defined as the average signal 10 standard deviations above background signaling. [a]Numbers provided by the manufacturer of the kits (MSD).

Further, we now discuss the beneficial impact of the observed changes in biomarkers and their potential relevance, lines 93-99:

"Our findings underscore the importance of incorporating and adhering to physical activity as an adjunct to GLP-1-based therapy for obesity, as it promotes healthy weight maintenance with reduced levels of pro-inflammatory cytokines IL-6 and IFN-γ and endothelial function markers sICAM-1, sVCAM-1, and tPA. Together with the observed decrease in cIMT captured by ultrasound, our findings suggest that structural vascular adaptations are more sensitive to exercise-induced hemodynamic and anti-inflammatory effects than to liraglutide's effects on metabolism alone during weight loss maintenance.[14]"

Statistical analysis
Power calculation appears to be missing here. What was the level of power? How was this calculated.

Authors' response:

Thank you for this consideration. The original study was powered to detect a 4 kg difference in body weight between any of the groups. To achieve a power of 80% at a significance level of 5%, 30 participants per group were estimated to be required to complete the study. This study concerns prespecified secondary outcomes, and the study was not powered to detect differences in these outcomes.

We now state this in the Methods section, lines 284-286:

"In the original trial, it was estimated that 30 participants in each group needed to complete the study to detect a 4 kg difference in weight between the intervention groups with a power of 80% at a significance level of 5%.[14]"

… and mention it as a limitation, lines 118-119:

"This is a prespecified secondary analysis, and findings should be interpreted with appropriate caution regarding type I error."

Table 1
Sex needs percent added to this

Authors' response:

We have added the percentage of sex to the table.

How was blood pressure recorded – in office or at home? Was this averaged over three readings?

Authors' response:

Thank you for this relevant comment, which is important for interpreting the values. We have described the method for blood pressure measurements in the Methods section, lines 262-264:

"Office blood pressure was measured twice in a seated position after at least 5 minutes of rest with an automated sphygmomanometer, and the average of the recordings is provided."

Summary
Overall this is an interesting and well conducted piece of work. The comments and suggestions have been provided to improve the scientific rigor of the paper and to tease out interesting comments.

Authors' response: Thank you very much. We greatly appreciate your time and constructive feedback.

References

1. Zyriax, B.C., Dransfeld, K. & Windler, E. Carotid intima-media thickness and cardiovascular risk factors in healthy volunteers. *Ultrasound J* **13**, 17 (2021).
2. Willeit, P.*, et al.* Carotid Intima-Media Thickness Progression as Surrogate Marker for Cardiovascular Risk. *Circulation* **142**, 621-642 (2020).
3. Stein, J.H.*, et al.* Use of Carotid Ultrasound to Identify Subclinical Vascular Disease and Evaluate Cardiovascular Disease Risk: A Consensus Statement from the American Society of Echocardiography Carotid Intima-Media Thickness Task Force Endorsed by the Society for Vascular Medicine. *Journal of the American Society of Echocardiography* **21**, 93-111 (2008).
4. Boughanem, H.*, et al.* Mediterranean diet, neutrophil count, and carotid intima-media thickness in secondary prevention: the CORDIOPREV study. *European Heart Journal* **46**, 719-729 (2024).
5. Lincoff, A.M.*, et al.* Semaglutide and Cardiovascular Outcomes in Obesity without Diabetes. *New England Journal of Medicine* **389**, 2221-2232 (2023).
6. Jensen, S.B.K.*, et al.* Healthy weight loss maintenance with exercise, GLP-1 receptor agonist, or both combined followed by one year without treatment: a post-treatment analysis of a randomised placebo-controlled trial. *EClinicalMedicine* **69**, 102475 (2024).
7. Wilding, J.P.H.*, et al.* Once-Weekly Semaglutide in Adults with Overweight or Obesity. *New England Journal of Medicine* **384**, 989-1002 (2021).
8. Pi-Sunyer, X.*, et al.* A Randomized, Controlled Trial of 3.0 mg of Liraglutide in Weight Management. *The New England journal of medicine* **373**, 11-22 (2015).
9. Stein, J.H.*, et al.* Use of carotid ultrasound to identify subclinical vascular disease and evaluate cardiovascular disease risk: a consensus statement from the American Society of Echocardiography Carotid Intima-Media Thickness Task Force. Endorsed by the Society for Vascular Medicine. *J Am Soc Echocardiogr* **21**, 93-111; quiz 189-190 (2008).
10. Potter, K.*, et al.* Carotid intima-medial thickness measured on multiple ultrasound frames: evaluation of a DICOM-based software system. *Cardiovasc Ultrasound* **5**, 29 (2007).
11. Sandsdal, R.M.*, et al.* Combination of exercise and GLP-1 receptor agonist treatment reduces severity of metabolic syndrome, abdominal obesity, and inflammation: a randomized controlled trial. *Cardiovasc Diabetol* **22**, 41 (2023).
12. Shivgulam, M.E.*, et al.* Impact of Exercise Training Interventions on Flow-Mediated Dilation in Adults: An Umbrella Review. *Sports Medicine* **53**, 1161-1174 (2023).
13. Gleeson, M.*, et al.* The anti-inflammatory effects of exercise: mechanisms and implications for the prevention and treatment of disease. *Nat Rev Immunol* **11**, 607-615 (2011).
14. Lundgren, J.R.*, et al.* Healthy Weight Loss Maintenance with Exercise, Liraglutide, or Both Combined. *The New England journal of medicine* **384**, 1719-1730 (2021).
15. Tousoulis, D., Antoniades, C., Vlachopoulos, C. & Stefanadis, C. Flow mediated dilation and carotid intima media thickness: clinical markers or just research tools? *Int J Cardiol* **163**, 226-228 (2013).
16. Hogan, A.E.*, et al.* Glucagon-like peptide 1 analogue therapy directly modulates innate immune-mediated inflammation in individuals with type 2 diabetes mellitus. *Diabetologia* **57**, 781-784 (2014).
17. von Scholten, B.J.*, et al.* Effects of liraglutide on cardiovascular risk biomarkers in patients with type 2 diabetes and albuminuria: A sub-analysis of a randomized, placebo-controlled, double-blind, crossover trial. *Diabetes, Obesity and Metabolism* **19**, 901-905 (2017).
18. Rajkovic, N.*, et al.* Relationship between Obesity, Adipocytokines and Inflammatory Markers in Type 2 Diabetes: Relevance for Cardiovascular Risk Prevention. *International Journal of Environmental Research and Public Health* **11**, 4049-4065 (2014).

Reviewer #1 (Remarks to the Author):

In this revised manuscript, now presented as a brief communication, Sandsdal and colleagues report the findings of a 2x2 factorial trial of exercise and liraglutide on changes in carotid IMT. The authors are thanked for the substantial changes that have been made and responses to the reviewer comments. It makes this much easier to understand and review. The main findings, that exercise interventions, but not liraglutide, has favorable effects on changes in cIMT are clear. The authors should consider a number of points.

Authors' response:

We thank Reviewer #1 for the constructive comments across both rounds of review and are pleased that the revised manuscript has improved in clarity. We have addressed the remaining points below.

1. My main concern remains the study design. The run of weight loss for all is problematic for the interpretation of all subsequent findings, and as pointed out in my previous commentary, is artificial compared with the real world.

Authors' response:

We thank the reviewer for highlighting clarification on this point and have elaborated on the limitations section paragraph, line 149.

"*While initial diet-induced weight loss is used in some clinical practices, including in Denmark and the United Kingdom,*[1-3] *GLP-1–based therapy is commonly prescribed as an adjunct to diet and exercise recommendations. Thus, in this study, the initial weight loss may have attenuated weight loss–dependent effects of liraglutide and exercise. However, the main outcome, cIMT, did not improve with the LCD-induced weight loss, but only with one year of exercise with or without liraglutide.*"

2. The interpretation that the findings suggest that GLP-1 receptor agonists effects on cardiovascular health must be mediated via weight loss on the basis of the lack of findings on cIMT should be toned down. I'm not convinced this study provides any mechanistic insights and given the pre-existing weight loss, it is impossible to interpret this. It also contrasts with findings of large cardiovascular outcomes trials in the GLP-1 space, in which reported benefits appear earlier than substantial weight loss, arguing the opposite point from the authors of this manuscript.

Authors' response:

We agree with the reviewer and have revised the point regarding the mediation of GLP-1 RAs' effects on cardiovascular health and have focused on the findings on the improvement of cIMT with or without liraglutide after initial weight loss.

The revised passage in the Discussion paragraph, line 126, now reads:

From:

"*Liraglutide maintained the LCD-induced weight loss but did not reduce cIMT in this study. This contrasts with previous clinical trials showing cIMT reduction with GLP-1 RA treatment; however, those studies examined patients with type 2 diabetes, a population characterized by substantially higher baseline inflammation and cardiovascular risk, and did not include an initial standardized weight loss phase. In the present study, all participants achieved approximately 13% weight loss through the LCD before randomization, which liraglutide then maintained throughout the intervention period. This design allows for assessment of GLP-1 RA effects independent of ongoing weight change. Our findings indicate that in people with obesity but without diabetes and with no established cardiovascular disease who have already achieved substantial weight loss, liraglutide provides limited additional benefit for reducing cardiovascular risk markers unless combined with exercise.*"

To:

"*One-year treatment with liraglutide alone did not reduce cIMT while maintaining the diet-induced weight loss. This contrasts with previous clinical trials showing cIMT reduction with GLP-1 RA treatment; however, these studies examined patients with type 2 diabetes, a population characterized by higher baseline cIMT, and did not include an initial standardized weight loss phase. Also, the large cardiovascular outcome trial with the GLP-1 RA semaglutide in patients with established cardiovascular disease has shown reduced incidence of cardiovascular events.[4] Our findings should be interpreted as pertaining to the weight loss maintenance phase in people with obesity who do not have diabetes or established cardiovascular disease and have already achieved substantial weight loss. In this context, liraglutide did not provide additional benefit in reducing cIMT and pro-inflammatory cytokines without exercise.*"

We have also revised the conclusion and emphasized the initial weight loss, line 167:

From:

"*In conclusion, exercise with and without liraglutide treatment during weight loss maintenance reduced carotid intima-media thickness and pro-inflammatory cytokine levels, and the combination of exercise and liraglutide also improved endothelial function in adults with obesity, whereas treatment with liraglutide alone did not. Thus, regular physical activity, with or without GLP-RA, is essential for promoting vascular health in adults with obesity, rather than GLP-RA treatment alone.*"

To:

"*In conclusion, among individuals with obesity who had adhered to a low-calorie diet, exercise reduced carotid intima-media thickness and pro-inflammatory cytokines, and exercise in combination with GLP-1 RA liraglutide also improved endothelial function. This was not observed with liraglutide treatment alone or with placebo, highlighting the role of physical activity in improving vascular health in adults with obesity.*"

3. Triglycerides should be presented as median (interquartile range).

Authors' response:

We now present triglycerides as the median with IQR.

4. It is surprising that hsCRP was not measured, when a number of inflammatory markers were included.

Authors' response:

We agree and have now included hsCRP in Supplementary Tables S1 and S3. In summary, changes in hsCRP were similar to those seen in the other inflammatory markers.

Reviewer #2 (Remarks to the Author):

I thank the editors and authors for their responses. No further comments

Authors' response:

We thank the reviewer for their time and for reviewing our manuscript.

Reviewer #3 (Remarks to the Author):

Many thanks to the Editorial Board of Nature Metabolism for asking me to review this paper further. The reviewer would like to thank the authors for their considerable changes to the manuscript and once more congratulate them on an excellent study to help understand the effects of GLP-1 receptor analogues and exercise on cardiovascular health.

Authors' response:

We thank the reviewer for their generous comments and constructive engagement throughout the review process, which have greatly improved the manuscript.

The reviewer makes a number of minor suggestions that can be applied at the discretion of the authors and editorial board.

The title should not provide the outcome. Suggest: "The effects of exercise and liraglutide on vascular health and inflammation during weight loss: a prespecified secondary analysis of the S-LiTE trial".

Authors' response:

Thank you for the suggestion. We have now revised the title to:

"*Effects of exercise and liraglutide on vascular health and inflammation during weight loss maintenance: a prespecified secondary analysis of the S-LiTE trial*"

Whilst this is a pre-specified secondary analysis, the reviewer thinks the (primary) objective of this study should be clearer. The authors state they investigated changes

in cIMT and circulating biomarkers. This reviewer believe they did in fact evaluate the effects of exercise and liraglutide on cIMT, inflammatory cytokines and endothelial cell function.
Suggest the final sentence of the introductory paragraph read something similar to: Therefore, we aimed to determine the effects of liraglutide and exercise, either alone or in combination, on cIMT, circulating pro-inflammatory cytokines and endothelial biomarkers in a prespecified secondary analysis of the S-LiTE randomized placebo-controlled trial to elucidate distinct mechanisms on vascular health of exercise.

Authors' response:

We agree and have clarified the objective in the Introduction paragraph accordingly, line 59.

From:

"*Therefore, we investigated changes in cIMT and circulating inflammatory and endothelial biomarkers in a prespecified secondary analysis of the S-LiTE randomized placebo-controlled trial to elucidate distinct mechanisms on vascular health of exercise, GLP-1 RA, and the combination treatment.*"

To:

"*Therefore, we aimed to determine the effects of liraglutide and exercise, either alone or in combination, on cIMT, circulating pro-inflammatory cytokines, and endothelial function in a prespecified secondary analysis of the S-LiTE randomized placebo-controlled trial to elucidate distinct mechanisms on vascular health.*"

In line 57 the description of exercise should either be expressed as a % of the population achieving adherence to guideline recommendations for exercise or state the average/median minutes of exercise per week i.e. In our study, X% of individuals in the exercise only group achieved guideline recommendation and exercised for X minutes +/- Y minutes as compared to x% in those randomized to liraglutide and exercise, who managed x minutes +/- y minutes.

Authors' response:

We thank the reviewer for this suggestion. We have added this in the manuscript, line 75:

From:

"*Exercise adherence was 156 ± 54 min/week at 78 ± 4% maximum heart rate in the exercise-only group and 144 ± 67 min/week at 78 ± 5% maximum heart rate in the combination group.*"

To:

"*Exercise adherence was 156 ± 54 min/week at 78 ± 4% maximum heart rate in the exercise-only group (corresponding to 92% achieving the minimum WHO recommendations on physical activity) and 144 ± 67 min/week at 78 ± 5% maximum heart rate in the combination group (corresponding to 79% achieving the minimum WHO recommendations on physical activity).*"

Line 70 states that the authors determined whether the changes in the macroscopic changes in vascular structure were associated with concurrent reductions in systemic inflammation. There are no data correlating reductions in cIMT with systemic inflammation. I think the authors mean they are evaluating the effects of the intervention on these circulating biomarkers.

Authors' response:

We agree and have clarified the sentence accordingly, line 90:

From:

"*We then investigated whether the macroscopic changes in vascular structure were concurrent with reductions in systemic inflammation biomarkers IL-6, TNF-α, and IFN-γ, the most upstream circulating markers of atherosclerotic development*."

To:

"*We then investigated the effects of exercise, liraglutide, or the combination treatment on systemic inflammation biomarkers IL-6, TNF-α, and IFN-γ, the most upstream circulating markers of atherosclerotic development.*"

The reviewer would like to thank the authors for incorporating the level of detection, level of quantification and coefficient of variation with the supplement.

Authors' response:

We thank the reviewer for the feedback.

The conclusion should be sharper. Suggest something along the lines of:
In conclusion, individuals with obesity who adhered to a low-calorie diet, exercise alone and in combination with liraglutide was shown to reduce carotid intima-medial thickness, pro-inflammatory cytokines and improve endothelial function. This was not observed with liraglutide alone or with placebo, highlighting the role of physical activity in improving vascular health in adults with obesity.

Authors' response:

We thank the reviewer for this suggestion and have sharpened the conclusion, line 167.

From:

"*In conclusion, exercise with and without liraglutide treatment during weight loss maintenance reduced carotid intima-media thickness and pro-inflammatory cytokine levels, and the combination of exercise and liraglutide also improved endothelial function in adults with obesity, whereas treatment with liraglutide alone did not. Thus, regular physical activity, with or without GLP-RA, is essential for promoting vascular health in adults with obesity, rather than GLP-RA treatment alone.*"

To:

"*In conclusion, among individuals with obesity who had adhered to a low-calorie diet, exercise reduced carotid intima-media thickness and pro-inflammatory cytokine levels, and the combination of exercise and liraglutide also improved endothelial function. This was not observed with liraglutide treatment alone or with placebo, highlighting the role of regular physical activity in improving vascular health in adults with obesity.*"

References

1. Astbury, N.M., *et al.* Doctor Referral of Overweight People to Low Energy total diet replacement Treatment (DROPLET): pragmatic randomised controlled trial. *BMJ* **362**, k3760 (2018).
2. Lean, M.E.J., *et al.* Durability of a primary care-led weight-management intervention for remission of type 2 diabetes: 2-year results of the DiRECT open-label, cluster-randomised trial. *The Lancet Diabetes & Endocrinology* **7**, 344-355 (2019).
3. Valabhji, J., *et al.* Early findings from the NHS Type 2 Diabetes Path to Remission Programme: a prospective evaluation of real-world implementation. *The Lancet Diabetes & Endocrinology* **12**, 653-663 (2024).
4. Lincoff, A.M., *et al.* Semaglutide and Cardiovascular Outcomes in Obesity without Diabetes. *New England Journal of Medicine* **389**, 2221-2232 (2023).

Many thanks to the Editorial Board of Nature Metabolism for asking me to review this paper further. The reviewer would like to thank the authors for their considerable changes to the manuscript and once more congratulate them on an excellent study to help understand the effects of GLP-1 receptor analogues and exercise on cardiovascular health.

The reviewer makes a number of minor suggestions that can be applied at the discretion of the authors and editorial board.

The title should not provide the outcome. Suggest: "The effects of exercise and liraglutide on vascular health and inflammation during weight loss: a prespecified secondary analysis of the S-LiTE trial".

Whilst this is a pre-specified secondary analysis, the reviewer thinks the (primary) objective of this study should be clearer. The authors state they investigated changes in cIMT and circulating biomarkers. This reviewer believe they did in fact evaluate the effects of exercise and liraglutide on cIMT, inflammatory cytokines and endothelial cell function.

Suggest the final sentence of the introductory paragraph read something similar to:
Therefore, we aimed to determine the effects of liraglutide and exercise, either alone or in combination, on cIMT, circulating pro-inflammatory cytokines and endothelial biomarkers in a prespecified secondary analysis of the S-LiTE randomized placebo-controlled trial to elucidate distinct mechanisms on vascular health of exercise.

In line 57 the description of exercise should either be expressed as a % of the population achieving adherence to guideline recommendations for exercise or state the average/median

minutes of exercise per week i.e. In our study, X% of individuals in the exercise only group achieved guideline recommendation and exercised for X minutes +/- Y minutes as compared to x% in those randomized to liraglutide and exercise, who managed x minutes +/- y minutes.

Line 70 states that the authors determined whether the changes in the macroscopic changes in vascular structure were associated with concurrent reductions in systemic inflammation. There are no data correlating reductions in cIMT with systemic inflammation. I think the authors mean they are evaluating the effects of the intervention on these circulating biomarkers.

The reviewer would like to thank the authors for incorporating the level of detection, level of quantification and coefficient of variation with the supplement.

The conclusion should be sharper. Suggest something along the lines of:
In conclusion, individuals with obesity who adhered to a low-calorie diet, exercise alone and in combination with liraglutide was shown to reduce carotid intima-medial thickness, pro-inflammatory cytokines and improve endothelial function. This was not observed with liraglutide alone or with placebo, highlighting the role of physical activity in improving vascular health in adults with obesity.
